# δ^13^C as a tool for iron and phosphorus deficiency prediction in crops

**DOI:** 10.1002/pld3.487

**Published:** 2023-03-20

**Authors:** Fabio Trevisan, Raphael Tiziani, Robert D. Hall, Stefano Cesco, Tanja Mimmo

**Affiliations:** ^1^ Faculty of Science and Technology Free University of Bolzano Bolzano Italy; ^2^ Laboratory of Plant Physiology Wageningen University & Research Wageningen The Netherlands; ^3^ Business Unit Bioscience Wageningen University & Research Wageningen The Netherlands; ^4^ Competence Centre of Plant Health Free University of Bozen‐Bolzano Bolzano Italy

**Keywords:** barley, C4 and C3 plants, carbon isotope ratio, cucumber, fractionation, maize, nutrient deficiency, plant physiology, tomato

## Abstract

Many studies proposed the use of stable carbon isotope ratio (δ^13^C) as a predictor of abiotic stresses in plants, considering only drought and nitrogen deficiency without further investigating the impact of other nutrient deficiencies, that is, phosphorus (P) and/or iron (Fe) deficiencies. To fill this knowledge gap, we assessed the δ^13^C of barley ( *Hordeum vulgare* L.), cucumber ( *Cucumis sativus* L.), maize ( *Zea mays* L.), and tomato (*Solanum lycopersicon* L.) plants suffering from P, Fe, and combined P/Fe deficiencies during a two‐week period using an isotope‐ratio mass spectrometer. Simultaneously, plant physiological status was monitored with an infra‐red gas analyzer. Results show clear contrasting time‐, treatment‐, species‐, and tissue‐specific variations. Furthermore, physiological parameters showed limited correlation with δ^13^C shifts, highlighting that the plants' δ^13^C, does not depend solely on photosynthetic carbon isotope fractionation/discrimination (Δ). Hence, the use of δ^13^C as a predictor is highly discouraged due to its inability to detect and discern different nutrient stresses, especially when combined stresses are present.

## 1 INTRODUCTION

Plants require essential mineral nutrients to survive and successfully complete their life cycle (Marschner, 2012). Under optimal conditions, all essential nutrients and chemical species are present with the right amount in the growth medium. However, nutrient shortages of any type and magnitude are widely diffused around the globe and reduce crop yield, both in terms of quantity and quality (MacDonald et al., 2011; Samaranayake et al., 2012). Nitrogen (N), phosphorus (P) and iron (Fe) deficiencies represent three of the major agricultural production limitations worldwide (Samaranayake et al., 2012; van de Wiel et al., 2016; Zuo & Zhang, 2011). To be more precise, N alone is considered a limiting factor for agriculture in approx. 7%–64% of cultivated land (Thornton et al., 2007; Wang et al., 2007). Moreover, degraded acidic and calcareous soils (50% and 25% of the world's arable land, respectively) have a high incidence on P and Fe availability (Fisher et al., 2012; Lynch, 2011; Zheng, 2010). Despite the differences between N, P, and Fe in terms of soil bioavailability, uptake, storage, and mobility within the plant, shortages of any of these three essential nutrients induce morpho‐physiological adaptations in plants. These responses comprise root elongation, root hair development, metabolic re‐programming, reduced photosynthetic rate, and/or stomata closure (Meng et al., 2021; Wei et al., 2016; Zhang et al., 2014), ultimately causing shifts in the carbon isotope discrimination or fractionation (Δ) and consequently in the plant's stable carbon isotope ratio (δ^13^C) (Cernusak et al., 2013; Farquhar & Richards, 1984; Tcherkez, 2010).

Fujita et al. (2003) and Tiziani et al. (2020) observed that the δ^13^C of P deficient tomato plants was higher as compared to plants grown in nutrient sufficiency. These authors suggested that the reduction in stomatal conductance, associated in many plant species with P deficiency (Zhang et al., 2014), is the main cause of the δ^13^C increase. Indeed, the reduced air exchange between the external environment and the intercellular air spaces decreases the available CO_2_ for fixation, obliging ribulose‐1,5‐bisphosphate carboxylase‐oxygenase (RuBisCO) to use the accessible CO_2_, including ^13^CO_2_ and consequently decreasing the discrimination against ^13^C. The reduced Δ ultimately results in an increase of the plant δ^13^C (Boyer & Kawamitsu, 2011). Instead, lower δ^13^C has been observed in Fe‐depleted sugar beet by Rombolà et al. (2005). These results are in contrast with a well‐known increase in the root phosphoenolpyruvate carboxylase (PEPC) activity in the same nutrient deficient plants (Andaluz et al., 2002; López‐Millán et al., 2009), considering that the PEPC fixes carbon with low Δ (2.2‰–3.0‰ of PEPC vs. 29‰–30‰ of RuBisCO) (Cernusak et al., 2013; Tcherkez et al., 2011; von Caemmerer et al., 2014). This inconsistency—a higher PEPC activity but lower δ^13^C—may be explained by a reduction in RuBisCO activity, resulting in an increase in CO_2_ concentration at the RuBisCO carboxylation site with a consequent increase of the discrimination against ^13^C. In addition, in C4 plant species, the effect of RuBisCO's fractionation might be suppressed due to their semi‐closed bundle sheath cells, their PEPC, the primary carboxylating enzyme in C4 plants, having a lower Δ, and their reduced tendency to close stomata in response to abiotic stresses (Bowman et al., 1989; Henderson et al., 1998).

Due to this existing evidence, δ^13^C measurements were proposed to identify, quantify, and describe abiotic stress dynamics. Indeed, δ^13^C might be exploited as early diagnosis tool to detect latent stress signals in plants (Clay et al., 2001; Dercon et al., 2006; Pansak et al., 2007). In this regard, we hypothesized a different Δ in aboveground and underground tissues. In fact, it is well known that there might be strong δ^13^C differences between photosynthetic and non‐photosynthetic tissues due to different biochemical composition and temporal development, and more in general diverse use of C sources, for example, C fixed by PEPC, C derived from sucrose storages accumulated during the day versus the night (Cernusak et al., 2009). However, although the potential of δ^13^C to detect abiotic stresses has been tested for N, water shortages, and their interaction (Clay et al., 2001; Dercon et al., 2006; Pansak et al., 2007), its applicability for P, Fe, and their combined nutrient deficiencies remains untested. Moreover, many blind spots persist in the plant δ^13^C research area. For instance, we have no insights into δ^13^C responses to Fe deficiency in C4 plants. The same is valid for combined nutrient deficiencies, for example, P and Fe, in either C3 and C4 plants. This, together with the limited number of available Δ in key reactions of primary carbon metabolism, prevents the prediction of the δ^13^C in plants suffering from nutrient deficiencies (Tcherkez et al., 2011).

Accordingly, the aim of the present research was to investigate the relationship between δ^13^C and nutrient stresses, that is, P and Fe deficiencies, in four different plant species. In addition, to understand whether δ^13^C could be used as a predictor of abiotic stresses, we aimed to test the following research hypotheses: (i) plant δ^13^C is expected to vary both in a treatment‐ and tissue‐ specific manner; (ii) the impact of nutrient deficiency on δ^13^C is expected to be milder in C4 plants than in C3 plants; and (iii) plant species utilizing the same photosynthetic pathway or with the same number of cotyledons behave similarly.

## 2 MATERIAL AND METHODS

### 2.1 Plant species

Four plant species (*Solanum Lycopersicon* L. *cv* Marmande; *Cucumis sativus* L. *cv* Chinese Long; *Hordeum vulgare* L*. cv* Solist; *Zea mays* L. *cv* F1 Hybrid P0423, Pioneer Hi‐Bred Italia S.r.l) were chosen considering photosynthesis type (C3 and C4) as well as Fe acquisition strategy (I and II) and crop physiology (monocot and dicot) (Table S1).

### 2.2 Growing conditions

All the seeds of the different plant species were germinated in plastic boxes containing 0.5 mmol L^−1^ CaSO_4_ moistened tissue paper (Nikolic et al., 2012). Darkness and a constant temperature of 25°C were maintained for 4–10 days according to the plant species (barley 4 days, cucumber 6 days, maize 10 days, tomato 7 days). After this germination period, the young seedlings were transferred into 1.5 L plastic pots, 10 seedlings per pot, containing species‐specific nutrient solutions (NSs) according to Marastoni et al. (2019) (Table S2). Synthetic sponges wrapped around the root collar were used as structural support, whereas the root system grew into the NS without any substrate. Particular attention has been paid to the selection of uniform seedlings to reduce biological variation as much as possible. The aerated NS was renewed every 3 days.

After 7 days of growth in a complete NS, 75% of the plants (min 105 plants per species) were transferred to treatment‐specific NSs for the remaining 14 days, whereas the remaining 25% (min 35 plants per species) were kept in a complete NS as a control. More precisely, 25% of the plants (min 35 plants per species) were grown under phosphorus deficiency (−P), 25% (min 35 plants per species) under iron deficiency (−Fe), and the remaining 25% (min 35 plants per species) under a combined phosphorus and iron deficiency (−P/−Fe). Hence, for each plant species, a minimum of 140 plants (five replicates, four treatments, seven timepoints) were used in addition to 15 plants (five replicates, one treatment, three timepoints) for monitoring the δ^13^C‰ prior the onset of nutrient deficiencies, usually twice as much to have spare plants as backup. This adds up to a total of 1240 plants. For the whole period of plant growth (21 days), the environmental parameters of the growth chamber were kept constant with a light:dark cycle of 14:10 h, a temperature regime of 24°C:19°C, a relative humidity of 70%, and a light intensity of 75 μmol m^−2^ s^−1^ at plant level.

Plants were sampled starting from the onset of the nutrient stresses and thereafter every 2 to 3 days. The sampling consisted of the harvest of the whole plant and the separation of the root system from the shoot. Seven biological replicates for each sampling time and treatment condition were used in this experiment.

### 2.3 Carbon isotope ratio determination

The harvested tissues were dried at 70°C until constant weight was reached, approx. 3 days. Dried biomass was then ball‐milled with a Mixer Mill MM 400 (Retsch, Italy) for 6 min at an oscillation frequency of 30 Hz, and 0.25 mg of the homogenized dried samples was weighed and placed into tin capsules. Following weighing, the total combustion of the sample and the δ^13^C determination was performed using an elemental analyzer (EA Flash 1112 Thermo Scientific, Germany) coupled to a continuous flow isotope ratio mass spectrometer (Delta V Thermo Scientific, Germany). The working conditions consisted of 1020°C in the oxidation furnace and 900°C in the reduction furnace. The produced H_2_O was removed by a Mg (ClO_4_)_2_ trap. The stable carbon isotope ratios were expressed in δ‰ versus an international reference, that is, V‐PDB (Vienna–Pee Dee Belemnite), according to the following equation:

(1) $$\delta^{13}C‰=\frac{\left(R_{\text{sample}}-R_{\text{standard}}\right)}{R_{\text{standard}}},$$

where R is the ratio between the heavier and the lighter isotopes; R_sample_ is the carbon isotope ratio measured for the sample, either root or shoot tissue; and R_standard_ is the carbon isotope ratio measured for an international standard, that is, Vienna–Pee Dee Belemnite (Farquhar et al., 1989; Hayes, 2001).

For the quality control of the analysis, four series of three working standards, namely IAEA 600 (caffeine), IAEA CH_3_ (cellulose), and acetanilide, were analyzed at the beginning, during, and end of the sequence, respectively. The measurement uncertainty for carbon isotopic determination was ±0.2‰.

### 2.4 Analysis of physiological parameters

During the last sampling day, just before harvesting the plants, leaf CO_2_ and water vapor fluxes were assessed by an infra‐red gas analyzer (IRGA) equipped with a small leaf chamber (2.16 cm^2^) (ACD BioScientific Ltd. – Lcpro T). CO_2_ concentration and relative humidity under the leaf and from the reference (far away from any CO_2_ or H_2_O source), leaf temperature, photosynthetic active radiation (PAR) at leaf level, atmospheric pressure, and effective molar air flow were obtained, allowing for the calculation of photosynthetic rate, stomatal conductance, and transpiration rate. Multiple measurements on different biological replicates per each treatment were taken from fully expanded 15‐day old leaves. Gas exchanges were measured under common conditions: molar air flow rate (68 μmol s^−1^), boundary resistance (0.25 m^2^ s mol^−1^), ambient CO_2_ concentration (550 ppm), leaf temperature (27 ± 1.5°C), and light intensity (75 μmol m^−2^ s^−1^).

### 2.5 Statistical analysis

R x64 version 4.1.2 software and SigmaPlot 12 for Windows 10 64bit were used to statistically analyze the obtained raw data. The following R packages were used: ggplot2 for data visualization (Wickham, 2016) and agricolae for statistical analysis (de Mendiburu, 2021). All the results will be reported as means ± standard error. Multiple comparisons between different treatments have been performed through two‐way analysis of variance (ANOVA) and one‐way ANOVA with Tukey as a post hoc test and a significant *p*‐value < 0.05.

## 3 RESULTS

### 3.1 Stomatal conductance

**FIGURE 1:**
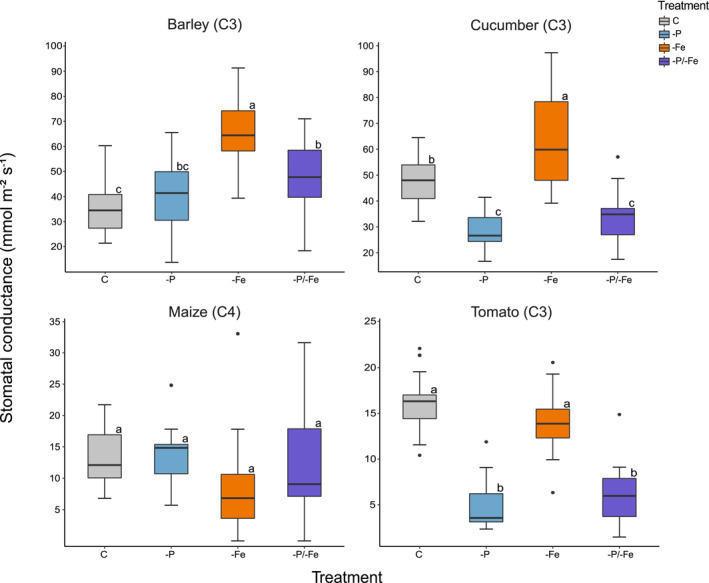
Leaf stomatal conductance (mmol m^−2^ s^−1^) of barley ( *Hordeum vulgare* L.), cucumber ( *Cucumis sativus* L.), maize ( *Zea mays* L.), and tomato (*Solanum lycopersicon* L.) plants according to four different treatments: control (C), phosphorus deficiency (−P), iron deficiency (−Fe), and phosphorus and iron combined deficiencies (−P/−Fe) with *n* ≥ 12. Letters next to the boxes indicate statistically significant differences (*p* < 0.05) assessed by a one‐way ANOVA with Tukey post hoc test.

Maize stomatal conductance was unaffected by the applied nutrient deficiencies (Figure 1). On the contrary, barley and cucumber plants suffering from Fe deficiency showed a significantly higher stomatal conductance (+57% and +71%, respectively) as compared to all other treatments. Despite this similarity, in barley, −P/−Fe induced a significantly higher stomatal conductance (+33%) than the control, whereas in cucumber, −P and −P/−Fe were found to negatively influence stomatal conductance (−34%) relative to the control. Similarly, in tomato, −P and −P/−Fe induced a significantly lower stomatal conductance in comparison with both −Fe and the control (−64%) (Figure 1).

### 3.2 Photosynthetic rate

**FIGURE 2:**
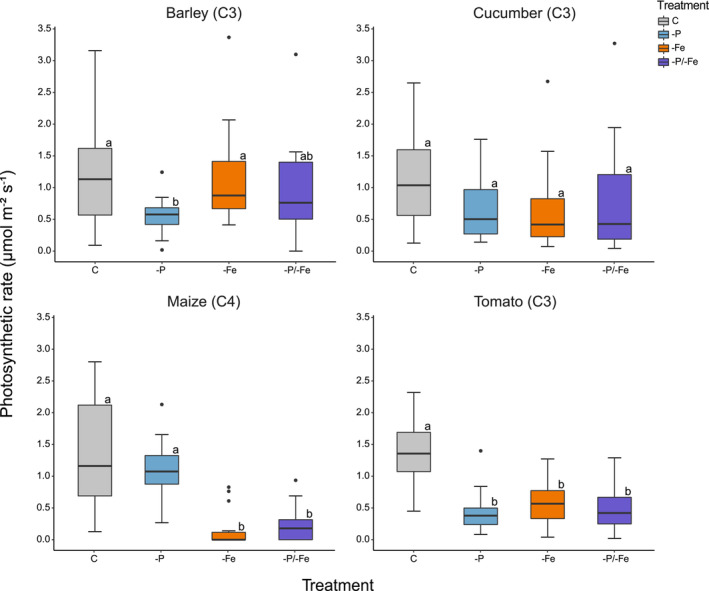
Leaf photosynthetic rate (μmol m^−2^ s^−1^) of barley ( *Hordeum vulgare* L.), cucumber ( *Cucumis sativus* L.), maize ( *Zea mays* L.), and tomato (*Solanum lycopersicon* L.) plants according to four different treatments: control (C), phosphorus deficiency (−P), iron deficiency (−Fe), and phosphorus and iron combined deficiencies (−P/−Fe) with *n* ≥ 12. Letters next to the boxes indicate statistically significant differences (*p* < 0.05) assessed by one‐way ANOVA with Tukey post hoc test.

Cucumber's photosynthetic rate was unaffected by the applied nutrient deficiencies. On the contrary, all treatments reduced tomato's photosynthetic rate as compared to the control (−64% on average). Similarly, maize subjected to Fe and P/Fe combined deficiencies showed a significantly lower photosynthetic rate (−87% and −82%, respectively) as compared to −P and control treatments, both of which had similar photosynthetic rates. Barley's photosynthetic rate was negatively influenced by P deficiency (−53% on average) as compared to the control or −Fe but was not significantly different to the −P/−Fe treatment (Figure 2).

### 3.3 Stable carbon isotope ratio (δ^13^C)

#### 3.3.1 Barley

**FIGURE 3:**
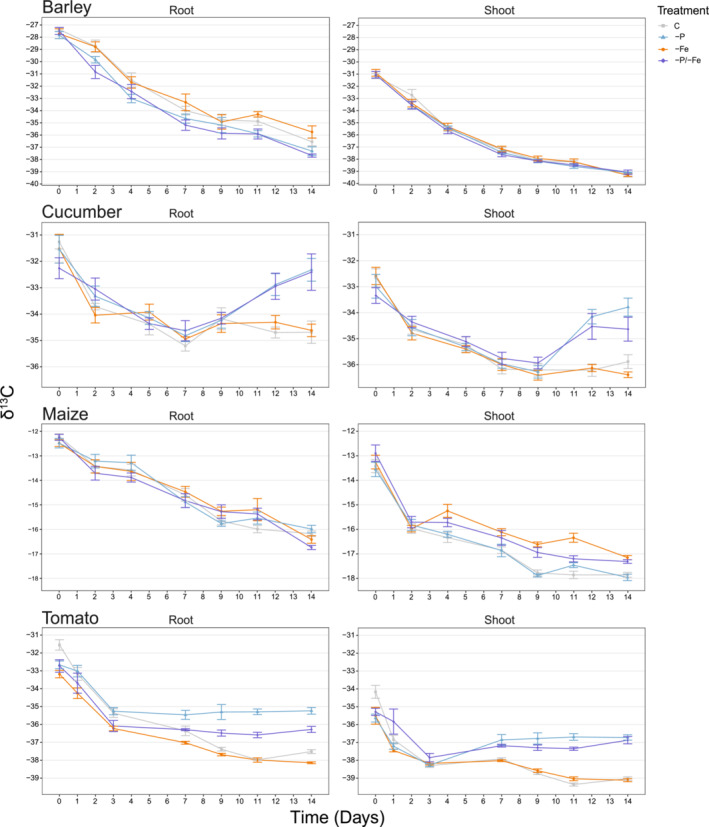
δ^13^C values for barley ( *Hordeum vulgare* L.), cucumber ( *Cucumis sativus* L.), maize ( *Zea mays* L.), and tomato (*Solanum lycopersicon* L.) plants grown under control conditions (C), phosphorus starvation (−P), iron starvation (−Fe), and combined phosphorus and iron starvation (−P/−Fe) plotted over time. Day 0 on the x‐axis indicates the day on which some of the plants were transferred to the treatment‐specific nutrient solutions (NSs), that is, after 7 days of growth in full NS. The δ^13^C values of roots, left, and shoots, right, are represented as mean ± standard error, *n* = 7.

Two‐way ANOVA highlighted that barley δ^13^C was significantly influenced by time (*p* < 0.001) and treatments (*p* < 0.001) for roots and only by time (*p* < 0.001) for shoots (Table 1). However, there was no interaction observed between the two factors in either of the two analyzed plant tissues (*p* = 0.504 root; *p* = 0.670 shoot). Barley δ^13^C significantly decreased in both roots and shoots for the whole duration of the time course in all treatments, without reaching a stable δ^13^C value. Root δ^13^C decreased from −27.5‰ to −36.5‰ in the control (i.e., a reduction of 33%), to −37.5‰ in −P and −P/−Fe (−36%), and to −35.5‰ in −Fe (−29%) between Days 0 and 14. For shoots, instead, in the same time span, the δ^13^C decreased by 26% in all the treatments, from −31.0‰ to −39.0‰ (Figure 3).

**TABLE 1:** Results of the two‐way ANOVA on the δ^13^C data shown in Figure 3.

	Barley			Cucumber			Maize			Tomato		
	Df	*F* value	*P* value	Df	*F* value	*P* value	Df	*F* value	*P* value	Df	*F* value	*P* value
Root												
Treatment	3	15.05	<0.001	3	7.06	<0.001	3	0.72	0.543	3	61.48	<0.001
Time	6	249.08	<0.001	6	31.77	<0.001	6	149.17	<0.001	6	210.85	<0.001
Treatment:Time	18	0.96	0.504	18	2.76	<0.001	18	1.29	0.202	18	6.55	<0.001
Residuals	168			168			168			168		
Shoot												
Treatment	3	1.52	0.212	3	10.26	<0.001	3	27.18	<0.001	3	47.80	<0.001
Time	6	768.39	<0.001	6	63.08	<0.001	6	231.87	<0.001	6	87.47	<0.001
Treatment:Time	18	0.82	0.670	18	4.41	<0.001	18	1.92	0.017	18	9.80	<0.001
Residuals	168			168			168			168		

Abbreviation: Df = degrees of freedom.

Even though the treatment effects were significant at root level, no clear trend nor pattern can be recognized. Iron deficiency revealed significantly higher δ^13^C values as compared to −P and −P/−Fe (+5%) in the last three growing days but not as compared to the control (Table S3). No significant differences were instead identified among control, −P, and −P/−Fe. For shoots, all the treatments behaved in the same way across the time course (Figure 3).

#### 3.3.2 Cucumber

The two‐way ANOVA highlighted highly significant time and treatment impacts on cucumber roots and shoots δ^13^C (*p* < 0.001). Moreover, there was also significant interaction between the two factors in both the analyzed plant tissues (*p* < 0.001, Table 1), indicating that the effect of nutrient deficiencies on the cucumber δ^13^C and their relationship varied over time (Figure 3). In all four treatments, the δ^13^C decreased up to Day 7 in roots (from −31.6‰ to −34.9‰, i.e., −10% on average) and to Day 9 in shoots (from −32.9‰ to −36.2‰, i.e., −10% on average). Despite the similar behavior of all treatments in the first half of the time course, the δ^13^C of both tissues suffering from −P and −P/−Fe deficiency later increased until Day 14, reaching −32.4‰ in roots (+7%) and −34.2‰ in shoots (+5%). On the contrary, the δ^13^C of the control and −Fe plants stabilized after Day 7 in roots (approx. 34.6‰) and after Day 9 in shoots (approx. 36.2‰) (Table S3). Indeed, −P and −P/−Fe showed a significant increasingly higher plant δ^13^C as compared to −Fe and the control from Day 9 onwards (+5% Day 12 and +7% Day 14 in roots and +5% Days 12 and 14 in shoots) (Figure 3).

#### 3.3.3 Maize

The two‐way ANOVA highlighted that time significantly altered maize roots (*p* < 0.001) and shoots (*p* < 0.001) δ^13^C, whereas the nutrient deficiencies only had an impact on shoots (*p* < 0.001) δ^13^C. Furthermore, shoots also showed an interaction between the two variables (*p* = 0.017) (Table 1).

A significant decrease in maize δ^13^C was observed during the time course at both root and shoot levels. In roots, the δ^13^C of control and −P stabilized from Day 7 onwards, and it continued to decrease till the last sampling day in the −Fe and −P/−Fe treatments. At root level, between Days 0 and 14, δ^13^C dropped from −12.2‰ to −16.2‰ in the control (−33%), from −12.5‰ to −16.0‰ in −P (−28%), from −12.5‰ to −16.5‰ in −Fe (−32%), and from −12.2‰ to −16.7‰ in −P/−Fe (−37%). In shoots, δ^13^C decreased between Days 0 and 14, from −13.5‰ to −18.0‰ in the control and −P (−33%), and from −13.1‰ to −17.2‰ in −Fe and −P/−Fe (−31%), reaching a stable value from Day 7 onwards in all treatments except −Fe where it decreased up to the last day (Figure 3). By comparing the different treatments, no significant differences (*p* = 0.543) were highlighted at root level. On the contrary, at shoot level, −Fe and −P/−Fe had significantly higher δ^13^C (+5%) as compared to the control and −P, in the last 5 days (Figure 3, Table S3).

#### 3.3.4 Tomato

The two‐way ANOVA highlighted that tomato δ^13^C was significantly altered by time (*p* < 0.001) and treatments (*p* < 0.001). In addition, the interaction between the two factors in both plant tissues was found to be highly significant (*p* < 0.001) (Table 1).

During the first 3 days of the time course, tomato's δ^13^C significantly decreased in both root and shoot, before reaching a constant level. Between Days 0 and 14, in roots, the δ^13^C decreased from −31.5‰ to −37.5‰ in the control (−19%), from −32.5‰ to −35.5‰ in −P (−9%), from −32.5‰ to −38.0‰ in −Fe (−17%), and from −32.5‰ to −36.0‰ in −P/−Fe (−11%). For shoots, instead the δ^13^C decreased from −34.0‰ to −39.0‰ in the control (−15%), from −35.5‰ to −39.0‰ in −Fe (−10%), and from −35.5‰ to −36.5‰ in −P and −P/−Fe (−3%) (Figure 3).

Considering the interaction between treatment and time, the four treatments did not show any significant differences in the first 3 days of the time course in both shoots and roots. From Day 7 onwards, −P and −P/−Fe showed a significantly higher δ^13^C as compared to control and −Fe (+5%). However, while at shoot level the differences in δ^13^C between P and combined deficiency was not statistically significant, at root level, it was (−P vs. −P/−Fe + 3%). Indeed, −P/−Fe roots had significantly higher δ^13^C values than control and −Fe roots but significantly lower values than −P roots (Figure 3, Table S3).

## 4 DISCUSSION

Great strides have been made in the comprehension of δ^13^C at the plant level (Tcherkez et al., 2006, 2011). Nevertheless, although the literature describes a fair number of studies performed in silico, that is, quantum chemical calculations (Tcherkez & Farquhar, 2005), and in vitro analyses, that is, enzymatic assays, followed by analyses of specific products or reactants by isotope ratio mass spectrometer (IRMS) (O'Leary, 1980), in vivo research is still very limited (Dercon et al., 2006; Rombolà et al., 2005; Tiziani et al., 2020). We therefore analyzed the δ^13^C in response to P and Fe deficiencies, in four contrasting plant species and tried to understand if δ^13^C could be used as a monitoring and/or predictive tool for abiotic stresses.

Time significantly affected the δ^13^C of all plant species and tissues tested (Figure 3). In all four plant species, δ^13^C decreased significantly between germination and the 14th day of growth (7 days in full NS and 7 days in treatment‐specific NS), in both roots and shoots (Figures 3 and S3). Even though the average decrease differed between plant species, the consistency of these results suggests that there may be a common biological mechanism determining this drop in δ^13^C during plant development. This phenomenon might be explained by a biomass dilution effect after seed germination. In fact, the measured δ^13^C of C3 and C4 seeds was −27.0‰ and −12.0‰, respectively (Table S4), as found also in previous studies (Kaler et al., 2018; Ludlow et al., 1976). Hence, the newly developed autotrophic plant tissues have a higher Δ, and consequently lower δ^13^C (Cernusak et al., 2009), thus tending to reduce the overall plant δ^13^C proportionally to the biomass produced. Even though this might be true for the first days of seedling development, it is unlikely to be still the main driving factor in two‐week old plantlets. This is especially true in the case of tomato, where the seed size is around 1:1000 of the biomass of two‐week old plantlets (3 ± 1 mg vs. 3000 ± 1000 mg, Table S4 and Figure S2). Therefore, even though the δ^13^C is higher in seeds, the huge dilution effect caused by plant growth will entail that the overall plant δ^13^C levels out in just a couple of days. However, in the first days of plant growth, the RuBisCO activity (Δ = 29.0‰–30.0‰) is reduced as compared to the PEPC activity (Δ = 2.2‰–3.0‰). Thus, the overall plant Δ determining the δ^13^C of newly developed plant tissues will be lower, and consequently, plants discriminate less against ^13^C, as compared to mature plants (Rival et al., 1998; Triques et al., 1997). This slow increase of the initially low RuBisCO activity, raising ^13^C discrimination and lowering the plants δ^13^C during plant development, could explain the observed results (Figure 3).

**FIGURE 4:**
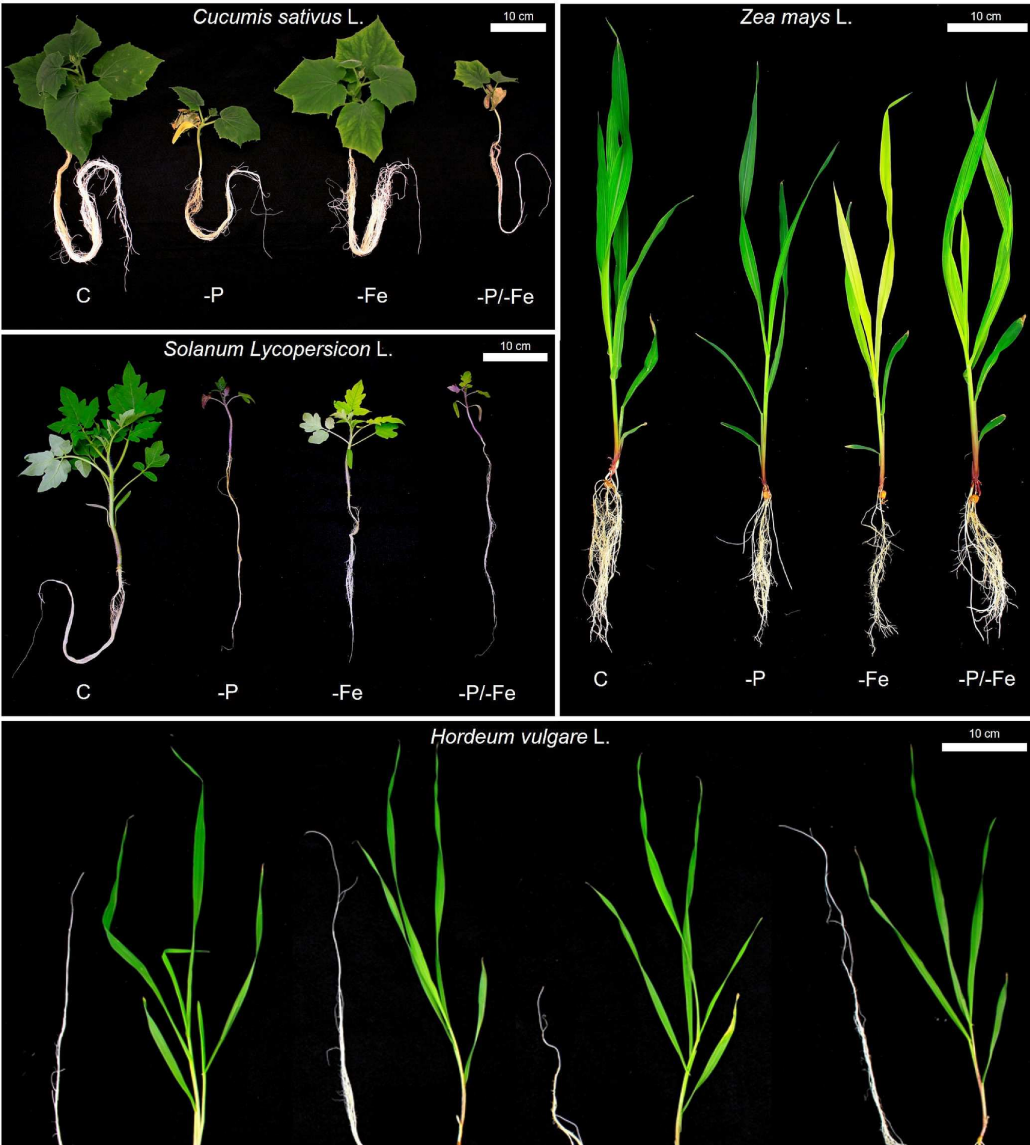
Pictures of representative plants for each species, barley ( *Hordeum vulgare* L.), cucumber ( *Cucumis sativus* L.), maize ( *Zea mays* L.), and tomato (*Solanum lycopersicon* L.), and treatment (C = control; −P = phosphorus deficiency; −Fe = iron deficiency; −P/−Fe = phosphorus and iron combined deficiencies). Scale bar on the top right of each picture = 10 cm.

In spite of a common behavior during the first days of plant development, towards the end of the experiment, treatment‐, species‐, and tissue‐specific responses in terms of Δ were observed (Figure 3) (Hypothesis i). Furthermore, the effect of nutrient deficiencies on the plants' δ^13^C varied over time in most plant species (Figure 3). Tomato and cucumber plants showed generally higher δ^13^C values in −P and −P/−Fe, that could be almost fully explained by the variation in stomatal conductance: a lower stomatal conductance determines a lower discrimination against ^13^C and therefore gives a higher δ^13^C (Figures 1 and 3). On the contrary, barley exhibited no treatment‐specific δ^13^C differences, despite clear phenotypic (Figure 4) and biomass differences (Table S4 and Figure S2). In maize, the treatment‐driven δ^13^C shifts, that is, higher δ^13^C in −Fe and −P/−Fe, were not attributable to changes in the stomatal conductance. However, the significantly lower photosynthetic rate (Figure 2) may be an indicator of a reduced RuBisCO activity. Decreased RuBisCO activity might enhance the partial contribution of other carbon fixation processes, for example, PEPC, characterized by a lower discrimination rate, and in turn, determine a higher δ^13^C. These results highlight the limitations of prediction models based only on photosynthetic Δ, that is, Farquhar's model as improved by Cernusak and Busch (Busch et al., 2020; Cernusak et al., 2013; Farquhar et al., 1989). Indeed, these models are unable to explain many significant differences in δ^13^C caused by abiotic stresses.

**FIGURE 5:**
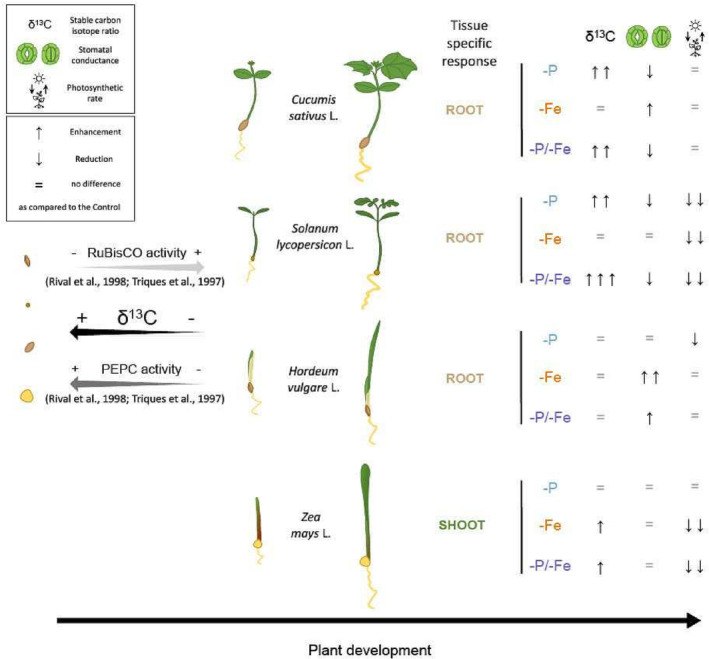
Summary of stable carbon isotope ratio (δ^13^C), stomatal conductance (gs), and photosynthetic rate (A) responses of the four analyzed plant species, barley ( *Hordeum vulgare* L.), cucumber ( *Cucumis sativus* L.), maize ( *Zea mays* L.), and tomato (*Solanum lycopersicon* L.), in relation to plant development and to different treatments (δ^13^C: ↓ = δ^13^C 0.5‰ lower, ↑ = δ^13^C 0.5‰ higher, ↑↑ = δ^13^C 2.0‰ higher, ↑↑↑ = δ^13^C 3.0‰ higher as compared to the control; gs: ↓ = gs 0.01 mol m^2^ s^−1^ lower, ↑ = gs 0.01 mol m^2^ s^−1^ higher, ↑↑ = gs 0.02 mol m^2^ s^−1^ higher as compared to the control; A: ↓ = A 0.5 μmol m^2^ s^−1^ lower, ↓↓ = A 1 μmol m^2^ s^−1^ lower as compared to the control). Ribulose‐1,5‐bisphosphate carboxylase‐oxygenase (RuBisCO) and phosphoenolpyruvate carboxylase (PEPC) activity represent the two main theorized drivers of δ^13^C variation during plant development. The tissue‐specific response highlight in which plant tissue the impact of treatments on the δ^13^C was more evident.

The δ^13^C response to −P/−Fe treatment resembled −P behavior in both roots and shoots along the whole time course in cucumber and tomato plants. In contrast, maize δ^13^C changes observed in −P/−Fe plants were similar to shifts in −Fe plants. These results do not only show that changes in plant δ^13^C are species specific but also that the perception of a combined, deficiency is also strictly species dependent. Therefore, interpreting δ^13^C values in natural conditions is considerably more complex than what might be expected (Figure 5).

Moreover, treatment‐driven differences in the δ^13^C of barley, cucumber, and tomato plants were found to be larger in root tissues as compared to shoots (Hypothesis i). However, in maize, the opposite trend was observed (larger differences in shoots as compared to roots) (Figure 3, Hypothesis ii). This species‐driven differential tissue specificity, might be ascribed to root PEPC activity (Feria et al., 2016). Indeed, at the root level, PEPC activity was proven to be enhanced under nutrient shortages (López‐Millán et al., 2009), especially in relation to the RuBisCO activity, which is generally decreased under abiotic stresses (Figure 2) (Warren, 2011; Warren & Adams, 2002). PEPC exhibits a lower Δ than RuBisCO (2.2‰–3.0‰ vs. 29.0‰–30.0‰), because it fixes the dissolved CO_2_ in the form of bicarbonate (HCO_3_^−^). Bicarbonate is then, in turn, ^13^C‐enriched due to fractionation (−9.0‰) coupled with the hydration equilibrium ([HCO_3_^−^]/[CO_2_]) (Mook et al., 1974), thus determining an overall ^13^C enrichment (−5.7‰) in the biomass produced starting from PEPC fixed carbon. Here, we emphasize, for the first time, the potential key role of roots in the plants Δ.

In accordance with the differences in species responses to nutrient stresses, it becomes evident that there is only limited variation explained by the photosynthesis type and by the nutrient acquisition strategy, except for the above‐mentioned root tissue specificity that seems to be connected to C3 photosynthesis (Hypotheses ii and iii). Hence, no generalization of the findings can be made in this regard (Figure 5). In contrast, the two dicots, tomato and cucumber, reacted similarly, allowing us to conclude that dicots may be more prone to alter their δ^13^C in response to nutrient stresses as compared to monocots.

Plant δ^13^C depends on metabolic fluxes and allocation patterns within metabolic pools (Kruger & Ratcliffe, 2009), on the Δ of all the carbon fixation, uptake, decarboxylation, and respiration processes (Ivlev, 2004; Tcherkez et al., 2011; Tcherkez & Farquhar, 2005), and not solely on the photosynthetic Δ. Hence, the theorized use of δ^13^C to foresee abiotic stresses needs caution, considering the current knowledge. Indeed, depending on the plant species and sometimes also to tissue type, the same nutrient deficiency may cause contrasting δ^13^C variations that might readily be misinterpreted. This could ultimately determine an overlooked nutrient shortage especially in the case of combined deficiencies (Figure 5). Moreover, plant physiology (Figures 1, 2, S1, and S2) and phenotype (Figure 4) clearly show nutrient deficient plants, highlighting the inability of the δ^13^C to foresee an abiotic stress.

In conclusion, we monitored, for the first time, the plant δ^13^C responses to three different nutrient stresses in four contrasting plant species. In all species and tissues, the δ^13^C significantly decreased during plant development, most probably due to a biomass dilution effect and to an initially low RuBisCO/PEPC activity ratio. The current study has highlighted both treatment‐ and species‐specific responses in terms of Δ, which were only partially correlated with the measured physiological parameters, suggesting the limits of prediction models based solely on photosynthetic Δ (Hypothesis i). Moreover, the photosynthesis mechanism (i.e., C3 versus C4) and/or cotyledon number explained only limited δ^13^C variation in response to nutrient stresses (Hypothesis ii and iii). This was substantiated not only by the different δ^13^C responses to nutrient deficiencies but also by the discrepancies in the combined deficiency and tissue specificity. The tissue‐specific responses suggest roots as playing an important role in the plant Δ. All these findings suggest that the use of the δ^13^C as a predictor of abiotic stresses is highly error prone unless a complete understanding of δ^13^C fractionation is available.

## AUTHOR CONTRIBUTIONS

Tanja Mimmo, Raphael Tiziani, and Stefano Cesco designed the research. Fabio Trevisan performed the research, and collected, analyzed, and interpreted the data. Fabio Trevisan, Tanja Mimmo, Raphael Tiziani, and Robert D. Hall wrote the article and critically evaluated both the manuscript and the results.

## CONFLICT OF INTEREST STATEMENT

The authors declare no conflicts of interest.

## Supporting information


**Figure S1** Transpiration Rate boxplots
**Figure S2** Fresh weights scatterplots
**Figure S3** Extended δ^13^C scatterplots
**Table S1** List of chosen plant species and some main characteristics
**Table S2** Nutrient solutions composition according to species
**Table S3** Statistical analyses performed on the δ^13^C results
**Table S4** Statistical analyses performed on the fresh weight

## Data Availability

The following information was supplied regarding data and code availability: the raw data, the version of the individual packages and scripts used to analyze the data and generate the figures of this study are available at GitHub: https://github.com/Fabio-Trevisan/13C-Experiment.git.
