# Supplementary material for: δ13C as a tool for iron and phosphorus deficiency prediction in crops
Source: Plant Direct. 2023 Mar 20;7(3):e487. doi: 10.1002/pld3.487 (PMC10027435; doi:10.1002/pld3.487)
Supplement: Supplementary file 1 — Figure S1 Transpiration Rate boxplots Figure S2 Fresh weights scatterplots Figure S3 Extended δ13C scatterplots Table S1 List of chosen plant species and some main characteristics Table S2 Nutrient solutions composition according to species Table S3 Statistical analyses performed on the δ13C results Table S4 Statistical analyses performed on the fresh weight [file PLD3-7-e487-s001.pdf]

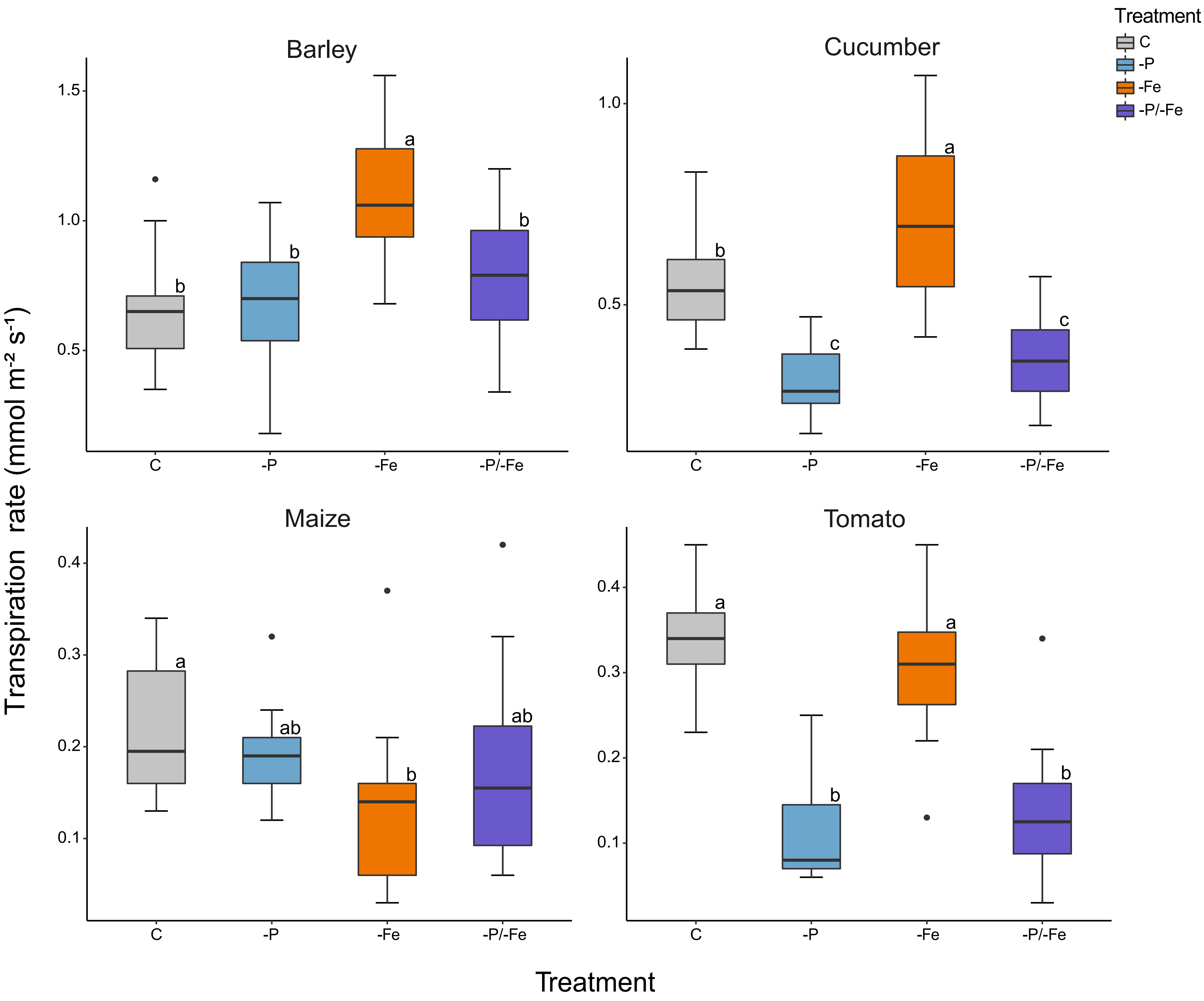


**Figure S1:** Transpiration Rate (mol $m^{-2}$ $s^{-1}$) of barley, cucumber, maize and tomato plants according to four different treatments: control (C), phosphorus deficiency (-P), iron deficiency (-Fe) and phosphorus and iron combined deficiencies (-P/-Fe) with n≥12. Letters next to the boxes indicate statistically significant differences (P<0.05) assessed by a one-way ANOVA with Tukey post hoc test.

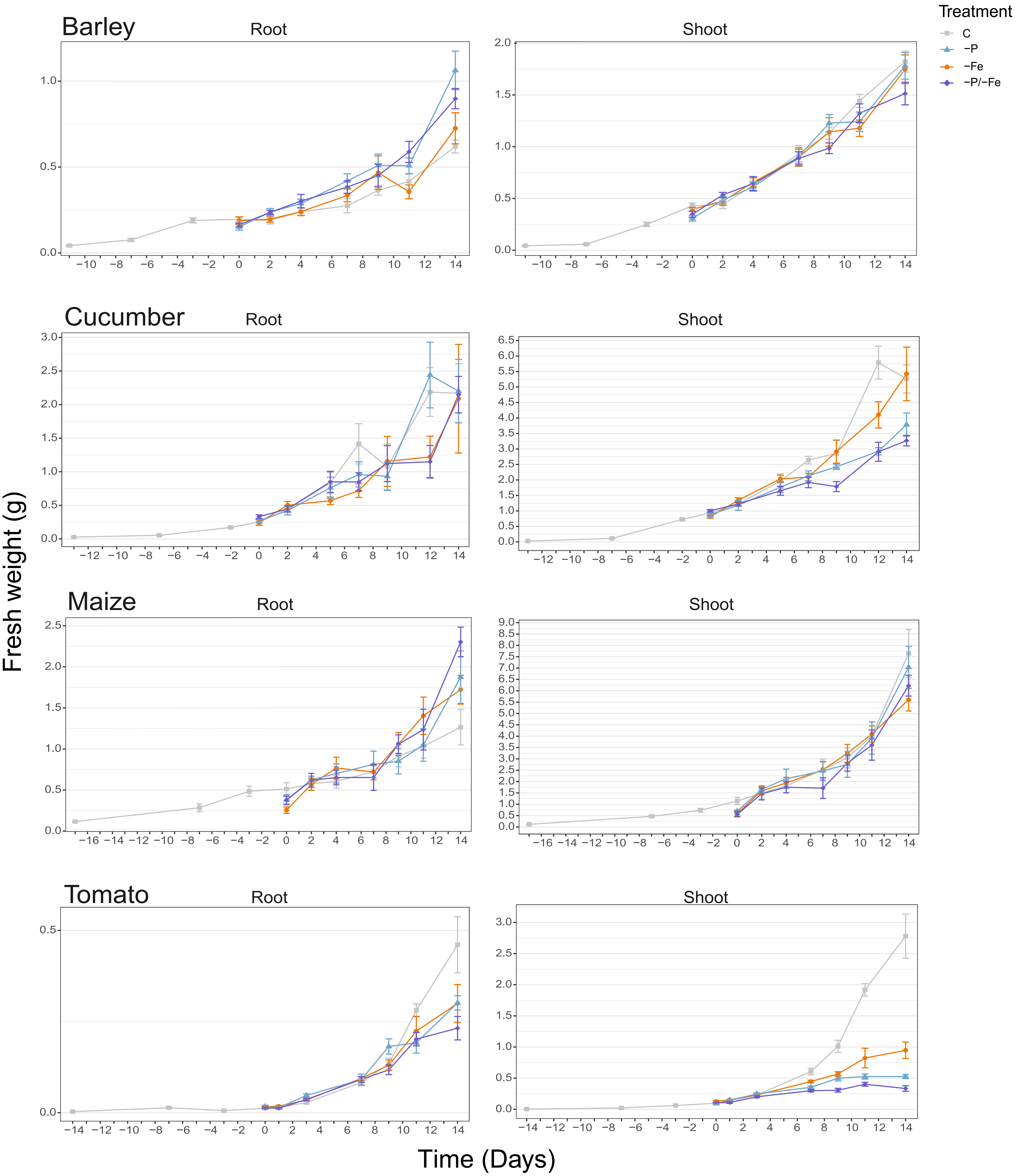


**Figure S2:** Fresh weights of barley, cucumber, maize and tomato plants grown under control conditions (C), phosphorus starvation (-P), iron starvation (-Fe) and combined phosphorus and iron starvation (-P/-Fe) plotted over time. The first point on the left of each graph describes the fresh weight of the seed represented as mean ± SE, n=3. Seeds were germinated in darkness for 4-10 days according to the plant species (barley 4 days, cucumber 6 days, maize 10 days, tomato 7 days). After germination, the young seedlings were exposed to the light and grown for 7 days in full nutrient solution (NS), *i.e.* second and third point starting from the left of each graph. Day 0 on the x axis indicates the day on which the plants were transferred to the treatment-specific NS. The fresh weight values of roots, left, and shoots, right, are represented as mean ± SE, n=7.

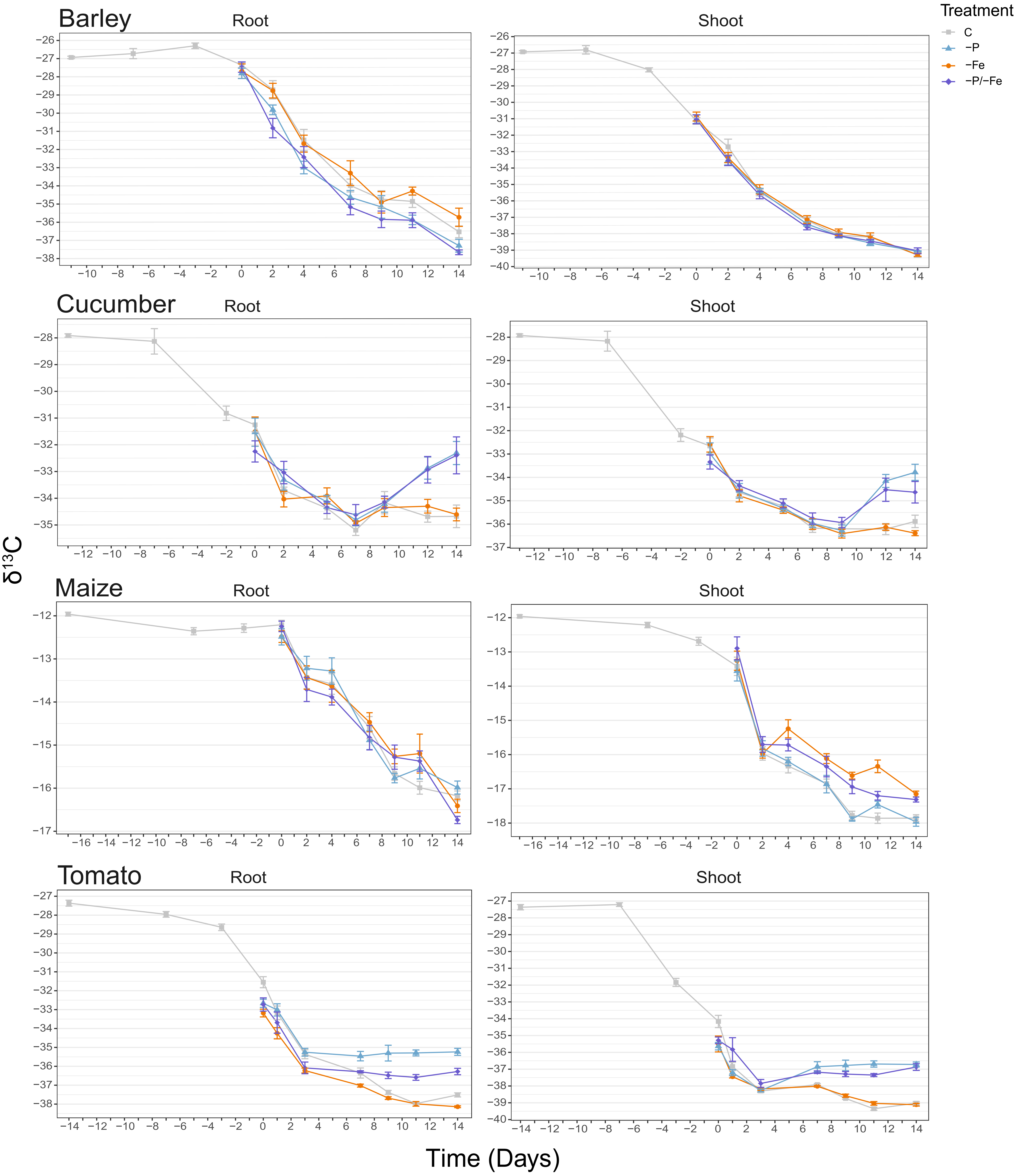


**Figure S3:** δ$^{13}$C values for barley, cucumber, maize and tomato plants grown under control conditions (C), phosphorus starvation (-P), iron starvation (-Fe) and combined phosphorus and iron starvation (-P/-Fe) plotted over time. The first point on the left of each graph describes the δ$^{13}$C values of the seed represented as mean ± SE, n=3. Seeds were germinated in darkness for 4-10 days according to the plant species (barley 4 days, cucumber 6 days, maize 10 days, tomato 7 days). After germination, the young seedlings were exposed to the light and grown for 7 days in full nutrient solution (NS), *i.e.* second and third point starting from the left of each graph. Day 0 on the x axis indicates the day on which the plants were transferred to the treatment-specific NS. The δ$^{13}$C values of roots, left, and shoots, right, are represented as mean ± SE, n=7.

**Table S1**: List of chosen plant species and some main characteristics. Namely: cultivar, photosynthetic pathway type (C3 or C4), Fe acquisition strategy (I or II), number of cotyledons (1 or 2) and botanical family.

| Plant species | Cultivar | Photosynthesis type | Fe acquisition | Cotyledon number and Family |
|---|---|---|---|---|
| *Solanum lycopersicon* L. (Tomato) | Marmande | C3 | Strategy I | Dicot (Solanaceae) |
| *Cucumis sativus* L. (Cucumber) | Chinese Long | C3 | Strategy I | Dicot (Cucurbitaceae) |
| *Hordeum vulgare* L. (Barley) | Solist | C3 | Strategy II | Monocot Poacee (Graminacea) |
| *Zea mays* L. (Maize) | F1 Hybrid P0423, Pioneer Hi-Bred Italia S.r.l | C4 | Strategy II | Monocot Poacee (Graminacea) |

**Table S2**: Nutrient solutions composition according to species. pH of each solution was measured and adjusted with either HCl or NaOH in case of markable differences.

| Plant species | $Ca(NO_3)_2$ (mmol $L^{-1}$) | $K_2SO_4$ (mmol $L^{-1}$) | $KH_2PO_4$ (mmol $L^{-1}$) | KCl (mmol $L^{-1}$) | $MgSO_4$ (mmol $L^{-1}$) | Micronutrients* (µmol $L^{-1}$) |
|---|---|---|---|---|---|---|
| *Solanum Lycopersicon* L. (Tomato) | 2 | 0.7 | 0.1 | 0.1 | 0.5 | 10 $H_3BO_3$<br>80 Fe(III)-EDTA<br>0.2 $CuSO_4$ |
| *Cucumis sativus* L. (Cucumber) | 2 | 0.7 | 0.1 | 0.1 | 0.5 | 10 $H_3BO_3$<br>80 Fe(III)-EDTA<br>0.2 $CuSO_4$ |
| *Hordeum vulgare* L. (Barley) | 2 | 0.7 | 0.1 | 0.1 | 0.5 | 1 $H_3BO_3$<br>100 Fe(III)-EDTA<br>0.2 $CuSO_4$ |
| *Zea mays* L. (Maize) | 2 | 0.7 | 0.1 | 0.1 | 0.5 | 1 $H_3BO_3$<br>100 Fe(III)-EDTA<br>0.5 $CuSO_4$ |

*(µmol $L^{-1}$): 0.5 $MnSO_4$, 0.5 $ZnSO_4$ and 0.01 $(NH_4)_6Mo_7O_{24}$

**Table S3:** Table summarizing the statistical analyses performed on the $\delta^{13}C$ results shown in the article main text (Figure 3). The table is divided in six sections: summary table for number of replicates (N), stable carbon isotope ratio value ($\delta^{13}C$), standard deviation (sd) and standard error (se), Two-Way Anova, One-Way Anova on treatments, Tukey.HSD multiple comparison on treatments, One-Way Anova on time and Tukey.HSD multiple comparison on time. BR= Barley Root; BS=Barley Shoot; CR= Cucumber Root; CS=Cucumber Shoot; MR= Maize Root; MS=Maize Shoot; TS=Tomato Shoot; TR= Tomato Root.

***Summary table***

| Treatment | Time | Species_Tissue | N | $\delta^{13}C$ | sd | se |
|---|---|---|---|---|---|---|
| C | 0 | Barley_Root | 7 | -27.37 | 0.38 | 0.14 |
| C | 0 | Barley_Shoot | 7 | -31.16 | 0.54 | 0.20 |
| C | 0 | Cucumber_Root | 7 | -31.26 | 0.70 | 0.26 |
| C | 0 | Cucumber_Shoot | 7 | -32.66 | 0.91 | 0.35 |
| C | 0 | Maize_Root | 7 | -12.21 | 0.28 | 0.10 |
| C | 0 | Maize_Shoot | 7 | -13.42 | 0.71 | 0.27 |
| C | 0 | Tomato_Root | 7 | -31.55 | 0.77 | 0.29 |
| C | 0 | Tomato_Shoot | 7 | -34.16 | 0.96 | 0.36 |
| C | 1 | Tomato_Root | 7 | -33.17 | 0.96 | 0.36 |
| C | 1 | Tomato_Shoot | 7 | -36.84 | 0.64 | 0.24 |
| C | 2 | Barley_Root | 7 | -28.74 | 1.33 | 0.50 |
| C | 2 | Barley_Shoot | 7 | -32.73 | 1.22 | 0.46 |
| C | 2 | Cucumber_Root | 7 | -33.72 | 0.32 | 0.12 |
| C | 2 | Cucumber_Shoot | 7 | -34.65 | 0.47 | 0.18 |
| C | 2 | Maize_Root | 7 | -13.43 | 0.68 | 0.26 |
| C | 2 | Maize_Shoot | 7 | -15.97 | 0.51 | 0.19 |
| C | 3 | Tomato_Root | 7 | -35.36 | 0.63 | 0.24 |
| C | 3 | Tomato_Shoot | 7 | -38.30 | 0.25 | 0.09 |
| C | 4 | Barley_Root | 7 | -31.50 | 1.55 | 0.59 |
| C | 4 | Barley_Shoot | 7 | -35.52 | 0.53 | 0.20 |
| C | 4 | Maize_Root | 7 | -13.58 | 0.63 | 0.24 |
| C | 4 | Maize_Shoot | 7 | -16.34 | 0.53 | 0.20 |
| C | 5 | Cucumber_Root | 7 | -34.38 | 1.08 | 0.41 |
| C | 5 | Cucumber_Shoot | 7 | -35.23 | 0.78 | 0.29 |
| C | 7 | Barley_Root | 7 | -34.00 | 0.94 | 0.35 |
| C | 7 | Barley_Shoot | 7 | -37.21 | 0.48 | 0.18 |
| C | 7 | Cucumber_Root | 7 | -35.21 | 0.52 | 0.20 |
| C | 7 | Cucumber_Shoot | 7 | -36.17 | 0.48 | 0.18 |
| C | 7 | Maize_Root | 7 | -14.60 | 0.68 | 0.26 |
| C | 7 | Maize_Shoot | 7 | -16.85 | 0.35 | 0.13 |
| C | 7 | Tomato_Root | 7 | -36.36 | 0.71 | 0.27 |
| C | 7 | Tomato_Shoot | 7 | -37.91 | 0.15 | 0.06 |
| C | 9 | Barley_Root | 7 | -34.75 | 1.22 | 0.46 |
| C | 9 | Barley_Shoot | 7 | -38.11 | 0.48 | 0.18 |
| C | 9 | Cucumber_Root | 7 | -34.18 | 1.10 | 0.42 |
| C | 9 | Cucumber_Shoot | 7 | -36.20 | 0.63 | 0.24 |
| C | 9 | Maize_Root | 7 | -15.66 | 0.41 | 0.16 |

| | | | | | | |
|---|---|---|---|---|---|---|
| C | 9 | Maize_Shoot | 7 | -17.78 | 0.32 | 0.12 |
| C | 9 | Tomato_Root | 7 | -37.38 | 0.28 | 0.11 |
| C | 9 | Tomato_Shoot | 7 | -38.74 | 0.14 | 0.05 |
| C | 11 | Barley_Root | 7 | -34.87 | 0.89 | 0.33 |
| C | 11 | Barley_Shoot | 7 | -38.24 | 0.31 | 0.12 |
| C | 11 | Maize_Root | 7 | -15.99 | 0.39 | 0.15 |
| C | 11 | Maize_Shoot | 7 | -17.86 | 0.40 | 0.15 |
| C | 11 | Tomato_Root | 7 | -37.97 | 0.09 | 0.03 |
| C | 11 | Tomato_Shoot | 7 | -39.35 | 0.23 | 0.09 |
| C | 12 | Cucumber_Root | 7 | -34.70 | 0.55 | 0.21 |
| C | 12 | Cucumber_Shoot | 7 | -36.21 | 0.63 | 0.24 |
| C | 14 | Barley_Root | 7 | -36.55 | 0.85 | 0.32 |
| C | 14 | Barley_Shoot | 7 | -39.33 | 0.36 | 0.14 |
| C | 14 | Cucumber_Root | 7 | -34.69 | 1.12 | 0.42 |
| C | 14 | Cucumber_Shoot | 7 | -35.88 | 0.70 | 0.27 |
| C | 14 | Maize_Root | 7 | -16.18 | 0.30 | 0.11 |
| C | 14 | Maize_Shoot | 7 | -17.86 | 0.26 | 0.10 |
| C | 14 | Tomato_Root | 7 | -37.51 | 0.29 | 0.11 |
| C | 14 | Tomato_Shoot | 7 | -39.02 | 0.26 | 0.10 |
| Fe | 0 | Barley_Root | 7 | -27.71 | 1.06 | 0.40 |
| Fe | 0 | Barley_Shoot | 7 | -30.90 | 0.72 | 0.27 |
| Fe | 0 | Cucumber_Root | 7 | -31.52 | 1.44 | 0.55 |
| Fe | 0 | Cucumber_Shoot | 7 | -32.59 | 0.88 | 0.33 |
| Fe | 0 | Maize_Root | 7 | -12.48 | 0.36 | 0.14 |
| Fe | 0 | Maize_Shoot | 7 | -13.26 | 0.74 | 0.28 |
| Fe | 0 | Tomato_Root | 7 | -33.18 | 0.54 | 0.21 |
| Fe | 0 | Tomato_Shoot | 7 | -35.50 | 1.25 | 0.47 |
| Fe | 1 | Tomato_Root | 7 | -34.25 | 0.78 | 0.30 |
| Fe | 1 | Tomato_Shoot | 7 | -37.45 | 0.20 | 0.07 |
| Fe | 2 | Barley_Root | 7 | -28.78 | 1.07 | 0.40 |
| Fe | 2 | Barley_Shoot | 7 | -33.40 | 0.82 | 0.31 |
| Fe | 2 | Cucumber_Root | 7 | -34.04 | 0.77 | 0.29 |
| Fe | 2 | Cucumber_Shoot | 7 | -34.79 | 0.69 | 0.26 |
| Fe | 2 | Maize_Root | 7 | -13.43 | 0.72 | 0.27 |
| Fe | 2 | Maize_Shoot | 7 | -15.97 | 0.35 | 0.13 |
| Fe | 3 | Tomato_Root | 7 | -36.22 | 0.33 | 0.13 |
| Fe | 3 | Tomato_Shoot | 7 | -38.17 | 0.23 | 0.09 |
| Fe | 4 | Barley_Root | 7 | -31.70 | 1.23 | 0.47 |
| Fe | 4 | Barley_Shoot | 7 | -35.33 | 0.74 | 0.28 |
| Fe | 4 | Maize_Root | 7 | -13.64 | 0.98 | 0.37 |
| Fe | 4 | Maize_Shoot | 7 | -15.25 | 0.70 | 0.26 |
| Fe | 5 | Cucumber_Root | 7 | -33.92 | 0.80 | 0.30 |
| Fe | 5 | Cucumber_Shoot | 7 | -35.40 | 0.36 | 0.14 |
| Fe | 7 | Barley_Root | 7 | -33.32 | 1.79 | 0.68 |
| Fe | 7 | Barley_Shoot | 7 | -37.16 | 0.61 | 0.23 |
| Fe | 7 | Cucumber_Root | 7 | -34.95 | 0.23 | 0.09 |
| Fe | 7 | Cucumber_Shoot | 7 | -36.00 | 0.60 | 0.23 |
| Fe | 7 | Maize_Root | 7 | -14.47 | 0.59 | 0.22 |
| Fe | 7 | Maize_Shoot | 7 | -16.12 | 0.38 | 0.14 |
| Fe | 7 | Tomato_Root | 7 | -37.02 | 0.21 | 0.08 |

| | | | | | | |
|---|---|---|---|---|---|---|
| Fe | 7 | Tomato_Shoot | 7 | -38.02 | 0.13 | 0.05 |
| Fe | 9 | Barley_Root | 7 | -34.93 | 1.56 | 0.59 |
| Fe | 9 | Barley_Shoot | 7 | -37.94 | 0.52 | 0.20 |
| Fe | 9 | Cucumber_Root | 7 | -34.36 | 0.88 | 0.33 |
| Fe | 9 | Cucumber_Shoot | 7 | -36.41 | 0.50 | 0.19 |
| Fe | 9 | Maize_Root | 7 | -15.26 | 0.45 | 0.17 |
| Fe | 9 | Maize_Shoot | 7 | -16.61 | 0.26 | 0.10 |
| Fe | 9 | Tomato_Root | 7 | -37.68 | 0.19 | 0.07 |
| Fe | 9 | Tomato_Shoot | 7 | -38.58 | 0.26 | 0.10 |
| Fe | 11 | Barley_Root | 7 | -34.31 | 0.57 | 0.22 |
| Fe | 11 | Barley_Shoot | 7 | -38.21 | 0.60 | 0.23 |
| Fe | 11 | Maize_Root | 7 | -15.20 | 1.20 | 0.45 |
| Fe | 11 | Maize_Shoot | 7 | -16.34 | 0.49 | 0.18 |
| Fe | 11 | Tomato_Root | 7 | -37.99 | 0.32 | 0.12 |
| Fe | 11 | Tomato_Shoot | 7 | -39.03 | 0.29 | 0.11 |
| Fe | 12 | Cucumber_Root | 7 | -34.31 | 0.68 | 0.26 |
| Fe | 12 | Cucumber_Shoot | 7 | -36.13 | 0.36 | 0.14 |
| Fe | 14 | Barley_Root | 7 | -35.75 | 1.33 | 0.50 |
| Fe | 14 | Barley_Shoot | 7 | -39.32 | 0.24 | 0.09 |
| Fe | 14 | Cucumber_Root | 7 | -34.62 | 0.63 | 0.24 |
| Fe | 14 | Cucumber_Shoot | 7 | -36.39 | 0.28 | 0.11 |
| Fe | 14 | Maize_Root | 7 | -16.41 | 0.41 | 0.16 |
| Fe | 14 | Maize_Shoot | 7 | -17.15 | 0.22 | 0.08 |
| Fe | 14 | Tomato_Root | 7 | -38.14 | 0.13 | 0.05 |
| Fe | 14 | Tomato_Shoot | 7 | -39.11 | 0.21 | 0.08 |
| P | 0 | Barley_Root | 7 | -27.83 | 0.75 | 0.28 |
| P | 0 | Barley_Shoot | 7 | -31.05 | 0.71 | 0.27 |
| P | 0 | Cucumber_Root | 7 | -31.54 | 1.39 | 0.53 |
| P | 0 | Cucumber_Shoot | 7 | -32.98 | 1.21 | 0.46 |
| P | 0 | Maize_Root | 7 | -12.48 | 0.51 | 0.19 |
| P | 0 | Maize_Shoot | 7 | -13.56 | 0.77 | 0.29 |
| P | 0 | Tomato_Root | 7 | -32.67 | 0.58 | 0.22 |
| P | 0 | Tomato_Shoot | 7 | -35.63 | 0.59 | 0.22 |
| P | 1 | Tomato_Root | 7 | -33.01 | 0.85 | 0.32 |
| P | 1 | Tomato_Shoot | 7 | -37.21 | 0.47 | 0.18 |
| P | 2 | Barley_Root | 7 | -29.84 | 0.70 | 0.27 |
| P | 2 | Barley_Shoot | 7 | -33.56 | 0.74 | 0.28 |
| P | 2 | Cucumber_Root | 7 | -33.32 | 1.02 | 0.39 |
| P | 2 | Cucumber_Shoot | 7 | -34.58 | 0.83 | 0.31 |
| P | 2 | Maize_Root | 7 | -13.22 | 0.73 | 0.28 |
| P | 2 | Maize_Shoot | 7 | -15.82 | 0.60 | 0.23 |
| P | 3 | Tomato_Root | 7 | -35.25 | 0.54 | 0.20 |
| P | 3 | Tomato_Shoot | 7 | -38.27 | 0.21 | 0.08 |
| P | 4 | Barley_Root | 7 | -33.01 | 0.91 | 0.35 |
| P | 4 | Barley_Shoot | 7 | -35.41 | 0.38 | 0.14 |
| P | 4 | Maize_Root | 7 | -13.28 | 0.81 | 0.31 |
| P | 4 | Maize_Shoot | 7 | -16.20 | 0.32 | 0.12 |
| P | 5 | Cucumber_Root | 7 | -34.15 | 0.65 | 0.24 |
| P | 5 | Cucumber_Shoot | 7 | -35.32 | 0.36 | 0.14 |
| P | 7 | Barley_Root | 7 | -34.65 | 1.00 | 0.38 |

| | | | | | | |
|---|---|---|---|---|---|---|
| P | 7 | Barley_Shoot | 7 | -37.44 | 0.34 | 0.13 |
| P | 7 | Cucumber_Root | 7 | -34.82 | 0.59 | 0.22 |
| P | 7 | Cucumber_Shoot | 7 | -35.97 | 0.44 | 0.17 |
| P | 7 | Maize_Root | 7 | -14.89 | 0.58 | 0.22 |
| P | 7 | Maize_Shoot | 7 | -16.86 | 0.68 | 0.26 |
| P | 7 | Tomato_Root | 7 | -35.46 | 0.66 | 0.25 |
| P | 7 | Tomato_Shoot | 7 | -36.85 | 0.78 | 0.30 |
| P | 9 | Barley_Root | 7 | -35.18 | 1.64 | 0.62 |
| P | 9 | Barley_Shoot | 7 | -38.16 | 0.34 | 0.13 |
| P | 9 | Cucumber_Root | 7 | -34.23 | 0.80 | 0.30 |
| P | 9 | Cucumber_Shoot | 7 | -36.28 | 0.63 | 0.24 |
| P | 9 | Maize_Root | 7 | -15.77 | 0.29 | 0.11 |
| P | 9 | Maize_Shoot | 7 | -17.88 | 0.16 | 0.06 |
| P | 9 | Tomato_Root | 7 | -35.30 | 1.10 | 0.42 |
| P | 9 | Tomato_Shoot | 7 | -36.78 | 0.83 | 0.31 |
| P | 11 | Barley_Root | 7 | -35.89 | 0.69 | 0.26 |
| P | 11 | Barley_Shoot | 7 | -38.59 | 0.41 | 0.16 |
| P | 11 | Maize_Root | 7 | -15.54 | 0.65 | 0.25 |
| P | 11 | Maize_Shoot | 7 | -17.46 | 0.26 | 0.10 |
| P | 11 | Tomato_Root | 7 | -35.29 | 0.42 | 0.16 |
| P | 11 | Tomato_Shoot | 7 | -36.69 | 0.49 | 0.19 |
| P | 12 | Cucumber_Root | 7 | -32.88 | 1.10 | 0.41 |
| P | 12 | Cucumber_Shoot | 7 | -34.15 | 0.74 | 0.28 |
| P | 14 | Barley_Root | 7 | -37.31 | 0.94 | 0.35 |
| P | 14 | Barley_Shoot | 7 | -39.10 | 0.09 | 0.03 |
| P | 14 | Cucumber_Root | 7 | -32.32 | 1.14 | 0.43 |
| P | 14 | Cucumber_Shoot | 7 | -33.78 | 0.92 | 0.35 |
| P | 14 | Maize_Root | 7 | -15.99 | 0.40 | 0.15 |
| P | 14 | Maize_Shoot | 7 | -17.96 | 0.35 | 0.13 |
| P | 14 | Tomato_Root | 7 | -35.24 | 0.49 | 0.19 |
| P | 14 | Tomato_Shoot | 7 | -36.72 | 0.39 | 0.15 |
| P/Fe | 0 | Barley_Root | 7 | -27.49 | 0.75 | 0.28 |
| P/Fe | 0 | Barley_Shoot | 7 | -31.06 | 0.72 | 0.27 |
| P/Fe | 0 | Cucumber_Root | 7 | -32.26 | 1.05 | 0.40 |
| P/Fe | 0 | Cucumber_Shoot | 7 | -33.34 | 0.80 | 0.30 |
| P/Fe | 0 | Maize_Root | 7 | -12.25 | 0.32 | 0.12 |
| P/Fe | 0 | Maize_Shoot | 7 | -12.89 | 0.88 | 0.33 |
| P/Fe | 0 | Tomato_Root | 7 | -32.73 | 0.93 | 0.35 |
| P/Fe | 0 | Tomato_Shoot | 7 | -35.28 | 0.52 | 0.20 |
| P/Fe | 1 | Tomato_Root | 7 | -33.69 | 1.48 | 0.56 |
| P/Fe | 1 | Tomato_Shoot | 7 | -35.83 | 1.85 | 0.70 |
| P/Fe | 2 | Barley_Root | 7 | -30.84 | 1.41 | 0.53 |
| P/Fe | 2 | Barley_Shoot | 7 | -33.58 | 0.81 | 0.30 |
| P/Fe | 2 | Cucumber_Root | 7 | -33.05 | 1.11 | 0.42 |
| P/Fe | 2 | Cucumber_Shoot | 7 | -34.36 | 0.58 | 0.22 |
| P/Fe | 2 | Maize_Root | 7 | -13.71 | 0.74 | 0.28 |
| P/Fe | 2 | Maize_Shoot | 7 | -15.70 | 0.61 | 0.23 |
| P/Fe | 3 | Tomato_Root | 7 | -36.08 | 0.82 | 0.31 |
| P/Fe | 3 | Tomato_Shoot | 7 | -37.85 | 0.62 | 0.24 |
| P/Fe | 4 | Barley_Root | 7 | -32.44 | 1.53 | 0.58 |

| | | | | | | |
|---|---|---|---|---|---|---|
| P/Fe | 4 | Barley_Shoot | 7 | -35.67 | 0.60 | 0.23 |
| P/Fe | 4 | Maize_Root | 7 | -13.89 | 0.49 | 0.18 |
| P/Fe | 4 | Maize_Shoot | 7 | -15.72 | 0.45 | 0.17 |
| P/Fe | 5 | Cucumber_Root | 7 | -34.36 | 0.58 | 0.22 |
| P/Fe | 5 | Cucumber_Shoot | 7 | -35.11 | 0.51 | 0.19 |
| P/Fe | 7 | Barley_Root | 7 | -35.19 | 1.12 | 0.42 |
| P/Fe | 7 | Barley_Shoot | 7 | -37.63 | 0.43 | 0.16 |
| P/Fe | 7 | Cucumber_Root | 7 | -34.63 | 1.02 | 0.38 |
| P/Fe | 7 | Cucumber_Shoot | 7 | -35.76 | 0.63 | 0.24 |
| P/Fe | 7 | Maize_Root | 7 | -14.83 | 0.75 | 0.28 |
| P/Fe | 7 | Maize_Shoot | 7 | -16.35 | 0.80 | 0.30 |
| P/Fe | 7 | Tomato_Root | 7 | -36.29 | 0.15 | 0.06 |
| P/Fe | 7 | Tomato_Shoot | 7 | -37.18 | 0.20 | 0.08 |
| P/Fe | 9 | Barley_Root | 7 | -35.86 | 1.20 | 0.45 |
| P/Fe | 9 | Barley_Shoot | 7 | -38.15 | 0.23 | 0.09 |
| P/Fe | 9 | Cucumber_Root | 7 | -34.16 | 0.58 | 0.22 |
| P/Fe | 9 | Cucumber_Shoot | 7 | -35.94 | 0.59 | 0.22 |
| P/Fe | 9 | Maize_Root | 7 | -15.28 | 0.74 | 0.28 |
| P/Fe | 9 | Maize_Shoot | 7 | -16.94 | 0.53 | 0.20 |
| P/Fe | 9 | Tomato_Root | 7 | -36.48 | 0.45 | 0.17 |
| P/Fe | 9 | Tomato_Shoot | 7 | -37.29 | 0.40 | 0.15 |
| P/Fe | 11 | Barley_Root | 7 | -35.91 | 1.08 | 0.41 |
| P/Fe | 11 | Barley_Shoot | 7 | -38.47 | 0.24 | 0.09 |
| P/Fe | 11 | Maize_Root | 7 | -15.37 | 0.62 | 0.24 |
| P/Fe | 11 | Maize_Shoot | 7 | -17.20 | 0.33 | 0.13 |
| P/Fe | 11 | Tomato_Root | 7 | -36.59 | 0.40 | 0.15 |
| P/Fe | 11 | Tomato_Shoot | 7 | -37.35 | 0.24 | 0.09 |
| P/Fe | 12 | Cucumber_Root | 7 | -32.95 | 1.31 | 0.50 |
| P/Fe | 12 | Cucumber_Shoot | 7 | -34.53 | 1.31 | 0.50 |
| P/Fe | 14 | Barley_Root | 7 | -37.68 | 0.35 | 0.13 |
| P/Fe | 14 | Barley_Shoot | 7 | -39.07 | 0.47 | 0.18 |
| P/Fe | 14 | Cucumber_Root | 7 | -32.41 | 1.83 | 0.69 |
| P/Fe | 14 | Cucumber_Shoot | 7 | -34.64 | 1.22 | 0.46 |
| P/Fe | 14 | Maize_Root | 7 | -16.74 | 0.22 | 0.08 |
| P/Fe | 14 | Maize_Shoot | 7 | -17.31 | 0.19 | 0.07 |
| P/Fe | 14 | Tomato_Root | 7 | -36.28 | 0.45 | 0.17 |
| P/Fe | 14 | Tomato_Shoot | 7 | -36.87 | 0.53 | 0.20 |

**Two-Way ANOVA**

***$Barley_Root***

| | | | | | | |
|---|---|---|---|---|---|---|
| Analysis | of | Variance | Table | | | |
| | | | | | | |
| Response: | Value | | | | | |
| | Df | Sum Sq | Mean Sq | F value | Pr(>F) | |
| Treatment | 3 | 57.36 | 19.12 | 15.0482 | 1.01E-08 | *** |
| Time | 6 | 1898.83 | 316.47 | 249.0839 | <2.2E-16 | *** |
| Treatment:Time | 18 | 22.04 | 1.22 | 0.9635 | 0.5041 | |
| Residuals | 168 | 213.45 | 1.27 | | | |
| --- | | | | | | |
| Signif. | codes: | 0 | '***' | 0.001 | '**' | 0.01 '*' |
| | | 0.05 | '.' | 0.1 | '' | 1 |

***$Barley_Shoot***

| | | | | | | |
|---|---|---|---|---|---|---|
| Analysis | of | Variance | Table | | | |
| | | | | | | |
| Response: | Value | | | | | |
| | Df | Sum Sq | Mean Sq | F value | Pr(>F) | |
| Treatment | 3 | 1.49 | 0.497 | 1.5154 | 0.2124 | |
| Time | 6 | 1513.14 | 252.19 | 768.394 | <2.2E-16 | *** |
| Treatment:Time | 18 | 4.87 | 0.271 | 0.8243 | 0.6699 | |
| Residuals | 168 | 55.14 | 0.328 | | | |
| --- | | | | | | |
| Signif. | codes: | 0 | '***' | 0.001 | '**' | 0.01 '*' |
| | | 0.05 | '.' | 0.1 | '' | 1 |

***$Cucumber_Root***

| | | | | | | |
|---|---|---|---|---|---|---|
| Analysis | of | Variance | Table | | | |
| | | | | | | |
| Response: | Value | | | | | |
| | Df | Sum Sq | Mean Sq | F value | Pr(>F) | |
| Treatment | 3 | 19.499 | 6.4996 | 7.0611 | 1.69E-04 | *** |
| Time | 6 | 175.479 | 29.2466 | 31.773 | <2.2E-16 | *** |
| Treatment:Time | 18 | 45.736 | 2.5409 | 2.7604 | 3.47E-04 | *** |
| Residuals | 168 | 154.641 | 0.9205 | | | |
| --- | | | | | | |
| Signif. | codes: | 0 | '***' | 0.001 | '**' | 0.01 '*' |
| | | 0.05 | '.' | 0.1 | '' | 1 |

***$Cucumber_Shoot***

| | | | | | | |
|---|---|---|---|---|---|---|
| Analysis | of | Variance | Table | | | |
| | | | | | | |
| Response: | Value | | | | | |
| | Df | Sum Sq | Mean Sq | F value | Pr(>F) | |
| Treatment | 3 | 16.311 | 5.437 | 10.2622 | 3.06E-06 | *** |
| Time | 6 | 200.514 | 33.419 | 63.0772 | <2.2E-16 | *** |

| | | | | | | | | |
|---|---|---|---|---|---|---|---|---|
| Treatment:Time | 18 | 42.011 | 2.334 | 4.4052 | 1.02E-07 | *** | | |
| Residuals | 168 | 89.008 | 0.53 | | | | | |
| --- | | | | | | | | |
| Signif. | codes: | 0 | ‘***’ | 0.001 | ‘**’ | | 0.01 | ‘*’ |
| | | 0.05 | ‘.’ | 0.1 | ‘’ | | 1 | |

***$Maize_Root***

Analysis of Variance Table

Response: Value

| | Df | Sum Sq | Mean Sq | F value | Pr(>F) | | | |
|---|---|---|---|---|---|---|---|---|
| Treatment | 3 | 0.8 | 0.268 | 0.7176 | 0.5427 | | | |
| Time | 6 | 334.36 | 55.726 | 149.1721 | <2.2E-16 | *** | | |
| Treatment:Time | 18 | 8.65 | 0.481 | 1.2869 | 0.2019 | | | |
| Residuals | 168 | 62.76 | 0.374 | | | | | |
| --- | | | | | | | | |
| Signif. | codes: | 0 | ‘***’ | 0.001 | ‘**’ | | 0.01 | ‘*’ |
| | | 0.05 | ‘.’ | 0.1 | ‘’ | | 1 | |

***$Maize_Shoot***

Analysis of Variance Table

Response: Value

| | Df | Sum Sq | Mean Sq | F value | Pr(>F) | | | |
|---|---|---|---|---|---|---|---|---|
| Treatment | 3 | 21.28 | 7.095 | 27.1838 | 2.21E-14 | *** | | |
| Time | 6 | 363.1 | 60.516 | 231.8745 | <2.2E-16 | *** | | |
| Treatment:Time | 18 | 9.03 | 0.502 | 1.9216 | 0.0171 | * | | |
| Residuals | 168 | 43.85 | 0.261 | | | | | |
| --- | | | | | | | | |
| Signif. | codes: | 0 | ‘***’ | 0.001 | ‘**’ | | 0.01 | ‘*’ |
| | | 0.05 | ‘.’ | 0.1 | ‘’ | | 1 | |

***$Tomato_Root***

Analysis of Variance Table

Response: Value

| | Df | Sum Sq | Mean Sq | F value | Pr(>F) | | | |
|---|---|---|---|---|---|---|---|---|
| Treatment | 3 | 75.99 | 25.331 | 61.4799 | <2.2E-16 | *** | | |
| Time | 6 | 521.25 | 86.876 | 210.8502 | <2.2E-16 | *** | | |
| Treatment:Time | 18 | 48.61 | 2.7 | 6.5539 | 3.54E-12 | *** | | |
| Residuals | 168 | 69.22 | 0.412 | | | | | |
| --- | | | | | | | | |
| Signif. | codes: | 0 | ‘***’ | 0.001 | ‘**’ | | 0.01 | ‘*’ |
| | | 0.05 | ‘.’ | 0.1 | ‘’ | | 1 | |

***$Tomato_Shoot***

Analysis of Variance Table

Response: Value

| | Df | Sum Sq | Mean Sq | F value | Pr(>F) | | |
|---|---|---|---|---|---|---|---|
| Treatment | 3 | 53.155 | 17.718 | 47.8028 | <2.2E-16 | *** | |
| Time | 6 | 194.537 | 32.423 | 87.474 | <2.2E-16 | *** | |
| Treatment:Time | 18 | 65.361 | 3.631 | 9.7966 | <2.2E-16 | *** | |
| Residuals | 168 | 62.27 | 0.371 | | | | |
| --- | | | | | | | |
| Signif. | codes: | 0 | ‘***’ | 0.001 | ‘**’ | 0.01 | ‘*’ |
| | | 0.05 | ‘.’ | 0.1 | ‘’ | 1 | |

**One-Way ANOVA on treatments**

***$Barley_Root***

$Barley_Root$`0`

Analysis of Variance Table

Response: Value

| | Df | Sum Sq | Mean Sq | F value | Pr(>F) | |
|---|---|---|---|---|---|---|
| Treatment | 3 | 0.9258 | 0.3086 | 0.5142 | 0.6764 | |
| Residuals | 24 | 14.4026 | 0.60011 | | | |

$Barley_Root$`2`

Analysis of Variance Table

Response: Value

| | Df | Sum Sq | Mean Sq | F value | Pr(>F) | |
|---|---|---|---|---|---|---|
| Treatment | 3 | 21.034 | 7.0113 | 5.187 | 0.006635 | ** |
| Residuals | 24 | 32.441 | 1.3517 | | | |

---

Signif. codes: 0 ‘***’ 0.001 ‘**’ 0.01 ‘*’ 0.05 ‘.’ 0.1 ‘’ 1

$Barley_Root$`4`

Analysis of Variance Table

Response: Value

| | Df | Sum Sq | Mean Sq | F value | Pr(>F) | |
|---|---|---|---|---|---|---|
| Treatment | 3 | 10.14 | 3.38 | 1.8988 | 0.1568 | |
| Residuals | 24 | 42.721 | 1.7801 | | | |

$Barley_Root$`7`

Analysis of Variance Table

Response: Value

| | Df | Sum Sq | Mean Sq | F value | Pr(>F) | |
|---|---|---|---|---|---|---|
| Treatment | 3 | 13.743 | 4.581 | 2.8839 | 0.05665 | . |
| Residuals | 24 | 38.123 | 1.5884 | | | |

---

Signif. codes: 0 ‘***’ 0.001 ‘**’ 0.01 ‘*’ 0.05 ‘.’ 0.1 ‘’ 1

$Barley_Root$`9`

Analysis of Variance Table

Response: Value

| | Df | Sum Sq | Mean Sq | F value | Pr(>F) | |
|---|---|---|---|---|---|---|
| Treatment | 3 | 4.997 | 1.6658 | 0.8266 | 0.4922 | |
| Residuals | 24 | 48.367 | 2.0153 | | | |

$Barley_Root$`11`

Analysis of Variance Table

Response: Value

| | Df | Sum Sq | Mean Sq | F value | Pr(>F) | |
|---|---|---|---|---|---|---|
| Treatment | 3 | 13.185 | 4.3949 | 6.378 | 0.002472 | ** |
| Residuals | 24 | 16.538 | 0.6891 | | | |

---

Signif. codes: 0 '***' 0.001 '**' 0.01 '*' 0.05 '.' 0.1 '' 1

$Barley_Root$`14`

Analysis of Variance Table

Response: Value

| | Df | Sum Sq | Mean Sq | F value | Pr(>F) | |
|---|---|---|---|---|---|---|
| Treatment | 3 | 15.37 | 5.1232 | 5.8947 | 0.003657 | ** |
| Residuals | 24 | 20.859 | 0.8691 | | | |

---

Signif. codes: 0 '***' 0.001 '**' 0.01 '*' 0.05 '.' 0.1 '' 1

***$Barley_Shoot***

$Barley_Shoot$`0`

Analysis of Variance Table

Response: Value

| | Df | Sum Sq | Mean Sq | F value | Pr(>F) |
|---|---|---|---|---|---|
| Treatment | 3 | 0.2588 | 0.08627 | 0.1898 | 0.9023 |
| Residuals | 24 | 10.9089 | 0.45454 | | |

$Barley_Shoot$`2`

Analysis of Variance Table

Response: Value

| | Df | Sum Sq | Mean Sq | F value | Pr(>F) |
|---|---|---|---|---|---|
| Treatment | 3 | 3.3171 | 1.1057 | 1.3166 | 0.2921 |
| Residuals | 24 | 20.1558 | 0.83983 | | |

$Barley_Shoot$`4`

Analysis of Variance Table

Response: Value

| | Df | Sum Sq | Mean Sq | F value | Pr(>F) |
|---|---|---|---|---|---|
| Treatment | 3 | 0.4648 | 0.15493 | 0.4647 | 0.7096 |
| Residuals | 24 | 8.0017 | 0.33341 | | |

$Barley_Shoot$`7`

Analysis of Variance Table

Response: Value

| | Df | Sum Sq | Mean Sq | F value | Pr(>F) |
|---|---|---|---|---|---|
| Treatment | 3 | 0.9773 | 0.32576 | 1.4361 | 0.2569 |

| | | | | | |
|---|---|---|---|---|---|
| Residuals | 24 | 5.4439 | 0.22683 | | |

$Barley_Shoot$`9`
Analysis of Variance Table

Response: Value

| | Df | Sum Sq | Mean Sq | F value | Pr(>F) |
|---|---|---|---|---|---|
| Treatment | 3 | 0.2196 | 0.073195 | 0.4366 | 0.7288 |
| Residuals | 24 | 4.0232 | 0.167633 | | |

$Barley_Shoot$`11`
Analysis of Variance Table

Response: Value

| | Df | Sum Sq | Mean Sq | F value | Pr(>F) |
|---|---|---|---|---|---|
| Treatment | 3 | 0.7281 | 0.24269 | 1.4137 | 0.2632 |
| Residuals | 24 | 4.1201 | 0.17167 | | |

$Barley_Shoot$`14`
Analysis of Variance Table

Response: Value

| | Df | Sum Sq | Mean Sq | F value | Pr(>F) |
|---|---|---|---|---|---|
| Treatment | 3 | 0.39598 | 0.13199 | 1.2751 | 0.3054 |
| Residuals | 24 | 2.48449 | 0.10352 | | |

***$Cucumber_Root***
$Cucumber_Root$`0`
Analysis of Variance Table

Response: Value

| | Df | Sum Sq | Mean Sq | F value | Pr(>F) |
|---|---|---|---|---|---|
| Treatment | 3 | 3.854 | 1.2846 | 0.9165 | 0.4478 |
| Residuals | 24 | 33.638 | 1.4016 | | |

$Cucumber_Root$`2`
Analysis of Variance Table

Response: Value

| | Df | Sum Sq | Mean Sq | F value | Pr(>F) |
|---|---|---|---|---|---|
| Treatment | 3 | 4.0057 | 1.33524 | 1.7976 | 0.1746 |
| Residuals | 24 | 17.8269 | 0.74279 | | |

$Cucumber_Root$`5`
Analysis of Variance Table

Response: Value

| | Df | Sum Sq | Mean Sq | F value | Pr(>F) |
|---|---|---|---|---|---|
| Treatment | 3 | 0.9677 | 0.32257 | 0.506 | 0.6818 |

| | | | | | | |
|---|---|---|---|---|---|---|
| Residuals | 24 | 15.2999 | 0.6375 | | | |

$Cucumber_Root$`7`
Analysis of Variance Table

Response: Value

| | Df | Sum Sq | Mean Sq | F value | Pr(>F) | |
|---|---|---|---|---|---|---|
| Treatment | 3 | 1.2188 | 0.40626 | 0.9539 | 0.4304 | |
| Residuals | 24 | 10.2218 | 0.42591 | | | |

$Cucumber_Root$`9`
Analysis of Variance Table

Response: Value

| | Df | Sum Sq | Mean Sq | F value | Pr(>F) | |
|---|---|---|---|---|---|---|
| Treatment | 3 | 0.1776 | 0.05919 | 0.0797 | 0.9704 | |
| Residuals | 24 | 17.8253 | 0.74272 | | | |

$Cucumber_Root$`12`
Analysis of Variance Table

Response: Value

| | Df | Sum Sq | Mean Sq | F value | Pr(>F) | |
|---|---|---|---|---|---|---|
| Treatment | 3 | 18.259 | 6.0863 | 6.6115 | 0.002055 | ** |
| Residuals | 24 | 22.093 | 0.9206 | | | |

---
Signif. codes: 0 ‘***’ 0.001 ‘**’ 0.01 ‘*’ 0.05 ‘.’ 0.1 ‘’ 1

$Cucumber_Root$`14`
Analysis of Variance Table

Response: Value

| | Df | Sum Sq | Mean Sq | F value | Pr(>F) | |
|---|---|---|---|---|---|---|
| Treatment | 3 | 36.752 | 12.2508 | 7.7916 | 0.00084 | *** |
| Residuals | 24 | 37.736 | 1.5723 | | | |

---
Signif. codes: 0 ‘***’ 0.001 ‘**’ 0.01 ‘*’ 0.05 ‘.’ 0.1 ‘’ 1

***$Cucumber_Shoot***

$Cucumber_Shoot$`0`
Analysis of Variance Table

Response: Value

| | Df | Sum Sq | Mean Sq | F value | Pr(>F) | |
|---|---|---|---|---|---|---|
| Treatment | 3 | 2.5145 | 0.83816 | 0.902 | 0.4547 | |
| Residuals | 24 | 22.3005 | 0.92919 | | | |

$Cucumber_Shoot$`2`
Analysis of Variance Table

Response: Value

| | Df | Sum Sq | Mean Sq | F value | Pr(>F) | |
|---|---|---|---|---|---|---|
| Treatment | 3 | 0.6734 | 0.22446 | 0.5215 | 0.6716 | |
| Residuals | 24 | 10.33 | 0.43042 | | | |

$Cucumber_Shoot$`5`
Analysis of Variance Table

Response: Value

| | Df | Sum Sq | Mean Sq | F value | Pr(>F) | |
|---|---|---|---|---|---|---|
| Treatment | 3 | 0.3255 | 0.1085 | 0.3827 | 0.7663 | |
| Residuals | 24 | 6.8034 | 0.28347 | | | |

$Cucumber_Shoot$`7`
Analysis of Variance Table

Response: Value

| | Df | Sum Sq | Mean Sq | F value | Pr(>F) | |
|---|---|---|---|---|---|---|
| Treatment | 3 | 0.6013 | 0.20044 | 0.6791 | 0.5734 | |
| Residuals | 24 | 7.0834 | 0.29514 | | | |

$Cucumber_Shoot$`9`
Analysis of Variance Table

Response: Value

| | Df | Sum Sq | Mean Sq | F value | Pr(>F) | |
|---|---|---|---|---|---|---|
| Treatment | 3 | 0.8343 | 0.2781 | 0.795 | 0.5087 | |
| Residuals | 24 | 8.3954 | 0.34981 | | | |

$Cucumber_Shoot$`12`
Analysis of Variance Table

Response: Value

| | Df | Sum Sq | Mean Sq | F value | Pr(>F) | |
|---|---|---|---|---|---|---|
| Treatment | 3 | 23.982 | 7.9939 | 11.485 | 7.28E-05 | *** |
| Residuals | 24 | 16.705 | 0.696 | | | |

---
Signif. codes: 0 ‘***’ 0.001 ‘**’ 0.01 ‘*’ 0.05 ‘.’ 0.1 ‘’ 1

$Cucumber_Shoot$`14`
Analysis of Variance Table

Response: Value

| | Df | Sum Sq | Mean Sq | F value | Pr(>F) | |
|---|---|---|---|---|---|---|
| Treatment | 3 | 29.391 | 9.797 | 13.52 | 2.27E-05 | *** |
| Residuals | 24 | 17.391 | 0.7246 | | | |

---
Signif. codes: 0 ‘***’ 0.001 ‘**’ 0.01 ‘*’ 0.05 ‘.’ 0.1 ‘’ 1

***$Maize_Root***

$Maize_Root$`0`

Analysis of Variance Table

Response: Value

| | Df | Sum Sq | Mean Sq | F value | Pr(>F) |
|---|---|---|---|---|---|
| Treatment | 3 | 0.4681 | 0.15602 | 1.1002 | 0.3683 |
| Residuals | 24 | 3.4036 | 0.14182 | | |

$Maize_Root$`2`

Analysis of Variance Table

Response: Value

| | Df | Sum Sq | Mean Sq | F value | Pr(>F) |
|---|---|---|---|---|---|
| Treatment | 3 | 0.8522 | 0.28406 | 0.5512 | 0.6522 |
| Residuals | 24 | 12.3676 | 0.51532 | | |

$Maize_Root$`4`

Analysis of Variance Table

Response: Value

| | Df | Sum Sq | Mean Sq | F value | Pr(>F) |
|---|---|---|---|---|---|
| Treatment | 3 | 1.2925 | 0.43082 | 0.7651 | 0.5248 |
| Residuals | 24 | 13.514 | 0.56308 | | |

$Maize_Root$`7`

Analysis of Variance Table

Response: Value

| | Df | Sum Sq | Mean Sq | F value | Pr(>F) |
|---|---|---|---|---|---|
| Treatment | 3 | 0.8029 | 0.26762 | 0.6289 | 0.6035 |
| Residuals | 24 | 10.2136 | 0.42557 | | |

$Maize_Root$`9`

Analysis of Variance Table

Response: Value

| | Df | Sum Sq | Mean Sq | F value | Pr(>F) |
|---|---|---|---|---|---|
| Treatment | 3 | 1.4042 | 0.46805 | 1.8566 | 0.1639 |
| Residuals | 24 | 6.0503 | 0.2521 | | |

$Maize_Root$`11`

Analysis of Variance Table

Response: Value

| | Df | Sum Sq | Mean Sq | F value | Pr(>F) |
|---|---|---|---|---|---|
| Treatment | 3 | 2.4327 | 0.81089 | 1.3521 | 0.2812 |
| Residuals | 24 | 14.3936 | 0.59973 | | |

$Maize_Root$`14`
Analysis of Variance Table

Response: Value

| | Df | Sum Sq | Mean Sq | F value | Pr(>F) | |
|---|---|---|---|---|---|---|
| Treatment | 3 | 2.2051 | 0.73504 | 6.2618 | 0.002713 | ** |
| Residuals | 24 | 2.8172 | 0.11738 | | | |

---
Signif. codes: 0 ‘***’ 0.001 ‘**’ 0.01 ‘*’ 0.05 ‘.’ 0.1 ‘’ 1

***$Maize_Shoot***
$Maize_Shoot$`0`
Analysis of Variance Table

Response: Value

| | Df | Sum Sq | Mean Sq | F value | Pr(>F) |
|---|---|---|---|---|---|
| Treatment | 3 | 1.7203 | 0.57344 | 0.945 | 0.4345 |
| Residuals | 24 | 14.5637 | 0.60682 | | |

$Maize_Shoot$`2`
Analysis of Variance Table

Response: Value

| | Df | Sum Sq | Mean Sq | F value | Pr(>F) |
|---|---|---|---|---|---|
| Treatment | 3 | 0.3464 | 0.11546 | 0.4146 | 0.744 |
| Residuals | 24 | 6.6835 | 0.27848 | | |

$Maize_Shoot$`4`
Analysis of Variance Table

Response: Value

| | Df | Sum Sq | Mean Sq | F value | Pr(>F) | |
|---|---|---|---|---|---|---|
| Treatment | 3 | 5.1688 | 1.72292 | 6.4209 | 0.002389 | ** |
| Residuals | 24 | 6.4399 | 0.26833 | | | |

---
Signif. codes: 0 ‘***’ 0.001 ‘**’ 0.01 ‘*’ 0.05 ‘.’ 0.1 ‘’ 1

$Maize_Shoot$`7`
Analysis of Variance Table

Response: Value

| | Df | Sum Sq | Mean Sq | F value | Pr(>F) | |
|---|---|---|---|---|---|---|
| Treatment | 3 | 2.9124 | 0.97079 | 2.8546 | 0.05834 | . |
| Residuals | 24 | 8.1619 | 0.34008 | | | |

---
Signif. codes: 0 ‘***’ 0.001 ‘**’ 0.01 ‘*’ 0.05 ‘.’ 0.1 ‘’ 1

$Maize_Shoot$`9`

Analysis of Variance Table

Response: Value

| | Df | Sum Sq | Mean Sq | F value | Pr(>F) | |
|---|---|---|---|---|---|---|
| Treatment | 3 | 8.1765 | 2.72551 | 22.783 | 3.33E-07 | *** |
| Residuals | 24 | 2.8711 | 0.11963 | | | |

---
Signif. codes: 0 ‘***’ 0.001 ‘**’ 0.01 ‘*’ 0.05 ‘.’ 0.1 ‘’ 1

$Maize_Shoot$`11`

Analysis of Variance Table

Response: Value

| | Df | Sum Sq | Mean Sq | F value | Pr(>F) | |
|---|---|---|---|---|---|---|
| Treatment | 3 | 8.6452 | 2.88172 | 19.949 | 1.05E-06 | *** |
| Residuals | 24 | 3.4669 | 0.14445 | | | |

---
Signif. codes: 0 ‘***’ 0.001 ‘**’ 0.01 ‘*’ 0.05 ‘.’ 0.1 ‘’ 1

$Maize_Shoot$`14`

Analysis of Variance Table

Response: Value

| | Df | Sum Sq | Mean Sq | F value | Pr(>F) | |
|---|---|---|---|---|---|---|
| Treatment | 3 | 3.3416 | 1.11387 | 16.118 | 5.96E-06 | *** |
| Residuals | 24 | 1.6586 | 0.06911 | | | |

---
Signif. codes: 0 ‘***’ 0.001 ‘**’ 0.01 ‘*’ 0.05 ‘.’ 0.1 ‘’ 1

***$Tomato_Root***

$Tomato_Root$`0`

Analysis of Variance Table

Response: Value

| | Df | Sum Sq | Mean Sq | F value | Pr(>F) | |
|---|---|---|---|---|---|---|
| Treatment | 3 | 10.098 | 3.3661 | 6.4677 | 0.002302 | ** |
| Residuals | 24 | 12.491 | 0.5204 | | | |

---
Signif. codes: 0 ‘***’ 0.001 ‘**’ 0.01 ‘*’ 0.05 ‘.’ 0.1 ‘’ 1

$Tomato_Root$`1`

Analysis of Variance Table

Response: Value

| | Df | Sum Sq | Mean Sq | F value | Pr(>F) |
|---|---|---|---|---|---|
| Treatment | 3 | 6.5809 | 2.1936 | 1.968 | 0.1457 |
| Residuals | 24 | 26.751 | 1.1146 | | |

$Tomato_Root$`3`

Analysis of Variance Table

Response: Value

| | Df | Sum Sq | Mean Sq | F value | Pr(>F) | |
|---|---|---|---|---|---|---|
| Treatment | 3 | 5.1066 | 1.70221 | 4.6321 | 0.0108 | * |
| Residuals | 24 | 8.8196 | 0.36748 | | | |

---

Signif. codes: 0 ‘***’ 0.001 ‘**’ 0.01 ‘*’ 0.05 ‘.’ 0.1 ‘’ 1

$Tomato_Root$`7`

Analysis of Variance Table

Response: Value

| | Df | Sum Sq | Mean Sq | F value | Pr(>F) | |
|---|---|---|---|---|---|---|
| Treatment | 3 | 8.5644 | 2.85478 | 11.415 | 7.60E-05 | *** |
| Residuals | 24 | 6.0021 | 0.25009 | | | |

---

Signif. codes: 0 ‘***’ 0.001 ‘**’ 0.01 ‘*’ 0.05 ‘.’ 0.1 ‘’ 1

$Tomato_Root$`9`

Analysis of Variance Table

Response: Value

| | Df | Sum Sq | Mean Sq | F value | Pr(>F) | |
|---|---|---|---|---|---|---|
| Treatment | 3 | 24.0309 | 8.0103 | 20.843 | 7.21E-07 | *** |
| Residuals | 24 | 9.2237 | 0.3843 | | | |

---

Signif. codes: 0 ‘***’ 0.001 ‘**’ 0.01 ‘*’ 0.05 ‘.’ 0.1 ‘’ 1

$Tomato_Root$`11`

Analysis of Variance Table

Response: Value

| | Df | Sum Sq | Mean Sq | F value | Pr(>F) | |
|---|---|---|---|---|---|---|
| Treatment | 3 | 35.028 | 11.6761 | 104.75 | 6.34E-14 | *** |
| Residuals | 24 | 2.675 | 0.1115 | | | |

---

Signif. codes: 0 ‘***’ 0.001 ‘**’ 0.01 ‘*’ 0.05 ‘.’ 0.1 ‘’ 1

$Tomato_Root$`14`

Analysis of Variance Table

Response: Value

| | Df | Sum Sq | Mean Sq | F value | Pr(>F) | |
|---|---|---|---|---|---|---|
| Treatment | 3 | 35.192 | 11.7305 | 86.404 | 5.31E-13 | *** |
| Residuals | 24 | 3.258 | 0.1358 | | | |

---

Signif. codes: 0 ‘***’ 0.001 ‘**’ 0.01 ‘*’ 0.05 ‘.’ 0.1 ‘’ 1

***$Tomato_Shoot***

$Tomato_Shoot$`0`

Analysis of Variance Table

Response: Value

| | Df | Sum Sq | Mean Sq | F value | Pr(>F) | |
|---|---|---|---|---|---|---|
| Treatment | 3 | 9.4434 | 3.1478 | 4.0538 | 0.01829 | * |
| Residuals | 24 | 18.6359 | 0.7765 | | | |

\---

Signif. codes: 0 ‘***’ 0.001 ‘**’ 0.01 ‘*’
0.05 ‘.’ 0.1 ” 1

$Tomato_Shoot$`1`

Analysis of Variance Table

Response: Value

| | Df | Sum Sq | Mean Sq | F value | Pr(>F) | |
|---|---|---|---|---|---|---|
| Treatment | 3 | 10.699 | 3.5665 | 3.4713 | 0.03176 | * |
| Residuals | 24 | 24.658 | 1.0274 | | | |

\---

Signif. codes: 0 ‘***’ 0.001 ‘**’ 0.01 ‘*’
0.05 ‘.’ 0.1 ” 1

$Tomato_Shoot$`3`

Analysis of Variance Table

Response: Value

| | Df | Sum Sq | Mean Sq | F value | Pr(>F) |
|---|---|---|---|---|---|
| Treatment | 3 | 0.9009 | 0.3003 | 2.1882 | 0.1156 |
| Residuals | 24 | 3.2937 | 0.13724 | | |

$Tomato_Shoot$`7`

Analysis of Variance Table

Response: Value

| | Df | Sum Sq | Mean Sq | F value | Pr(>F) | |
|---|---|---|---|---|---|---|
| Treatment | 3 | 6.7301 | 2.24338 | 12.966 | 3.09E-05 | *** |
| Residuals | 24 | 4.1524 | 0.17302 | | | |

\---

Signif. codes: 0 ‘***’ 0.001 ‘**’ 0.01 ‘*’
0.05 ‘.’ 0.1 ” 1

$Tomato_Shoot$`9`

Analysis of Variance Table

Response: Value

| | Df | Sum Sq | Mean Sq | F value | Pr(>F) | F | value |
|---|---|---|---|---|---|---|---|
| Treatment | 3 | 19.57 | 6.5232 | 27.778 | 5.61E-08 | *** | |
| Residuals | 24 | 5.636 | 0.2348 | | | | |

\---

Signif. codes: 0 ‘***’ 0.001 ‘**’ 0.01 ‘*’
0.05 ‘.’ 0.1 ” 1

$Tomato_Shoot$`11`

Analysis of Variance Table

Response: Value

| | Df | Sum Sq | Mean Sq | F value | Pr(>F) | |
|---|---|---|---|---|---|---|
| Treatment | 3 | 34.947 | 11.6491 | 107.13 | 4.94E-14 | *** |
| Residuals | 24 | 2.61 | 0.1087 | | | |

---

Signif. codes: 0 ‘***’ 0.001 ‘**’ 0.01 ‘*’ 0.05 ‘.’ 0.1 ‘’ 1

$Tomato_Shoot$`14`

Analysis of Variance Table

Response: Value

| | Df | Sum Sq | Mean Sq | F value | Pr(>F) | |
|---|---|---|---|---|---|---|
| Treatment | 3 | 36.226 | 12.0753 | 88.237 | 4.22E-13 | *** |
| Residuals | 24 | 3.284 | 0.1369 | | | |

---

Signif. codes: 0 ‘***’ 0.001 ‘**’ 0.01 ‘*’ 0.05 ‘.’ 0.1 ‘’ 1

**Tukey.HSD multiple comparison on treatments**

| ***Between treatments differences*** | BR | | | | | | |
|---|---|---|---|---|---|---|---|
| ***Treatment effect*** | 0 | 2 | 4 | 7 | 9 | 11 | 14 |
| Tukey | P = 0.676 | P = 0.007 | P = 0.157 | P = 0.057 | P = 0.492 | P = 0.002 | P = 0.004 |
| C | a | a | a | ab | a | ab | ab |
| -P | a | ab | a | ab | a | b | b |
| -Fe | a | a | a | a | a | a | a |
| -P/-Fe | a | b | a | b | a | b | b |

| ***Between treatments differences*** | BS --> not to test (P=0.212) | | | | | | |
|---|---|---|---|---|---|---|---|
| ***Treatment effect*** | 0 | 2 | 4 | 7 | 9 | 11 | 14 |
| Tukey | P = 0.902 | P = 0.292 | P = 0.710 | P = 0.257 | P = 0.729 | P = 0.263 | P = 0.305 |
| C | a | a | a | a | a | a | a |
| -P | a | a | a | a | a | a | a |
| -Fe | a | a | a | a | a | a | a |
| -P/-Fe | a | a | a | a | a | a | a |

| ***Between treatments differences*** | CR | | | | | | |
|---|---|---|---|---|---|---|---|
| ***Treatment effect*** | 0 | 2 | 5 | 7 | 9 | 12 | 14 |
| Tukey | P = 0.448 | P = 0.175 | P = 0.682 | P = 0.430 | P = 0.970 | P = 0.002 | P < 0.001 |
| C | a | a | a | a | a | c | b |
| -P | a | a | a | a | a | a | a |
| -Fe | a | a | a | a | a | bc | b |
| -P/-Fe | a | a | a | a | a | ab | a |

| ***Between treatments differences*** | CS | | | | | | |
|---|---|---|---|---|---|---|---|
| ***Treatment effect*** | 0 | 2 | 5 | 7 | 9 | 12 | 14 |
| Tukey | P = 0.455 | P = 0.672 | P = 0.766 | P = 0.573 | P = 0.509 | P < 0.001 | P < 0.001 |
| C | a | a | a | a | a | b | bc |
| -P | a | a | a | a | a | a | a |
| -Fe | a | a | a | a | a | b | c |
| -P/-Fe | a | a | a | a | a | a | ab |

| ***Between treatments differences*** | MR --> not to test (P=0.543) | | | | | | |
|---|---|---|---|---|---|---|---|
| ***Treatment effect*** | 0 | 2 | 4 | 7 | 9 | 11 | 14 |
| Tukey | P = 0.368 | P = 0.652 | P = 0.525 | P = 0.604 | P = 0.164 | P = 0.281 | P = 0.003 |
| C | a | a | a | a | a | a | a |
| -P | a | a | a | a | a | a | a |
| -Fe | a | a | a | a | a | a | ab |
| -P/-Fe | a | a | a | a | a | a | b |

| ***Between treatments differences*** | MS | | | | | | |
|---|---|---|---|---|---|---|---|
| ***Treatment effect*** | 0 | 2 | 4 | 7 | 9 | 11 | 14 |
| Tukey | P = 0.435 | P = 0.744 | P = 0.002 | P = 0.058 | P < 0.001 | P < 0.001 | P < 0.001 |
| C | a | a | b | a | b | c | b |
| -P | a | a | b | a | b | bc | b |
| -Fe | a | a | a | a | a | a | a |

| | | | | | | | |
|---|---|---|---|---|---|---|---|
| -P/-Fe | a | a | ab | a | a | b | a |

***Between treatments differences*** TR

| ***Treatment effect*** | 0 | 1 | 3 | 7 | 9 | 11 | 14 |
|---|---|---|---|---|---|---|---|
| Tukey | P = 0.002 | P = 0.145 | P = 0.011 | P < 0.001 | P < 0.001 | P < 0.001 | P < 0.001 |
| C | a | a | ab | b | bc | c | c |
| -P | b | a | a | a | a | a | a |
| -Fe | b | a | b | b | c | c | d |
| -P/-Fe | b | a | ab | b | b | b | b |

***Between treatments differences*** TS

| ***Treatment effect*** | 0 | 1 | 3 | 7 | 9 | 11 | 14 |
|---|---|---|---|---|---|---|---|
| Tukey | P = 0.018 | P = 0.032 | P = 0.116 | P < 0.001 | P < 0.001 | P < 0.001 | P < 0.001 |
| C | a | ab | a | b | b | c | b |
| -P | b | ab | a | a | a | a | a |
| -Fe | b | b | a | b | b | c | b |
| -P/-Fe | ab | a | a | a | a | b | a |

**One-Way ANOVA on time**

***$Barley_Root***

$Barley_Root$C

Analysis of Variance Table

Response: Value

| | Df | Sum Sq | Mean Sq | F value | Pr(>F) | | |
|---|---|---|---|---|---|---|---|
| Time | 6 | 495.73 | 82.622 | 70.456 | < | 2.20E-16 | *** |
| Residuals | 42 | 49.25 | 1.173 | | | | |

---

Signif. codes: 0 ‘***’ 0.001 ‘**’ 0.01 ‘*’ 0.05 ‘.’ 0.1 ‘’ 1

$Barley_Root$Fe

Analysis of Variance Table

Response: Value

| | Df | Sum Sq | Mean Sq | F value | Pr(>F) | |
|---|---|---|---|---|---|---|
| Time | 6 | 403.17 | 67.195 | 40.813 | 5.61E-16 | *** |
| Residuals | 42 | 69.15 | 1.646 | | | |

---

Signif. codes: 0 ‘***’ 0.001 ‘**’ 0.01 ‘*’ 0.05 ‘.’ 0.1 ‘’ 1

$Barley_Root$P

Analysis of Variance Table

Response: Value

| | Df | Sum Sq | Mean Sq | F value | Pr(>F) | | |
|---|---|---|---|---|---|---|---|
| Time | 6 | 490.54 | 81.757 | 82.481 | < | 2.20E-16 | *** |
| Residuals | 42 | 41.63 | 0.991 | | | | |

---

Signif. codes: 0 ‘***’ 0.001 ‘**’ 0.01 ‘*’ 0.05 ‘.’ 0.1 ‘’ 1

$Barley_Root$`P/Fe`

Analysis of Variance Table

Response: Value

| | Df | Sum Sq | Mean Sq | F value | Pr(>F) | | |
|---|---|---|---|---|---|---|---|
| Time | 6 | 531.43 | 88.571 | 69.639 | < | 2.20E-16 | *** |
| Residuals | 42 | 53.42 | 1.272 | | | | |

---

Signif. codes: 0 ‘***’ 0.001 ‘**’ 0.01 ‘*’ 0.05 ‘.’ 0.1 ‘’ 1

***$Barley_Shoot***

$Barley_Shoot$C

Analysis of Variance Table

Response: Value

| | Df | Sum Sq | Mean Sq | F value | Pr(>F) | | |
|---|---|---|---|---|---|---|---|
| Time | 6 | 394.03 | 65.671 | 167.14 | < | 2.20E-16 | *** |
| Residuals | 42 | 16.5 | 0.393 | | | | |
| --- | | | | | | | |
| Signif. | codes: | 0 | ‘***’ | 0.001 | ‘**’ | 0.01 | ‘*’ |
| | | 0.05 | ‘.’ | 0.1 | ‘’ | 1 | |

$Barley_Shoot$Fe
Analysis of Variance Table

Response: Value

| | Df | Sum Sq | Mean Sq | F value | Pr(>F) | | |
|---|---|---|---|---|---|---|---|
| Time | 6 | 379.74 | 63.29 | 158.08 | < | 2.20E-16 | *** |
| Residuals | 42 | 16.82 | 0.4 | | | | |
| --- | | | | | | | |
| Signif. | codes: | 0 | ‘***’ | 0.001 | ‘**’ | 0.01 | ‘*’ |
| | | 0.05 | ‘.’ | 0.1 | ‘’ | 1 | |

$Barley_Shoot$P
Analysis of Variance Table

Response: Value

| | Df | Sum Sq | Mean Sq | F value | Pr(>F) | | |
|---|---|---|---|---|---|---|---|
| Time | 6 | 375.04 | 62.507 | 274.21 | < | 2.20E-16 | *** |
| Residuals | 42 | 9.57 | 0.228 | | | | |
| --- | | | | | | | |
| Signif. | codes: | 0 | ‘***’ | 0.001 | ‘**’ | 0.01 | ‘*’ |
| | | 0.05 | ‘.’ | 0.1 | ‘’ | 1 | |

$Barley_Shoot$`P/Fe`
Analysis of Variance Table

Response: Value

| | Df | Sum Sq | Mean Sq | F value | Pr(>F) | | |
|---|---|---|---|---|---|---|---|
| Time | 6 | 369.19 | 61.532 | 211.03 | < | 2.20E-16 | *** |
| Residuals | 42 | 12.25 | 0.292 | | | | |
| --- | | | | | | | |
| Signif. | codes: | 0 | ‘***’ | 0.001 | ‘**’ | 0.01 | ‘*’ |
| | | 0.05 | ‘.’ | 0.1 | ‘’ | 1 | |

***$Cucumber_Root***

$Cucumber_Root$C
Analysis of Variance Table

Response: Value

| | Df | Sum Sq | Mean Sq | F value | Pr(>F) | | |
|---|---|---|---|---|---|---|---|
| Time | 6 | 71.131 | 11.855 | 17.358 | 5.66E-10 | *** | |
| Residuals | 42 | 28.686 | 0.683 | | | | |
| --- | | | | | | | |
| Signif. | codes: | 0 | ‘***’ | 0.001 | ‘**’ | 0.01 | ‘*’ |
| | | 0.05 | ‘.’ | 0.1 | ‘’ | 1 | |

$Cucumber_Root$Fe

Analysis of Variance Table

Response: Value

| | Df | Sum Sq | Mean Sq | F value | Pr(>F) | |
|---|---|---|---|---|---|---|
| Time | 6 | 53.714 | 8.9523 | 12.528 | 4.82E-08 | *** |
| Residuals | 42 | 30.013 | 0.7146 | | | |

---

Signif. codes: 0 ‘***’ 0.001 ‘**’ 0.01 ‘*’ 0.05 ‘.’ 0.1 ‘’ 1

$Cucumber_Root$P

Analysis of Variance Table

Response: Value

| | Df | Sum Sq | Mean Sq | F value | Pr(>F) | |
|---|---|---|---|---|---|---|
| Time | 6 | 56.948 | 9.4913 | 9.6459 | 1.14E-06 | *** |
| Residuals | 42 | 41.327 | 0.984 | | | |

---

Signif. codes: 0 ‘***’ 0.001 ‘**’ 0.01 ‘*’ 0.05 ‘.’ 0.1 ‘’ 1

$Cucumber_Root$`P/Fe`

Analysis of Variance Table

Response: Value

| | Df | Sum Sq | Mean Sq | F value | Pr(>F) | |
|---|---|---|---|---|---|---|
| Time | 6 | 39.422 | 6.5704 | 5.0527 | 0.000558 | *** |
| Residuals | 42 | 54.615 | 1.3004 | | | |

---

Signif. codes: 0 ‘***’ 0.001 ‘**’ 0.01 ‘*’ 0.05 ‘.’ 0.1 ‘’ 1

***$Cucumber_Shoot***

$Cucumber_Shoot$C

Analysis of Variance Table

Response: Value

| | Df | Sum Sq | Mean Sq | F value | Pr(>F) | |
|---|---|---|---|---|---|---|
| Time | 6 | 71.091 | 11.8485 | 26.069 | 1.11E-12 | *** |
| Residuals | 42 | 19.089 | 0.4545 | | | |

---

Signif. codes: 0 ‘***’ 0.001 ‘**’ 0.01 ‘*’ 0.05 ‘.’ 0.1 ‘’ 1

$Cucumber_Shoot$Fe

Analysis of Variance Table

Response: Value

| | Df | Sum Sq | Mean Sq | F value | Pr(>F) | |
|---|---|---|---|---|---|---|
| Time | 6 | 78.263 | 13.0439 | 41.707 | 3.83E-16 | *** |
| Residuals | 42 | 13.136 | 0.3128 | | | |

---

Signif. codes: 0 ‘***’ 0.001 ‘**’ 0.01 ‘*’

0.05 ‘.’ 0.1 ‘’ 1

$Cucumber_Shoot$P

Analysis of Variance Table

Response: Value

| | Df | Sum Sq | Mean Sq | F value | Pr(>F) | |
|---|---|---|---|---|---|---|
| Time | 6 | 60.055 | 10.0091 | 16.428 | 1.24E-09 | *** |
| Residuals | 42 | 25.59 | 0.6093 | | | |

---

Signif. codes: 0 ‘***’ 0.001 ‘**’ 0.01 ‘*’
0.05 ‘.’ 0.1 ‘’ 1

$Cucumber_Shoot$`P/Fe`

Analysis of Variance Table

Response: Value

| | Df | Sum Sq | Mean Sq | F value | Pr(>F) | |
|---|---|---|---|---|---|---|
| Time | 6 | 33.115 | 5.5192 | 7.4311 | 1.84E-05 | *** |
| Residuals | 42 | 31.194 | 0.7427 | | | |

---

Signif. codes: 0 ‘***’ 0.001 ‘**’ 0.01 ‘*’
0.05 ‘.’ 0.1 ‘’ 1

***$Maize_Root***

$Maize_Root$C

Analysis of Variance Table

Response: Value

| | Df | Sum Sq | Mean Sq | F value | Pr(>F) | | |
|---|---|---|---|---|---|---|---|
| Time | 6 | 95.407 | 15.9012 | 61.464 | < | 2.20E-16 | *** |
| Residuals | 42 | 10.866 | 0.2587 | | | | |

---

Signif. codes: 0 ‘***’ 0.001 ‘**’ 0.01 ‘*’
0.05 ‘.’ 0.1 ‘’ 1

$Maize_Root$Fe

Analysis of Variance Table

Response: Value

| | Df | Sum Sq | Mean Sq | F value | Pr(>F) | |
|---|---|---|---|---|---|---|
| Time | 6 | 74.39 | 12.3984 | 23.163 | 7.31E-12 | *** |
| Residuals | 42 | 22.481 | 0.5353 | | | |

---

Signif. codes: 0 ‘***’ 0.001 ‘**’ 0.01 ‘*’
0.05 ‘.’ 0.1 ‘’ 1

$Maize_Root$P

Analysis of Variance Table

Response: Value

| | Df | Sum Sq | Mean Sq | F value | Pr(>F) | |
|---|---|---|---|---|---|---|
| Time | 6 | 85.519 | 14.2531 | 40.537 | 6.33E-16 | *** |
| Residuals | 42 | 14.768 | 0.3516 | | | |

---
Signif. codes: 0 ‘***’ 0.001 ‘**’ 0.01 ‘*’ 0.05 ‘.’ 0.1 ‘’ 1

$Maize_Root$`P/Fe`
Analysis of Variance Table

Response: Value

| | Df | Sum Sq | Mean Sq | F value | Pr(>F) | |
|---|---|---|---|---|---|---|
| Time | 6 | 87.695 | 14.6159 | 41.916 | 3.50E-16 | *** |
| Residuals | 42 | 14.645 | 0.3487 | | | |

---
Signif. codes: 0 ‘***’ 0.001 ‘**’ 0.01 ‘*’ 0.05 ‘.’ 0.1 ‘’ 1

***$Maize_Shoot***

$Maize_Shoot$C
Analysis of Variance Table

Response: Value

| | Df | Sum Sq | Mean Sq | F value | Pr(>F) | |
|---|---|---|---|---|---|---|
| Time | 6 | 106.318 | 17.7197 | 82.58 | < 2.20E-16 | *** |
| Residuals | 42 | 9.012 | 0.2146 | | | |

---
Signif. codes: 0 ‘***’ 0.001 ‘**’ 0.01 ‘*’ 0.05 ‘.’ 0.1 ‘’ 1

$Maize_Shoot$Fe
Analysis of Variance Table

Response: Value

| | Df | Sum Sq | Mean Sq | F value | Pr(>F) | |
|---|---|---|---|---|---|---|
| Time | 6 | 67.804 | 11.3007 | 47.86 | < 2.20E-16 | *** |
| Residuals | 42 | 9.917 | 0.2361 | | | |

---
Signif. codes: 0 ‘***’ 0.001 ‘**’ 0.01 ‘*’ 0.05 ‘.’ 0.1 ‘’ 1

$Maize_Shoot$P
Analysis of Variance Table

Response: Value

| | Df | Sum Sq | Mean Sq | F value | Pr(>F) | |
|---|---|---|---|---|---|---|
| Time | 6 | 100.118 | 16.6864 | 67.299 | < 2.20E-16 | *** |
| Residuals | 42 | 10.414 | 0.2479 | | | |

---
Signif. codes: 0 ‘***’ 0.001 ‘**’ 0.01 ‘*’ 0.05 ‘.’ 0.1 ‘’ 1

$Maize_Shoot$`P/Fe`
Analysis of Variance Table

Response: Value

| | Df | Sum Sq | Mean Sq | F value | Pr(>F) |
|---|---|---|---|---|---|

| | | | | | | | |
|---|---|---|---|---|---|---|---|
| Time | 6 | 97.882 | 16.3136 | 47.245 | < | 2.20E-16 | *** |
| Residuals | 42 | 14.503 | 0.3453 | | | | |
| --- | | | | | | | |
| Signif. | codes: | 0 | ‘***’ | 0.001 | ‘**’ | 0.01 | ‘*’ |
| | | 0.05 | ‘.’ | 0.1 | ‘’ | 1 | |

***$Tomato_Root***

$Tomato_Root$C

| | | | | | | | |
|---|---|---|---|---|---|---|---|
| Analysis | of | Variance | Table | | | | |
| | | | | | | | |
| Response: | Value | | | | | | |
| | Df | Sum Sq | Mean Sq | F value | Pr(>F) | | |
| Time | 6 | 247.73 | 41.288 | 112.02 | < | 2.20E-16 | *** |
| Residuals | 42 | 15.48 | 0.369 | | | | |
| --- | | | | | | | |
| Signif. | codes: | 0 | ‘***’ | 0.001 | ‘**’ | 0.01 | ‘*’ |
| | | 0.05 | ‘.’ | 0.1 | ‘’ | 1 | |

$Tomato_Root$Fe

| | | | | | | | |
|---|---|---|---|---|---|---|---|
| Analysis | of | Variance | Table | | | | |
| | | | | | | | |
| Response: | Value | | | | | | |
| | Df | Sum Sq | Mean Sq | F value | Pr(>F) | | |
| Time | 6 | 157.987 | 26.3312 | 150.96 | < | 2.20E-16 | *** |
| Residuals | 42 | 7.326 | 0.1744 | | | | |
| --- | | | | | | | |
| Signif. | codes: | 0 | ‘***’ | 0.001 | ‘**’ | 0.01 | ‘*’ |
| | | 0.05 | ‘.’ | 0.1 | ‘’ | 1 | |

$Tomato_Root$P

| | | | | | | | |
|---|---|---|---|---|---|---|---|
| Analysis | of | Variance | Table | | | | |
| | | | | | | | |
| Response: | Value | | | | | | |
| | Df | Sum Sq | Mean Sq | F value | Pr(>F) | | |
| Time | 6 | 61.602 | 10.2669 | 20.984 | 3.37E-11 | *** | |
| Residuals | 42 | 20.55 | 0.4893 | | | | |
| --- | | | | | | | |
| Signif. | codes: | 0 | ‘***’ | 0.001 | ‘**’ | 0.01 | ‘*’ |
| | | 0.05 | ‘.’ | 0.1 | ‘’ | 1 | |

$Tomato_Root$`P/Fe`

| | | | | | | | |
|---|---|---|---|---|---|---|---|
| Analysis | of | Variance | Table | | | | |
| | | | | | | | |
| Response: | Value | | | | | | |
| | Df | Sum Sq | Mean Sq | F value | Pr(>F) | | |
| Time | 6 | 102.547 | 17.0911 | 27.753 | 4.02E-13 | *** | |
| Residuals | 42 | 25.865 | 0.6158 | | | | |
| --- | | | | | | | |
| Signif. | codes: | 0 | ‘***’ | 0.001 | ‘**’ | 0.01 | ‘*’ |
| | | 0.05 | ‘.’ | 0.1 | ‘’ | 1 | |

***$Tomato_Shoot***

| | | | | | | | |
|---|---|---|---|---|---|---|---|
| $Tomato_Shoot$C | | | | | | | |
| Analysis | of | Variance | Table | | | | |
| | | | | | | | |
| Response: | Value | | | | | | |
| | Df | Sum Sq | Mean Sq | F value | Pr(>F) | | |
| Time | 6 | 134.47 | 22.4125 | 100.47 | < | 2.20E-16 | *** |
| Residuals | 42 | 9.37 | 0.2231 | | | | |
| --- | | | | | | | |
| Signif. | codes: | 0 | ‘***’ | 0.001 | ‘**’ | 0.01 | ‘*’ |
| | | 0.05 | ‘.’ | 0.1 | ‘’ | 1 | |
| $Tomato_Shoot$Fe | | | | | | | |
| Analysis | of | Variance | Table | | | | |
| | | | | | | | |
| Response: | Value | | | | | | |
| | Df | Sum Sq | Mean Sq | F value | Pr(>F) | | |
| Time | 6 | 64.524 | 10.7541 | 40.349 | 6.87E-16 | *** | |
| Residuals | 42 | 11.194 | 0.2665 | | | | |
| --- | | | | | | | |
| Signif. | codes: | 0 | ‘***’ | 0.001 | ‘**’ | 0.01 | ‘*’ |
| | | 0.05 | ‘.’ | 0.1 | ‘’ | 1 | |
| $Tomato_Shoot$P | | | | | | | |
| Analysis | of | Variance | Table | | | | |
| | | | | | | | |
| Response: | Value | | | | | | |
| | Df | Sum Sq | Mean Sq | F value | Pr(>F) | | |
| Time | 6 | 25.662 | 4.277 | 12.952 | 3.14E-08 | *** | |
| Residuals | 42 | 13.869 | 0.3302 | | | | |
| --- | | | | | | | |
| Signif. | codes: | 0 | ‘***’ | 0.001 | ‘**’ | 0.01 | ‘*’ |
| | | 0.05 | ‘.’ | 0.1 | ‘’ | 1 | |
| $Tomato_Shoot$`P/Fe` | | | | | | | |
| Analysis | of | Variance | Table | | | | |
| | | | | | | | |
| Response: | Value | | | | | | |
| | Df | Sum Sq | Mean Sq | F value | Pr(>F) | | |
| Time | 6 | 35.237 | 5.8728 | 8.8606 | 2.94E-06 | *** | |
| Residuals | 42 | 27.838 | 0.6628 | | | | |
| --- | | | | | | | |
| Signif. | codes: | 0 | ‘***’ | 0.001 | ‘**’ | 0.01 | ‘*’ |
| | | 0.05 | ‘.’ | 0.1 | ‘’ | 1 | |

**Tukey.HSD multiple comparison on time**

| ***Within treatments differences*** | BR | | | | | | | |
|---|---|---|---|---|---|---|---|---|
| ***Time effect*** | Tukey | 0 | 2 | 4 | 7 | 9 | 11 | 14 |
| C | P < 0.001 | a | a | b | c | c | cd | d |
| -P | P < 0.001 | a | b | c | cd | d | de | e |
| -Fe | P < 0.001 | a | a | b | bc | cd | cd | d |
| -P/-Fe | P < 0.001 | a | b | b | c | cd | cd | d |

| ***Within treatments differences*** | BS | | | | | | | |
|---|---|---|---|---|---|---|---|---|
| ***Time effect*** | Tukey | 0 | 2 | 4 | 7 | 9 | 11 | 14 |
| C | P < 0.001 | a | b | c | d | d | d | e |
| -P | P < 0.001 | a | b | c | d | de | ef | f |
| -Fe | P < 0.001 | a | b | c | d | d | d | e |
| -P/-Fe | P < 0.001 | a | b | c | d | d | de | e |

| ***Within treatments differences*** | CR | | | | | | | |
|---|---|---|---|---|---|---|---|---|
| ***Time effect*** | Tukey | 0 | 2 | 5 | 7 | 9 | 12 | 14 |
| C | P < 0.001 | a | b | bc | c | bc | bc | bc |
| -P | P < 0.001 | a | bcd | cd | d | cd | abc | ab |
| -Fe | P < 0.001 | a | b | b | b | b | b | b |
| -P/-Fe | P < 0.001 | a | abc | c | c | bc | abc | ab |

| ***Within treatments differences*** | CS | | | | | | | |
|---|---|---|---|---|---|---|---|---|
| ***Time effect*** | Tukey | 0 | 2 | 5 | 7 | 9 | 12 | 14 |
| C | P < 0.001 | a | b | bc | c | c | c | c |
| -P | P < 0.001 | a | bc | cd | d | d | abc | ab |
| -Fe | P < 0.001 | a | b | bc | cd | d | cd | d |
| -P/-Fe | P < 0.001 | a | ab | bc | bc | c | abc | abc |

| ***Within treatments differences*** | MR | | | | | | | |
|---|---|---|---|---|---|---|---|---|
| ***Time effect*** | Tukey | 0 | 2 | 4 | 7 | 9 | 11 | 14 |
| C | P < 0.001 | a | b | b | c | d | d | d |
| -P | P < 0.001 | a | a | a | b | bc | bc | c |
| -Fe | P < 0.001 | a | ab | ab | bc | cd | c | d |
| -P/-Fe | P < 0.001 | a | b | bc | cd | d | d | e |

| ***Within treatments differences*** | MS | | | | | | | |
|---|---|---|---|---|---|---|---|---|
| ***Time effect*** | Tukey | 0 | 2 | 4 | 7 | 9 | 11 | 14 |
| C | P < 0.001 | a | b | bc | c | d | d | d |
| -P | P < 0.001 | a | b | bc | cd | e | de | e |
| -Fe | P < 0.001 | a | bc | b | c | cd | c | d |
| -P/-Fe | P < 0.001 | a | b | b | bc | c | c | c |

| ***Within treatments differences*** | TR | | | | | | | |
|---|---|---|---|---|---|---|---|---|
| ***Time effect*** | Tukey | 0 | 1 | 3 | 7 | 9 | 11 | 14 |
| C | P < 0.001 | a | b | c | c | d | d | d |
| -P | P < 0.001 | a | a | b | b | b | b | b |
| -Fe | P < 0.001 | a | b | c | d | de | e | e |
| -P/-Fe | P < 0.001 | a | a | b | b | b | b | b |

| ***Within treatments differences*** | TS | | | | | | | |
|---|---|---|---|---|---|---|---|---|
| ***Time effect*** | Tukey | 0 | 1 | 3 | 7 | 9 | 11 | 14 |
| C | P < 0.001 | a | b | cd | c | de | e | de |

| | | | | | | | | |
|---|---|---|---|---|---|---|---|---|
| -P | P < 0.001 | a | b | c | b | b | b | b |
| -Fe | P < 0.001 | a | b | bc | bc | cd | d | d |
| -P/-Fe | P < 0.001 | a | ab | c | c | c | c | bc |

**Table S4:** Table summarizing the statistical analyses performed on the fresh weight of the analysed plants shown in the article supplementary figure 2. The table is divided in six sections: summary table for number of replicates (N), mean fresh weight, standard deviation (sd) and standard error (se), Two-Way Anova, One-Way Anova on treatments, Tukey.HSD multiple comparison on treatments, One-Way Anova on time and Tukey.HSD multiple comparison on time. BR= Barley Root; BS=Barley Shoot; CR= Cucumber Root; CS=Cucumber Shoot; MR= Maize Root; MS=Maize Shoot; TS=Tomato Shoot; TR= Tomato Root; n.a. = not analysed.

***Summary table***

| Treatment | Time | Species_Tissue | N | Mean Fresh Weight (g) | sd | se |
|---|---|---|---|---|---|---|
| C | -17 | Maize_Root | 3 | 0.1153 | 0.0099 | 0.0057 |
| C | -17 | Maize_Shoot | 3 | 0.1153 | 0.0099 | 0.0057 |
| C | -14 | Tomato_Root | 3 | 0.0031 | 0.0007 | 0.0004 |
| C | -14 | Tomato_Shoot | 3 | 0.0031 | 0.0007 | 0.0004 |
| C | -13 | Cucumber_Root | 3 | 0.0274 | 0.0021 | 0.0012 |
| C | -13 | Cucumber_Shoot | 3 | 0.0274 | 0.0021 | 0.0012 |
| C | -11 | Barley_Root | 3 | 0.0429 | 0.0046 | 0.0027 |
| C | -11 | Barley_Shoot | 3 | 0.0429 | 0.0046 | 0.0027 |
| C | -7 | Barley_Root | 7 | 0.0762 | 0.0230 | 0.0087 |
| C | -7 | Barley_Shoot | 7 | 0.0574 | 0.0148 | 0.0056 |
| C | -7 | Cucumber_Root | 7 | 0.0544 | 0.0148 | 0.0056 |
| C | -7 | Cucumber_Shoot | 7 | 0.1124 | 0.0229 | 0.0087 |
| C | -7 | Maize_Root | 7 | 0.2839 | 0.1256 | 0.0475 |
| C | -7 | Maize_Shoot | 7 | 0.4660 | 0.1067 | 0.0403 |
| C | -7 | Tomato_Root | 7 | 0.0134 | 0.0045 | 0.0017 |
| C | -7 | Tomato_Shoot | 7 | 0.0219 | 0.0044 | 0.0017 |
| C | -3 | Barley_Root | 7 | 0.1895 | 0.0398 | 0.0150 |
| C | -3 | Barley_Shoot | 7 | 0.2494 | 0.0556 | 0.0210 |
| C | -3 | Maize_Root | 7 | 0.4842 | 0.1646 | 0.0622 |
| C | -3 | Maize_Shoot | 7 | 0.7369 | 0.2157 | 0.0815 |
| C | -3 | Tomato_Root | 7 | 0.0057 | 0.0017 | 0.0006 |
| C | -3 | Tomato_Shoot | 7 | 0.0607 | 0.0063 | 0.0024 |
| C | -2 | Cucumber_Root | 7 | 0.1710 | 0.0190 | 0.0072 |
| C | -2 | Cucumber_Shoot | 7 | 0.7243 | 0.0786 | 0.0297 |
| C | 0 | Barley_Root | 7 | 0.1953 | 0.0336 | 0.0127 |
| C | 0 | Barley_Shoot | 7 | 0.4287 | 0.0729 | 0.0275 |
| C | 0 | Cucumber_Root | 7 | 0.2527 | 0.1206 | 0.0456 |
| C | 0 | Cucumber_Shoot | 7 | 0.9317 | 0.3152 | 0.1191 |
| C | 0 | Maize_Root | 7 | 0.5123 | 0.2019 | 0.0763 |
| C | 0 | Maize_Shoot | 7 | 1.1437 | 0.4252 | 0.1607 |
| C | 0 | Tomato_Root | 7 | 0.0123 | 0.0056 | 0.0021 |
| C | 0 | Tomato_Shoot | 7 | 0.0978 | 0.0318 | 0.0120 |
| C | 1 | Tomato_Root | 7 | 0.0142 | 0.0053 | 0.0020 |
| C | 1 | Tomato_Shoot | 7 | 0.1379 | 0.0321 | 0.0121 |
| C | 2 | Barley_Root | 7 | 0.1883 | 0.0513 | 0.0194 |
| C | 2 | Barley_Shoot | 7 | 0.4438 | 0.1080 | 0.0408 |
| C | 2 | Cucumber_Root | 7 | 0.4390 | 0.1061 | 0.0401 |

| | | | | | | |
|---|---|---|---|---|---|---|
| C | 2 | Cucumber_Shoot | 7 | 1.2222 | 0.2375 | 0.0898 |
| C | 2 | Maize_Root | 7 | 0.5745 | 0.2138 | 0.0808 |
| C | 2 | Maize_Shoot | 7 | 1.4887 | 0.8379 | 0.3167 |
| C | 3 | Tomato_Root | 7 | 0.0277 | 0.0086 | 0.0033 |
| C | 3 | Tomato_Shoot | 7 | 0.2277 | 0.0263 | 0.0099 |
| C | 4 | Barley_Root | 7 | 0.2401 | 0.0631 | 0.0238 |
| C | 4 | Barley_Shoot | 7 | 0.6244 | 0.1102 | 0.0416 |
| C | 4 | Maize_Root | 7 | 0.6053 | 0.2211 | 0.0836 |
| C | 4 | Maize_Shoot | 7 | 1.8079 | 0.8255 | 0.3120 |
| C | 5 | Cucumber_Root | 7 | 0.8331 | 0.4084 | 0.1544 |
| C | 5 | Cucumber_Shoot | 7 | 1.9610 | 0.6204 | 0.2345 |
| C | 7 | Barley_Root | 7 | 0.2745 | 0.1084 | 0.0410 |
| C | 7 | Barley_Shoot | 7 | 0.9324 | 0.2046 | 0.0773 |
| C | 7 | Cucumber_Root | 7 | 1.4161 | 0.7952 | 0.3005 |
| C | 7 | Cucumber_Shoot | 7 | 2.6404 | 0.3206 | 0.1212 |
| C | 7 | Maize_Root | 7 | 0.7213 | 0.2570 | 0.0971 |
| C | 7 | Maize_Shoot | 7 | 2.5310 | 1.2165 | 0.4598 |
| C | 7 | Tomato_Root | 7 | 0.0829 | 0.0257 | 0.0097 |
| C | 7 | Tomato_Shoot | 7 | 0.6053 | 0.1419 | 0.0536 |
| C | 9 | Barley_Root | 7 | 0.3642 | 0.0745 | 0.0282 |
| C | 9 | Barley_Shoot | 7 | 1.1306 | 0.1541 | 0.0583 |
| C | 9 | Cucumber_Root | 7 | 1.0738 | 0.9012 | 0.3406 |
| C | 9 | Cucumber_Shoot | 7 | 2.8581 | 1.1137 | 0.4209 |
| C | 9 | Maize_Root | 7 | 0.9129 | 0.3424 | 0.1294 |
| C | 9 | Maize_Shoot | 7 | 3.0429 | 1.1656 | 0.4405 |
| C | 9 | Tomato_Root | 7 | 0.1343 | 0.0410 | 0.0155 |
| C | 9 | Tomato_Shoot | 7 | 1.0111 | 0.2573 | 0.0972 |
| C | 11 | Barley_Root | 7 | 0.4164 | 0.1093 | 0.0413 |
| C | 11 | Barley_Shoot | 7 | 1.4425 | 0.1676 | 0.0634 |
| C | 11 | Maize_Root | 7 | 1.0307 | 0.3796 | 0.1435 |
| C | 11 | Maize_Shoot | 7 | 4.0139 | 1.6422 | 0.6207 |
| C | 11 | Tomato_Root | 7 | 0.2806 | 0.0478 | 0.0181 |
| C | 11 | Tomato_Shoot | 7 | 1.9183 | 0.2647 | 0.1000 |
| C | 12 | Cucumber_Root | 7 | 2.1861 | 0.9605 | 0.3630 |
| C | 12 | Cucumber_Shoot | 7 | 5.7880 | 1.4084 | 0.5323 |
| C | 14 | Barley_Root | 7 | 0.6195 | 0.0987 | 0.0373 |
| C | 14 | Barley_Shoot | 7 | 1.8219 | 0.2685 | 0.1015 |
| C | 14 | Cucumber_Root | 7 | 2.1696 | 1.1640 | 0.4400 |
| C | 14 | Cucumber_Shoot | 7 | 5.2629 | 1.2051 | 0.4555 |
| C | 14 | Maize_Root | 7 | 1.2642 | 0.5732 | 0.2166 |
| C | 14 | Maize_Shoot | 7 | 7.6429 | 2.7924 | 1.0554 |
| C | 14 | Tomato_Root | 7 | 0.4606 | 0.2035 | 0.0769 |
| C | 14 | Tomato_Shoot | 7 | 2.7797 | 0.9343 | 0.3531 |
| Fe | 0 | Barley_Root | 7 | 0.1874 | 0.0611 | 0.0231 |
| Fe | 0 | Barley_Shoot | 7 | 0.3945 | 0.0518 | 0.0196 |
| Fe | 0 | Cucumber_Root | 7 | 0.2439 | 0.1073 | 0.0406 |
| Fe | 0 | Cucumber_Shoot | 7 | 0.8127 | 0.1176 | 0.0444 |
| Fe | 0 | Maize_Root | 7 | 0.2550 | 0.1025 | 0.0387 |
| Fe | 0 | Maize_Shoot | 7 | 0.5913 | 0.2930 | 0.1107 |
| Fe | 0 | Tomato_Root | 7 | 0.0165 | 0.0039 | 0.0015 |

| | | | | | | |
|---|---|---|---|---|---|---|
| Fe | 0 | Tomato_Shoot | 7 | 0.1276 | 0.0439 | 0.0166 |
| Fe | 1 | Tomato_Root | 7 | 0.0178 | 0.0061 | 0.0023 |
| Fe | 1 | Tomato_Shoot | 7 | 0.1507 | 0.0269 | 0.0102 |
| Fe | 2 | Barley_Root | 7 | 0.1968 | 0.0461 | 0.0174 |
| Fe | 2 | Barley_Shoot | 7 | 0.4751 | 0.1064 | 0.0402 |
| Fe | 2 | Cucumber_Root | 7 | 0.5024 | 0.1454 | 0.0549 |
| Fe | 2 | Cucumber_Shoot | 7 | 1.3431 | 0.2069 | 0.0782 |
| Fe | 2 | Maize_Root | 7 | 0.5677 | 0.1885 | 0.0712 |
| Fe | 2 | Maize_Shoot | 7 | 1.5896 | 0.4720 | 0.1784 |
| Fe | 3 | Tomato_Root | 7 | 0.0345 | 0.0056 | 0.0021 |
| Fe | 3 | Tomato_Shoot | 7 | 0.2286 | 0.0252 | 0.0095 |
| Fe | 4 | Barley_Root | 7 | 0.2401 | 0.0568 | 0.0215 |
| Fe | 4 | Barley_Shoot | 7 | 0.6552 | 0.1162 | 0.0439 |
| Fe | 4 | Maize_Root | 7 | 0.7680 | 0.3468 | 0.1311 |
| Fe | 4 | Maize_Shoot | 7 | 1.9396 | 0.6463 | 0.2443 |
| Fe | 5 | Cucumber_Root | 7 | 0.5684 | 0.1480 | 0.0559 |
| Fe | 5 | Cucumber_Shoot | 7 | 2.0342 | 0.3136 | 0.1185 |
| Fe | 7 | Barley_Root | 7 | 0.3348 | 0.0978 | 0.0370 |
| Fe | 7 | Barley_Shoot | 7 | 0.8996 | 0.2342 | 0.0885 |
| Fe | 7 | Cucumber_Root | 7 | 0.7192 | 0.2685 | 0.1015 |
| Fe | 7 | Cucumber_Shoot | 7 | 2.0936 | 0.3185 | 0.1204 |
| Fe | 7 | Maize_Root | 7 | 0.7187 | 0.2529 | 0.0956 |
| Fe | 7 | Maize_Shoot | 7 | 2.5165 | 0.9218 | 0.3484 |
| Fe | 7 | Tomato_Root | 7 | 0.0941 | 0.0126 | 0.0048 |
| Fe | 7 | Tomato_Shoot | 7 | 0.4453 | 0.0565 | 0.0214 |
| Fe | 9 | Barley_Root | 7 | 0.4675 | 0.2579 | 0.0975 |
| Fe | 9 | Barley_Shoot | 7 | 1.1426 | 0.3635 | 0.1374 |
| Fe | 9 | Cucumber_Root | 7 | 1.1553 | 0.9846 | 0.3722 |
| Fe | 9 | Cucumber_Shoot | 7 | 2.9146 | 0.9885 | 0.3736 |
| Fe | 9 | Maize_Root | 7 | 1.0590 | 0.3702 | 0.1399 |
| Fe | 9 | Maize_Shoot | 7 | 3.2452 | 1.0380 | 0.3923 |
| Fe | 9 | Tomato_Root | 7 | 0.1304 | 0.0379 | 0.0143 |
| Fe | 9 | Tomato_Shoot | 7 | 0.5668 | 0.0971 | 0.0367 |
| Fe | 11 | Barley_Root | 7 | 0.3560 | 0.1058 | 0.0400 |
| Fe | 11 | Barley_Shoot | 7 | 1.1784 | 0.2101 | 0.0794 |
| Fe | 11 | Maize_Root | 7 | 1.4036 | 0.6023 | 0.2277 |
| Fe | 11 | Maize_Shoot | 7 | 4.0944 | 0.9380 | 0.3545 |
| Fe | 11 | Tomato_Root | 7 | 0.2239 | 0.1067 | 0.0403 |
| Fe | 11 | Tomato_Shoot | 7 | 0.8226 | 0.4149 | 0.1568 |
| Fe | 12 | Cucumber_Root | 7 | 1.2211 | 0.8133 | 0.3074 |
| Fe | 12 | Cucumber_Shoot | 7 | 4.1011 | 1.1232 | 0.4245 |
| Fe | 14 | Barley_Root | 7 | 0.7258 | 0.2391 | 0.0904 |
| Fe | 14 | Barley_Shoot | 7 | 1.7478 | 0.3666 | 0.1386 |
| Fe | 14 | Cucumber_Root | 7 | 2.0879 | 2.1400 | 0.8088 |
| Fe | 14 | Cucumber_Shoot | 7 | 5.4237 | 2.2879 | 0.8647 |
| Fe | 14 | Maize_Root | 7 | 1.7211 | 0.4661 | 0.1762 |
| Fe | 14 | Maize_Shoot | 7 | 5.6070 | 1.3148 | 0.4970 |
| Fe | 14 | Tomato_Root | 7 | 0.2997 | 0.1375 | 0.0520 |
| Fe | 14 | Tomato_Shoot | 7 | 0.9473 | 0.3485 | 0.1317 |
| P | 0 | Barley_Root | 7 | 0.1509 | 0.0484 | 0.0183 |

| | | | | | | |
|---|---|---|---|---|---|---|
| P | 0 | Barley_Shoot | 7 | 0.3085 | 0.0721 | 0.0272 |
| P | 0 | Cucumber_Root | 7 | 0.2712 | 0.0712 | 0.0269 |
| P | 0 | Cucumber_Shoot | 7 | 0.8780 | 0.1417 | 0.0536 |
| P | 0 | Maize_Root | 7 | 0.3803 | 0.1647 | 0.0623 |
| P | 0 | Maize_Shoot | 7 | 0.6988 | 0.1237 | 0.0467 |
| P | 0 | Tomato_Root | 7 | 0.0166 | 0.0103 | 0.0039 |
| P | 0 | Tomato_Shoot | 7 | 0.0908 | 0.0403 | 0.0152 |
| P | 1 | Tomato_Root | 7 | 0.0142 | 0.0021 | 0.0008 |
| P | 1 | Tomato_Shoot | 7 | 0.1468 | 0.0321 | 0.0121 |
| P | 2 | Barley_Root | 7 | 0.2377 | 0.0560 | 0.0212 |
| P | 2 | Barley_Shoot | 7 | 0.4851 | 0.0898 | 0.0339 |
| P | 2 | Cucumber_Root | 7 | 0.4217 | 0.1676 | 0.0633 |
| P | 2 | Cucumber_Shoot | 7 | 1.1824 | 0.4252 | 0.1607 |
| P | 2 | Maize_Root | 7 | 0.6231 | 0.1209 | 0.0457 |
| P | 2 | Maize_Shoot | 7 | 1.6603 | 0.4099 | 0.1549 |
| P | 3 | Tomato_Root | 7 | 0.0471 | 0.0116 | 0.0044 |
| P | 3 | Tomato_Shoot | 7 | 0.2470 | 0.0406 | 0.0153 |
| P | 4 | Barley_Root | 7 | 0.2876 | 0.0675 | 0.0255 |
| P | 4 | Barley_Shoot | 7 | 0.6116 | 0.1103 | 0.0417 |
| P | 4 | Maize_Root | 7 | 0.6997 | 0.3112 | 0.1176 |
| P | 4 | Maize_Shoot | 7 | 2.1214 | 1.1355 | 0.4292 |
| P | 5 | Cucumber_Root | 7 | 0.7620 | 0.4177 | 0.1579 |
| P | 5 | Cucumber_Shoot | 7 | 1.7643 | 0.4761 | 0.1799 |
| P | 7 | Barley_Root | 7 | 0.4194 | 0.1106 | 0.0418 |
| P | 7 | Barley_Shoot | 7 | 0.9065 | 0.1785 | 0.0675 |
| P | 7 | Cucumber_Root | 7 | 0.9605 | 0.4976 | 0.1881 |
| P | 7 | Cucumber_Shoot | 7 | 2.1313 | 0.4271 | 0.1614 |
| P | 7 | Maize_Root | 7 | 0.8121 | 0.4245 | 0.1605 |
| P | 7 | Maize_Shoot | 7 | 2.4737 | 1.1166 | 0.4220 |
| P | 7 | Tomato_Root | 7 | 0.0909 | 0.0399 | 0.0151 |
| P | 7 | Tomato_Shoot | 7 | 0.3522 | 0.0548 | 0.0207 |
| P | 9 | Barley_Root | 7 | 0.5093 | 0.1757 | 0.0664 |
| P | 9 | Barley_Shoot | 7 | 1.2273 | 0.2196 | 0.0830 |
| P | 9 | Cucumber_Root | 7 | 0.9319 | 0.5583 | 0.2110 |
| P | 9 | Cucumber_Shoot | 7 | 2.4251 | 0.2261 | 0.0854 |
| P | 9 | Maize_Root | 7 | 0.8504 | 0.4089 | 0.1546 |
| P | 9 | Maize_Shoot | 7 | 2.7570 | 1.5058 | 0.5691 |
| P | 9 | Tomato_Root | 7 | 0.1818 | 0.0552 | 0.0209 |
| P | 9 | Tomato_Shoot | 7 | 0.4991 | 0.1179 | 0.0446 |
| P | 11 | Barley_Root | 7 | 0.5078 | 0.1199 | 0.0453 |
| P | 11 | Barley_Shoot | 7 | 1.2420 | 0.2486 | 0.0940 |
| P | 11 | Maize_Root | 7 | 1.0476 | 0.5241 | 0.1981 |
| P | 11 | Maize_Shoot | 7 | 3.9206 | 1.8813 | 0.7111 |
| P | 11 | Tomato_Root | 7 | 0.1917 | 0.0748 | 0.0283 |
| P | 11 | Tomato_Shoot | 7 | 0.5253 | 0.1072 | 0.0405 |
| P | 12 | Cucumber_Root | 7 | 2.4401 | 1.2913 | 0.4881 |
| P | 12 | Cucumber_Shoot | 7 | 2.9252 | 0.3125 | 0.1181 |
| P | 14 | Barley_Root | 7 | 1.0626 | 0.2966 | 0.1121 |
| P | 14 | Barley_Shoot | 7 | 1.7805 | 0.3369 | 0.1274 |
| P | 14 | Cucumber_Root | 7 | 2.2000 | 1.2495 | 0.4723 |

| | | | | | | |
|---|---|---|---|---|---|---|
| P | 14 | Cucumber_Shoot | 7 | 3.7858 | 0.9956 | 0.3763 |
| P | 14 | Maize_Root | 7 | 1.8737 | 0.8442 | 0.3191 |
| P | 14 | Maize_Shoot | 7 | 7.0365 | 2.4266 | 0.9172 |
| P | 14 | Tomato_Root | 7 | 0.3012 | 0.0520 | 0.0197 |
| P | 14 | Tomato_Shoot | 7 | 0.5255 | 0.0855 | 0.0323 |
| P/Fe | 0 | Barley_Root | 7 | 0.1631 | 0.0294 | 0.0111 |
| P/Fe | 0 | Barley_Shoot | 7 | 0.3474 | 0.0773 | 0.0292 |
| P/Fe | 0 | Cucumber_Root | 7 | 0.3324 | 0.0705 | 0.0267 |
| P/Fe | 0 | Cucumber_Shoot | 7 | 1.0026 | 0.0989 | 0.0374 |
| P/Fe | 0 | Maize_Root | 7 | 0.3754 | 0.1284 | 0.0485 |
| P/Fe | 0 | Maize_Shoot | 7 | 0.5603 | 0.3007 | 0.1137 |
| P/Fe | 0 | Tomato_Root | 7 | 0.0126 | 0.0056 | 0.0021 |
| P/Fe | 0 | Tomato_Shoot | 7 | 0.1054 | 0.0192 | 0.0073 |
| P/Fe | 1 | Tomato_Root | 7 | 0.0121 | 0.0057 | 0.0021 |
| P/Fe | 1 | Tomato_Shoot | 7 | 0.1086 | 0.0366 | 0.0138 |
| P/Fe | 2 | Barley_Root | 7 | 0.2364 | 0.0325 | 0.0123 |
| P/Fe | 2 | Barley_Shoot | 7 | 0.5342 | 0.0652 | 0.0246 |
| P/Fe | 2 | Cucumber_Root | 7 | 0.4462 | 0.1201 | 0.0454 |
| P/Fe | 2 | Cucumber_Shoot | 7 | 1.2273 | 0.1784 | 0.0674 |
| P/Fe | 2 | Maize_Root | 7 | 0.6182 | 0.2240 | 0.0847 |
| P/Fe | 2 | Maize_Shoot | 7 | 1.4593 | 0.6769 | 0.2558 |
| P/Fe | 3 | Tomato_Root | 7 | 0.0362 | 0.0085 | 0.0032 |
| P/Fe | 3 | Tomato_Shoot | 7 | 0.2009 | 0.0417 | 0.0158 |
| P/Fe | 4 | Barley_Root | 7 | 0.3019 | 0.1031 | 0.0390 |
| P/Fe | 4 | Barley_Shoot | 7 | 0.6439 | 0.1787 | 0.0675 |
| P/Fe | 4 | Maize_Root | 7 | 0.6515 | 0.2347 | 0.0887 |
| P/Fe | 4 | Maize_Shoot | 7 | 1.7507 | 0.6433 | 0.2431 |
| P/Fe | 5 | Cucumber_Root | 7 | 0.8496 | 0.4157 | 0.1571 |
| P/Fe | 5 | Cucumber_Shoot | 7 | 1.6486 | 0.3795 | 0.1434 |
| P/Fe | 7 | Barley_Root | 7 | 0.3836 | 0.0964 | 0.0365 |
| P/Fe | 7 | Barley_Shoot | 7 | 0.8876 | 0.1693 | 0.0640 |
| P/Fe | 7 | Cucumber_Root | 7 | 0.8493 | 0.3551 | 0.1342 |
| P/Fe | 7 | Cucumber_Shoot | 7 | 1.9223 | 0.4556 | 0.1722 |
| P/Fe | 7 | Maize_Root | 7 | 0.6520 | 0.4150 | 0.1569 |
| P/Fe | 7 | Maize_Shoot | 7 | 1.7085 | 1.1923 | 0.4507 |
| P/Fe | 7 | Tomato_Root | 7 | 0.0913 | 0.0213 | 0.0080 |
| P/Fe | 7 | Tomato_Shoot | 7 | 0.2984 | 0.0516 | 0.0195 |
| P/Fe | 9 | Barley_Root | 7 | 0.4507 | 0.1759 | 0.0665 |
| P/Fe | 9 | Barley_Shoot | 7 | 0.9850 | 0.1365 | 0.0516 |
| P/Fe | 9 | Cucumber_Root | 7 | 1.1223 | 0.7092 | 0.2680 |
| P/Fe | 9 | Cucumber_Shoot | 7 | 1.7841 | 0.4263 | 0.1611 |
| P/Fe | 9 | Maize_Root | 7 | 1.0581 | 0.2992 | 0.1131 |
| P/Fe | 9 | Maize_Shoot | 7 | 2.8140 | 0.9364 | 0.3539 |
| P/Fe | 9 | Tomato_Root | 7 | 0.1175 | 0.0347 | 0.0131 |
| P/Fe | 9 | Tomato_Shoot | 7 | 0.3062 | 0.0798 | 0.0301 |
| P/Fe | 11 | Barley_Root | 7 | 0.5882 | 0.1627 | 0.0615 |
| P/Fe | 11 | Barley_Shoot | 7 | 1.3229 | 0.2396 | 0.0906 |
| P/Fe | 11 | Maize_Root | 7 | 1.2345 | 0.6576 | 0.2485 |
| P/Fe | 11 | Maize_Shoot | 7 | 3.6072 | 1.7651 | 0.6671 |
| P/Fe | 11 | Tomato_Root | 7 | 0.2018 | 0.0490 | 0.0185 |

| | | | | | | |
|---|---|---|---|---|---|---|
| P/Fe | 11 | Tomato_Shoot | 7 | 0.4021 | 0.0848 | 0.0320 |
| P/Fe | 12 | Cucumber_Root | 7 | 1.1486 | 0.6404 | 0.2421 |
| P/Fe | 12 | Cucumber_Shoot | 7 | 2.9083 | 0.8088 | 0.3057 |
| P/Fe | 14 | Barley_Root | 7 | 0.8982 | 0.1558 | 0.0589 |
| P/Fe | 14 | Barley_Shoot | 7 | 1.5134 | 0.2883 | 0.1090 |
| P/Fe | 14 | Cucumber_Root | 7 | 2.1477 | 0.7192 | 0.2718 |
| P/Fe | 14 | Cucumber_Shoot | 7 | 3.2689 | 0.4483 | 0.1694 |
| P/Fe | 14 | Maize_Root | 7 | 2.3029 | 0.4774 | 0.1804 |
| P/Fe | 14 | Maize_Shoot | 7 | 6.2228 | 1.2083 | 0.4567 |
| P/Fe | 14 | Tomato_Root | 7 | 0.2318 | 0.0850 | 0.0321 |
| P/Fe | 14 | Tomato_Shoot | 7 | 0.3337 | 0.1188 | 0.0449 |

**Two-Way ANOVA**

***$Barley_Root***

Analysis of Variance Table

Response: Value

| | Df | Sum Sq | Mean Sq | F value | Pr(>F) | |
|---|---|---|---|---|---|---|
| Treatment | 3 | 1.1453 | 0.38177 | 25.096 | 1.23E-13 | *** |
| Time | 9 | 8.8455 | 0.98284 | 64.606 | <2.2E-16 | *** |
| Treatment:Time | 18 | 0.7043 | 0.03913 | 2.572 | 0.000798 | *** |
| Residuals | 182 | 2.7687 | 0.01521 | | | |

\---

Signif. codes: 0 ‘***’ 0.001 ‘**’ 0.01 ‘*’ 0.05 ‘.’ 0.1 ‘’ 1

***$Barley_Shoot***

Analysis of Variance Table

Response: Value

| | Df | Sum Sq | Mean Sq | F value | Pr(>F) | |
|---|---|---|---|---|---|---|
| Treatment | 3 | 1.229 | 0.4097 | 11.2758 | 8.06E-07 | *** |
| Time | 9 | 47.979 | 5.331 | 146.7268 | <2.2E-16 | *** |
| Treatment:Time | 18 | 0.814 | 0.0452 | 1.2447 | 0.2301 | |
| Residuals | 182 | 6.613 | 0.0363 | | | |

\---

Signif. codes: 0 ‘***’ 0.001 ‘**’ 0.01 ‘*’ 0.05 ‘.’ 0.1 ‘’ 1

***$Cucumber_Root***

Analysis of Variance Table

Response: Value

| | Df | Sum Sq | Mean Sq | F value | Pr(>F) | |
|---|---|---|---|---|---|---|
| Treatment | 3 | 1.693 | 0.5644 | 1.0703 | 0.363 | |
| Time | 9 | 92.303 | 10.2559 | 19.4481 | <2.2E-16 | *** |
| Treatment:Time | 18 | 9.394 | 0.5219 | 0.9897 | 0.4736 | |
| Residuals | 182 | 95.977 | 0.5273 | | | |

\---

Signif. codes: 0 ‘***’ 0.001 ‘**’ 0.01 ‘*’ 0.05 ‘.’ 0.1 ‘’ 1

***$Cucumber_Shoot***

Analysis of Variance Table

Response: Value

| | Df | Sum Sq | Mean Sq | F value | Pr(>F) | |
|---|---|---|---|---|---|---|
| Treatment | 3 | 13.2 | 4.4 | 8.2202 | 3.69E-05 | *** |
| Time | 9 | 378.23 | 42.026 | 78.5164 | <2.2E-16 | *** |
| Treatment:Time | 18 | 40.98 | 2.277 | 4.2535 | 1.68E-07 | *** |
| Residuals | 182 | 97.42 | 0.535 | | | |

---

| | | | | | | |
|---|---|---|---|---|---|---|
| Signif. | codes: | 0 | ‘***’ | 0.001 | ‘**’ | 0.01 ‘*’ |
| | | 0.05 | ‘.’ | 0.1 | ‘’ | 1 |

***$Maize_Root***

Analysis of Variance Table

Response: Value

| | Df | Sum Sq | Mean Sq | F value | Pr(>F) | |
|---|---|---|---|---|---|---|
| Treatment | 3 | 3.126 | 1.0421 | 7.3533 | 0.000112 | *** |
| Time | 9 | 39.912 | 4.4346 | 31.2924 | <2.2E-16 | *** |
| Treatment:Time | 18 | 4.336 | 0.2409 | 1.6999 | 0.042615 | * |
| Residuals | 182 | 25.792 | 0.1417 | | | |

---

| | | | | | | |
|---|---|---|---|---|---|---|
| Signif. | codes: | 0 | ‘***’ | 0.001 | ‘**’ | 0.01 ‘*’ |
| | | 0.05 | ‘.’ | 0.1 | ‘’ | 1 |

***$Maize_Shoot***

Analysis of Variance Table

Response: Value

| | Df | Sum Sq | Mean Sq | F value | Pr(>F) | |
|---|---|---|---|---|---|---|
| Treatment | 3 | 8.8 | 2.932 | 2.094 | 0.1026 | |
| Time | 9 | 720.39 | 80.043 | 57.1725 | <2.2E-16 | *** |
| Treatment:Time | 18 | 17.54 | 0.975 | 0.6961 | 0.8124 | |
| Residuals | 182 | 254.81 | 1.4 | | | |

---

| | | | | | | |
|---|---|---|---|---|---|---|
| Signif. | codes: | 0 | ‘***’ | 0.001 | ‘**’ | 0.01 ‘*’ |
| | | 0.05 | ‘.’ | 0.1 | ‘’ | 1 |

***$Tomato_Root***

Analysis of Variance Table

Response: Value

| | Df | Sum Sq | Mean Sq | F value | Pr(>F) | |
|---|---|---|---|---|---|---|
| Treatment | 3 | 0.01146 | 0.003819 | 1.1271 | 0.3394 | |
| Time | 9 | 2.55408 | 0.283786 | 83.7575 | <2.2E-16 | *** |
| Treatment:Time | 18 | 0.20086 | 0.011159 | 3.2935 | 2.20E-05 | *** |
| Residuals | 182 | 0.61665 | 0.003388 | | | |

---

| | | | | | | |
|---|---|---|---|---|---|---|
| Signif. | codes: | 0 | ‘***’ | 0.001 | ‘**’ | 0.01 ‘*’ |
| | | 0.05 | ‘.’ | 0.1 | ‘’ | 1 |

***$Tomato_Shoot***

Analysis of Variance Table

Response: Value

| | Df | Sum Sq | Mean Sq | F value | Pr(>F) | |
|---|---|---|---|---|---|---|
| Treatment | 3 | 7.557 | 2.5192 | 53.993 | <2.2E-16 | *** |
| Time | 9 | 38.162 | 4.2402 | 90.88 | <2.2E-16 | *** |

| | | | | | | |
|---|---|---|---|---|---|---|
| Treatment:Time | 18 | 23.489 | 1.3049 | 27.969 | <2.2E-16 | *** |
| Residuals | 182 | 8.492 | 0.0467 | | | |

---

| | | | | | | | |
|---|---|---|---|---|---|---|---|
| Signif. | codes: | 0 | ‘***’ | 0.001 | ‘**’ | 0.01 | ‘*’ |
| | | 0.05 | ‘.’ | 0.1 | ‘’ | 1 | |

**One-Way ANOVA on treatments**

***$Barley_Root***

$Barley_Root$`0`

Analysis of Variance Table

Response: Value

| | Df | Sum Sq | Mean Sq | F value | Pr(>F) |
|---|---|---|---|---|---|
| Treatment | 3 | 0.008983 | 0.002994 | 1.4836 | 0.2441 |
| Residuals | 24 | 0.048437 | 0.002018 | | |

$Barley_Root$`2`

Analysis of Variance Table

Response: Value

| | Df | Sum Sq | Mean Sq | F value | Pr(>F) |
|---|---|---|---|---|---|
| Treatment | 3 | 0.014087 | 0.004696 | 2.0976 | 0.1271 |
| Residuals | 24 | 0.053726 | 0.002239 | | |

$Barley_Root$`4`

Analysis of Variance Table

Response: Value

| | Df | Sum Sq | Mean Sq | F value | Pr(>F) |
|---|---|---|---|---|---|
| Treatment | 3 | 0.021629 | 0.00721 | 1.2889 | 0.3009 |
| Residuals | 24 | 0.134248 | 0.005594 | | |

$Barley_Root$`7`

Analysis of Variance Table

Response: Value

| | Df | Sum Sq | Mean Sq | F value | Pr(>F) | |
|---|---|---|---|---|---|---|
| Treatment | 3 | 0.082853 | 0.027618 | 2.5787 | 0.07719 | . |
| Residuals | 24 | 0.257043 | 0.01071 | | | |

---

Signif. codes: 0 ‘***’ 0.001 ‘**’ 0.01 ‘*’ 0.05 ‘.’ 0.1 ‘’ 1

$Barley_Root$`9`

Analysis of Variance Table

Response: Value

| | Df | Sum Sq | Mean Sq | F value | Pr(>F) |
|---|---|---|---|---|---|
| Treatment | 3 | 0.07811 | 0.026037 | 0.7777 | 0.5179 |
| Residuals | 24 | 0.80348 | 0.033478 | | |

$Barley_Root$`11`

Analysis of Variance Table

Response: Value

| | Df | Sum Sq | Mean Sq | F value | Pr(>F) | |
|---|---|---|---|---|---|---|
| Treatment | 3 | 0.21874 | 0.072913 | 4.5584 | 0.01153 | * |
| Residuals | 24 | 0.38389 | 0.015995 | | | |

---
Signif. codes: 0 ‘***’ 0.001 ‘**’ 0.01 ‘*’ 0.05 ‘.’ 0.1 ‘’ 1

$Barley_Root$`14`
Analysis of Variance Table

Response: Value

| | Df | Sum Sq | Mean Sq | F value | Pr(>F) | |
|---|---|---|---|---|---|---|
| Treatment | 3 | 0.79715 | 0.265718 | 5.9315 | 0.003548 | ** |
| Residuals | 24 | 1.07515 | 0.044798 | | | |

---
Signif. codes: 0 ‘***’ 0.001 ‘**’ 0.01 ‘*’ 0.05 ‘.’ 0.1 ‘’ 1

***$Barley_Shoot***

$Barley_Shoot$`0`
Analysis of Variance Table

Response: Value

| | Df | Sum Sq | Mean Sq | F value | Pr(>F) | |
|---|---|---|---|---|---|---|
| Treatment | 3 | 0.058354 | 0.019451 | 4.0602 | 0.01818 | * |
| Residuals | 24 | 0.114976 | 0.004791 | | | |

---
Signif. codes: 0 ‘***’ 0.001 ‘**’ 0.01 ‘*’ 0.05 ‘.’ 0.1 ‘’ 1

$Barley_Shoot$`2`
Analysis of Variance Table

Response: Value

| | Df | Sum Sq | Mean Sq | F value | Pr(>F) |
|---|---|---|---|---|---|
| Treatment | 3 | 0.029487 | 0.009829 | 1.114 | 0.3629 |
| Residuals | 24 | 0.211761 | 0.008823 | | |

$Barley_Shoot$`4`
Analysis of Variance Table

Response: Value

| | Df | Sum Sq | Mean Sq | F value | Pr(>F) |
|---|---|---|---|---|---|
| Treatment | 3 | 0.00799 | 0.002662 | 0.1527 | 0.9269 |
| Residuals | 24 | 0.41844 | 0.017435 | | |

$Barley_Shoot$`7`
Analysis of Variance Table

Response: Value

| | Df | Sum Sq | Mean Sq | F value | Pr(>F) |
|---|---|---|---|---|---|
| Treatment | 3 | 0.00754 | 0.002514 | 0.0639 | 0.9784 |

| | | | | | |
|---|---|---|---|---|---|
| Residuals | 24 | 0.94341 | 0.039309 | | |

$Barley_Shoot$`9`
Analysis of Variance Table

Response: Value

| | Df | Sum Sq | Mean Sq | F value | Pr(>F) |
|---|---|---|---|---|---|
| Treatment | 3 | 0.21235 | 0.070782 | 1.2712 | 0.3066 |
| Residuals | 24 | 1.3363 | 0.055679 | | |

$Barley_Shoot$`11`
Analysis of Variance Table

Response: Value

| | Df | Sum Sq | Mean Sq | F value | Pr(>F) |
|---|---|---|---|---|---|
| Treatment | 3 | 0.27251 | 0.090837 | 1.8976 | 0.157 |
| Residuals | 24 | 1.14887 | 0.04787 | | |

$Barley_Shoot$`14`
Analysis of Variance Table

Response: Value

| | Df | Sum Sq | Mean Sq | F value | Pr(>F) |
|---|---|---|---|---|---|
| Treatment | 3 | 0.40223 | 0.13408 | 1.3303 | 0.2878 |
| Residuals | 24 | 2.41893 | 0.10079 | | |

***$Cucumber_Root***

$Cucumber_Root$`0`
Analysis of Variance Table

Response: Value

| | Df | Sum Sq | Mean Sq | F value | Pr(>F) |
|---|---|---|---|---|---|
| Treatment | 3 | 0.033371 | 0.011124 | 1.2323 | 0.3197 |
| Residuals | 24 | 0.216636 | 0.009027 | | |

$Cucumber_Root$`2`
Analysis of Variance Table

Response: Value

| | Df | Sum Sq | Mean Sq | F value | Pr(>F) |
|---|---|---|---|---|---|
| Treatment | 3 | 0.02564 | 0.008546 | 0.4565 | 0.7152 |
| Residuals | 24 | 0.44931 | 0.018721 | | |

$Cucumber_Root$`5`
Analysis of Variance Table

Response: Value

| | Df | Sum Sq | Mean Sq | F value | Pr(>F) |
|---|---|---|---|---|---|

| | | | | | | |
|---|---|---|---|---|---|---|
| Treatment | 3 | 0.3492 | 0.11641 | 0.8687 | 0.4709 | |
| Residuals | 24 | 3.2161 | 0.134 | | | |

$Cucumber_Root$`7`

Analysis of Variance Table

Response: Value

| | Df | Sum Sq | Mean Sq | F value | Pr(>F) | |
|---|---|---|---|---|---|---|
| Treatment | 3 | 1.9282 | 0.64274 | 2.3849 | 0.09421 | . |
| Residuals | 24 | 6.468 | 0.2695 | | | |

---

Signif. codes: 0 ‘***’ 0.001 ‘**’ 0.01 ‘*’ 0.05 ‘.’ 0.1 ‘’ 1

$Cucumber_Root$`9`

Analysis of Variance Table

Response: Value

| | Df | Sum Sq | Mean Sq | F value | Pr(>F) |
|---|---|---|---|---|---|
| Treatment | 3 | 0.2037 | 0.06789 | 0.1046 | 0.9566 |
| Residuals | 24 | 15.5774 | 0.64906 | | |

$Cucumber_Root$`12`

Analysis of Variance Table

Response: Value

| | Df | Sum Sq | Mean Sq | F value | Pr(>F) | |
|---|---|---|---|---|---|---|
| Treatment | 3 | 9.1547 | 3.05157 | 3.3337 | 0.0363 | * |
| Residuals | 24 | 21.9691 | 0.91538 | | | |

---

Signif. codes: 0 ‘***’ 0.001 ‘**’ 0.01 ‘*’ 0.05 ‘.’ 0.1 ‘’ 1

$Cucumber_Root$`14`

Analysis of Variance Table

Response: Value

| | Df | Sum Sq | Mean Sq | F value | Pr(>F) |
|---|---|---|---|---|---|
| Treatment | 3 | 0.047 | 0.01572 | 0.0078 | 0.999 |
| Residuals | 24 | 48.077 | 2.00321 | | |

***$Cucumber_Shoot***

$Cucumber_Shoot$`0`

Analysis of Variance Table

Response: Value

| | Df | Sum Sq | Mean Sq | F value | Pr(>F) |
|---|---|---|---|---|---|
| Treatment | 3 | 0.13627 | 0.045422 | 1.2706 | 0.3069 |
| Residuals | 24 | 0.85798 | 0.035749 | | |

$Cucumber_Shoot$`2`

Analysis of Variance Table

Response: Value

| | Df | Sum Sq | Mean Sq | F value | Pr(>F) | |
|---|---|---|---|---|---|---|
| Treatment | 3 | 0.10058 | 0.033525 | 0.43 | 0.7334 | |
| Residuals | 24 | 1.87115 | 0.077964 | | | |

$Cucumber_Shoot$`5`

Analysis of Variance Table

Response: Value

| | Df | Sum Sq | Mean Sq | F value | Pr(>F) | |
|---|---|---|---|---|---|---|
| Treatment | 3 | 0.6591 | 0.21968 | 1.0291 | 0.3974 | |
| Residuals | 24 | 5.1235 | 0.21348 | | | |

$Cucumber_Shoot$`7`

Analysis of Variance Table

Response: Value

| | Df | Sum Sq | Mean Sq | F value | Pr(>F) | |
|---|---|---|---|---|---|---|
| Treatment | 3 | 2.0097 | 0.66988 | 4.5094 | 0.01205 | * |
| Residuals | 24 | 3.5653 | 0.14855 | | | |

---

Signif. codes: 0 '***' 0.001 '**' 0.01 '*' 0.05 '.' 0.1 '' 1

$Cucumber_Shoot$`9`

Analysis of Variance Table

Response: Value

| | Df | Sum Sq | Mean Sq | F value | Pr(>F) | |
|---|---|---|---|---|---|---|
| Treatment | 3 | 5.7274 | 1.90915 | 3.1166 | 0.04492 | * |
| Residuals | 24 | 14.7016 | 0.61257 | | | |

---

Signif. codes: 0 '***' 0.001 '**' 0.01 '*' 0.05 '.' 0.1 '' 1

$Cucumber_Shoot$`12`

Analysis of Variance Table

Response: Value

| | Df | Sum Sq | Mean Sq | F value | Pr(>F) | |
|---|---|---|---|---|---|---|
| Treatment | 3 | 38.743 | 12.9144 | 12.924 | 3.16E-05 | *** |
| Residuals | 24 | 23.982 | 0.9993 | | | |

---

Signif. codes: 0 '***' 0.001 '**' 0.01 '*' 0.05 '.' 0.1 '' 1

$Cucumber_Shoot$`14`

Analysis of Variance Table

Response: Value

| | Df | Sum Sq | Mean Sq | F value | Pr(>F) |
|---|---|---|---|---|---|

| | | | | | | |
|---|---|---|---|---|---|---|
| Treatment | 3 | 24.109 | 8.0364 | 4.0799 | 0.01785 | * |
| Residuals | 24 | 47.274 | 1.9697 | | | |

---

Signif. codes: 0 ‘***’ 0.001 ‘**’ 0.01 ‘*’ 0.05 ‘.’ 0.1 ‘’ 1

***$Maize_Root***

$Maize_Root$`0`

Analysis of Variance Table

Response: Value

| | Df | Sum Sq | Mean Sq | F value | Pr(>F) | |
|---|---|---|---|---|---|---|
| Treatment | 3 | 0.23216 | 0.077386 | 3.2617 | 0.03895 | * |
| Residuals | 24 | 0.56942 | 0.023726 | | | |

---

Signif. codes: 0 ‘***’ 0.001 ‘**’ 0.01 ‘*’ 0.05 ‘.’ 0.1 ‘’ 1

$Maize_Root$`2`

Analysis of Variance Table

Response: Value

| | Df | Sum Sq | Mean Sq | F value | Pr(>F) |
|---|---|---|---|---|---|
| Treatment | 3 | 0.01743 | 0.00581 | 0.1591 | 0.9228 |
| Residuals | 24 | 0.87641 | 0.036517 | | |

$Maize_Root$`4`

Analysis of Variance Table

Response: Value

| | Df | Sum Sq | Mean Sq | F value | Pr(>F) |
|---|---|---|---|---|---|
| Treatment | 3 | 0.10164 | 0.033882 | 0.4221 | 0.7388 |
| Residuals | 24 | 1.92641 | 0.080267 | | |

$Maize_Root$`7`

Analysis of Variance Table

Response: Value

| | Df | Sum Sq | Mean Sq | F value | Pr(>F) |
|---|---|---|---|---|---|
| Treatment | 3 | 0.09082 | 0.030273 | 0.251 | 0.8599 |
| Residuals | 24 | 2.8952 | 0.120633 | | |

$Maize_Root$`9`

Analysis of Variance Table

Response: Value

| | Df | Sum Sq | Mean Sq | F value | Pr(>F) |
|---|---|---|---|---|---|
| Treatment | 3 | 0.23271 | 0.07757 | 0.6071 | 0.6168 |
| Residuals | 24 | 3.06642 | 0.12777 | | |

$Maize_Root$`11`
Analysis of Variance Table

Response: Value

| | Df | Sum Sq | Mean Sq | F value | Pr(>F) | |
|---|---|---|---|---|---|---|
| Treatment | 3 | 0.6493 | 0.21643 | 0.7132 | 0.5537 | |
| Residuals | 24 | 7.2836 | 0.30348 | | | |

$Maize_Root$`14`
Analysis of Variance Table

Response: Value

| | Df | Sum Sq | Mean Sq | F value | Pr(>F) | |
|---|---|---|---|---|---|---|
| Treatment | 3 | 3.8595 | 1.28649 | 3.4623 | 0.03204 | * |
| Residuals | 24 | 8.9176 | 0.37157 | | | |

---
Signif. codes: 0 '***' 0.001 '**' 0.01 '*' 0.05 '.' 0.1 '' 1

***$Maize_Shoot***
$Maize_Shoot$`0`
Analysis of Variance Table

Response: Value

| | Df | Sum Sq | Mean Sq | F value | Pr(>F) | |
|---|---|---|---|---|---|---|
| Treatment | 3 | 1.5313 | 0.51043 | 5.4837 | 0.005151 | ** |
| Residuals | 24 | 2.234 | 0.09308 | | | |

---
Signif. codes: 0 '***' 0.001 '**' 0.01 '*' 0.05 '.' 0.1 '' 1

$Maize_Shoot$`2`
Analysis of Variance Table

Response: Value

| | Df | Sum Sq | Mean Sq | F value | Pr(>F) |
|---|---|---|---|---|---|
| Treatment | 3 | 0.18 | 0.06001 | 0.1548 | 0.9256 |
| Residuals | 24 | 9.3068 | 0.38778 | | |

$Maize_Shoot$`4`
Analysis of Variance Table

Response: Value

| | Df | Sum Sq | Mean Sq | F value | Pr(>F) |
|---|---|---|---|---|---|
| Treatment | 3 | 0.5687 | 0.18956 | 0.2706 | 0.846 |
| Residuals | 24 | 16.8145 | 0.7006 | | |

$Maize_Shoot$`7`
Analysis of Variance Table

Response: Value

| | Df | Sum Sq | Mean Sq | F value | Pr(>F) |
|---|---|---|---|---|---|
| Treatment | 3 | 3.3601 | 1.12 | 0.8964 | 0.4574 |
| Residuals | 24 | 29.987 | 1.2495 | | |

$Maize_Shoot$`9`
Analysis of Variance Table

Response: Value

| | Df | Sum Sq | Mean Sq | F value | Pr(>F) |
|---|---|---|---|---|---|
| Treatment | 3 | 1.054 | 0.35149 | 0.252 | 0.8591 |
| Residuals | 24 | 33.481 | 1.39505 | | |

$Maize_Shoot$`11`
Analysis of Variance Table

Response: Value

| | Df | Sum Sq | Mean Sq | F value | Pr(>F) |
|---|---|---|---|---|---|
| Treatment | 3 | 0.956 | 0.31874 | 0.1246 | 0.9446 |
| Residuals | 24 | 61.388 | 2.55782 | | |

$Maize_Shoot$`14`
Analysis of Variance Table

Response: Value

| | Df | Sum Sq | Mean Sq | F value | Pr(>F) |
|---|---|---|---|---|---|
| Treatment | 3 | 16.826 | 5.6085 | 1.3295 | 0.2881 |
| Residuals | 24 | 101.247 | 4.2186 | | |

***$Tomato_Root***

$Tomato_Root$`0`
Analysis of Variance Table

Response: Value

| | Df | Sum Sq | Mean Sq | F value | Pr(>F) |
|---|---|---|---|---|---|
| Treatment | 3 | 0.000119 | 3.98E-05 | 0.8655 | 0.4725 |
| Residuals | 24 | 0.001102 | 4.59E-05 | | |

$Tomato_Root$`1`
Analysis of Variance Table

Response: Value

| | Df | Sum Sq | Mean Sq | F value | Pr(>F) |
|---|---|---|---|---|---|
| Treatment | 3 | 0.000114 | 3.81E-05 | 1.4958 | 0.241 |
| Residuals | 24 | 0.000611 | 2.55E-05 | | |

$Tomato_Root$`3`
Analysis of Variance Table

| | | | | | | | | |
|---|---|---|---|---|---|---|---|---|
| Response: | Value | | | | | | | |
| | Df | Sum Sq | Mean Sq | F value | Pr(>F) | | | |
| Treatment | 3 | 0.001362 | 0.000454 | 5.8439 | 0.003813 | ** | | |
| Residuals | 24 | 0.001864 | 7.77E-05 | | | | | |
| --- | | | | | | | | |
| Signif. | codes: | 0 | ‘***’ | 0.001 | ‘**’ | | 0.01 | ‘*’ |
| | | 0.05 | ‘.’ | 0.1 | ‘’ | | 1 | |
| $Tomato_Root$`7` | | | | | | | | |
| Analysis | of | Variance | Table | | | | | |
| | | | | | | | | |
| Response: | Value | | | | | | | |
| | Df | Sum Sq | Mean Sq | F value | Pr(>F) | | | |
| Treatment | 3 | 0.000483 | 0.000161 | 0.2248 | 0.8782 | | | |
| Residuals | 24 | 0.017187 | 0.000716 | | | | | |
| | | | | | | | | |
| $Tomato_Root$`9` | | | | | | | | |
| Analysis | of | Variance | Table | | | | | |
| | | | | | | | | |
| Response: | Value | | | | | | | |
| | Df | Sum Sq | Mean Sq | F value | Pr(>F) | | | |
| Treatment | 3 | 0.01659 | 0.00553 | 3.0016 | 0.05036 | . | | |
| Residuals | 24 | 0.044216 | 0.001842 | | | | | |
| --- | | | | | | | | |
| Signif. | codes: | 0 | ‘***’ | 0.001 | ‘**’ | | 0.01 | ‘*’ |
| | | 0.05 | ‘.’ | 0.1 | ‘’ | | 1 | |
| $Tomato_Root$`11` | | | | | | | | |
| Analysis | of | Variance | Table | | | | | |
| | | | | | | | | |
| Response: | Value | | | | | | | |
| | Df | Sum Sq | Mean Sq | F value | Pr(>F) | | | |
| Treatment | 3 | 0.033204 | 0.011068 | 2.0434 | 0.1346 | | | |
| Residuals | 24 | 0.129995 | 0.005417 | | | | | |
| | | | | | | | | |
| $Tomato_Root$`14` | | | | | | | | |
| Analysis | of | Variance | Table | | | | | |
| | | | | | | | | |
| Response: | Value | | | | | | | |
| | Df | Sum Sq | Mean Sq | F value | Pr(>F) | | | |
| Treatment | 3 | 0.19794 | 0.065979 | 3.7565 | 0.02417 | * | | |
| Residuals | 24 | 0.42154 | 0.017564 | | | | | |
| --- | | | | | | | | |
| Signif. | codes: | 0 | ‘***’ | 0.001 | ‘**’ | | 0.01 | ‘*’ |
| | | 0.05 | ‘.’ | 0.1 | ‘’ | | 1 | |
| | | | | | | | | |
| ***$Tomato_Shoot*** | | | | | | | | |
| $Tomato_Shoot$`0` | | | | | | | | |
| Analysis | of | Variance | Table | | | | | |
| | | | | | | | | |
| Response: | Value | | | | | | | |
| | Df | Sum Sq | Mean Sq | F value | Pr(>F) | | | |

Treatment 3 0.005352 0.001784 1.4448 0.2545
Residuals 24 0.029636 0.001235

$Tomato_Shoot$`1`
Analysis of Variance Table

Response: Value

| | Df | Sum Sq | Mean Sq | F value | Pr(>F) | |
|---|---|---|---|---|---|---|
| Treatment | 3 | 0.007618 | 0.002539 | 2.4634 | 0.08688 | . |
| Residuals | 24 | 0.02474 | 0.001031 | | | |

---
Signif. codes: 0 ‘***’ 0.001 ‘**’ 0.01 ‘*’
0.05 ‘.’ 0.1 ‘’ 1

$Tomato_Shoot$`3`
Analysis of Variance Table

Response: Value

| | Df | Sum Sq | Mean Sq | F value | Pr(>F) |
|---|---|---|---|---|---|
| Treatment | 3 | 0.007569 | 0.002523 | 2.1427 | 0.1212 |
| Residuals | 24 | 0.02826 | 0.001178 | | |

$Tomato_Shoot$`7`
Analysis of Variance Table

Response: Value

| | Df | Sum Sq | Mean Sq | F value | Pr(>F) | |
|---|---|---|---|---|---|---|
| Treatment | 3 | 0.37981 | 0.126603 | 17.464 | 3.14E-06 | *** |
| Residuals | 24 | 0.17398 | 0.007249 | | | |

---
Signif. codes: 0 ‘***’ 0.001 ‘**’ 0.01 ‘*’
0.05 ‘.’ 0.1 ‘’ 1

$Tomato_Shoot$`9`
Analysis of Variance Table

Response: Value

| | Df | Sum Sq | Mean Sq | F value | Pr(>F) | |
|---|---|---|---|---|---|---|
| Treatment | 3 | 1.86612 | 0.62204 | 25.954 | 1.04E-07 | *** |
| Residuals | 24 | 0.57521 | 0.02397 | | | |

---
Signif. codes: 0 ‘***’ 0.001 ‘**’ 0.01 ‘*’
0.05 ‘.’ 0.1 ‘’ 1

$Tomato_Shoot$`11`
Analysis of Variance Table

Response: Value

| | Df | Sum Sq | Mean Sq | F value | Pr(>F) | |
|---|---|---|---|---|---|---|
| Treatment | 3 | 10.011 | 3.337 | 51.163 | 1.41E-10 | *** |
| Residuals | 24 | 1.5654 | 0.0652 | | | |

---
Signif. codes: 0 ‘***’ 0.001 ‘**’ 0.01 ‘*’

| | | | | | | |
|---|---|---|---|---|---|---|
| | | 0.05 ‘.’ | | 0.1 ‘’ | | 1 |

$Tomato_Shoot$`14`

Analysis of Variance Table

Response: Value

| | Df | Sum Sq | Mean Sq | F value | Pr(>F) | |
|---|---|---|---|---|---|---|
| Treatment | 3 | 26.2742 | 8.7581 | 34.491 | 7.27E-09 | *** |
| Residuals | 24 | 6.0941 | 0.2539 | | | |

---

Signif. codes: 0 ‘***’ 0.001 ‘**’ 0.01 ‘*’ 0.05 ‘.’ 0.1 ‘’ 1

**Tukey.HSD multiple comparison on treatments**

| ***Between treatments differences*** | BR | | | | | | |
|---|---|---|---|---|---|---|---|
| ***Treatment effect*** | 0 | 2 | 4 | 7 | 9 | 11 | 14 |
| Tukey | P = 0.244 | P = 0.127 | P = 0.301 | P = 0.077 | P = 0.518 | P = 0.012 | P = 0.004 |
| C | a | a | a | a | a | ab | b |
| -P | a | a | a | a | a | ab | a |
| -Fe | a | a | a | a | a | b | b |
| -P/-Fe | a | a | a | a | a | a | ab |

| ***Between treatments differences*** | BS | | | | | | |
|---|---|---|---|---|---|---|---|
| ***Treatment effect*** | 0 | 2 | 4 | 7 | 9 | 11 | 14 |
| Tukey | P = 0.018 | P = 0.363 | P = 0.927 | P = 0.978 | P = 0.307 | P = 0.157 | P = 0.288 |
| C | a | a | a | a | a | a | a |
| -P | b | a | a | a | a | a | a |
| -Fe | ab | a | a | a | a | a | a |
| -P/-Fe | ab | a | a | a | a | a | a |

| ***Between treatments differences*** | CR --> not to test (P=0.363) | | | | | | |
|---|---|---|---|---|---|---|---|
| ***Treatment effect*** | 0 | 2 | 5 | 7 | 9 | 12 | 14 |
| Tukey | P = 0.320 | P = 0.715 | P = 0.471 | P = 0.094 | P = 0.957 | P = 0.036 | P = 0.999 |
| C | a | a | a | a | a | a | a |
| -P | a | a | a | a | a | a | a |
| -Fe | a | a | a | a | a | a | a |
| -P/-Fe | a | a | a | a | a | a | a |

| ***Between treatments differences*** | CS | | | | | | |
|---|---|---|---|---|---|---|---|
| ***Treatment effect*** | 0 | 2 | 5 | 7 | 9 | 12 | 14 |
| Tukey | P = 0.307 | P = 0.733 | P = 0.397 | P = 0.012 | P = 0.045 | P < 0.001 | P = 0.018 |
| C | a | a | a | a | a | a | ab |
| -P | a | a | a | ab | a | b | ab |
| -Fe | a | a | a | ab | a | b | a |
| -P/-Fe | a | a | a | b | a | b | b |

| ***Between treatments differences*** | MR | | | | | | |
|---|---|---|---|---|---|---|---|
| ***Treatment effect*** | 0 | 2 | 4 | 7 | 9 | 11 | 14 |
| Tukey | P = 0.039 | P = 0.923 | P = 0.739 | P = 0.860 | P = 0.617 | P = 0.553 | P = 0.032 |
| C | a | a | a | a | a | a | b |
| -P | ab | a | a | a | a | a | ab |
| -Fe | b | a | a | a | a | a | ab |
| -P/-Fe | ab | a | a | a | a | a | a |

| ***Between treatments differences*** | MS --> not to test (P=0.103) | | | | | | |
|---|---|---|---|---|---|---|---|
| ***Treatment effect*** | 0 | 2 | 4 | 7 | 9 | 11 | 14 |
| Tukey | P = 0.005 | P = 0.926 | P = 0.846 | P = 0.457 | P = 0.859 | P = 0.945 | P = 0.288 |
| C | a | a | a | a | a | a | a |
| -P | ab | a | a | a | a | a | a |
| -Fe | b | a | a | a | a | a | a |

| | | | | | | | |
|---|---|---|---|---|---|---|---|
| -P/-Fe | b | a | a | a | a | a | a |

***Between treatments differences*** TR --> not to test (P=0.339)

| ***Treatment effect*** | 0 | 1 | 3 | 7 | 9 | 11 | 14 |
|---|---|---|---|---|---|---|---|
| Tukey | P = 0.472 | P = 0.241 | P = 0.004 | P = 0.878 | P = 0.050 | P = 0.135 | P =0.024 |
| C | a | a | b | a | ab | a | a |
| -P | a | a | a | a | a | a | ab |
| -Fe | a | a | ab | a | ab | a | ab |
| -P/-Fe | a | a | ab | a | b | a | b |

***Between treatments differences*** TS

| ***Treatment effect*** | 0 | 1 | 3 | 7 | 9 | 11 | 14 |
|---|---|---|---|---|---|---|---|
| Tukey | P = 0.255 | P = 0.087 | P = 0.121 | P < 0.001 | P < 0.001 | P < 0.001 | P < 0.001 |
| C | a | a | a | a | a | a | a |
| -P | a | a | a | bc | bc | b | b |
| -Fe | a | a | a | b | b | bc | b |
| -P/-Fe | a | a | a | c | c | c | b |

**One-Way ANOVA on time**

***$Barley_Root***

$Barley_Root$C

Analysis of Variance Table

Response: Value

| | Df | Sum Sq | Mean Sq | F value | Pr(>F) | |
|---|---|---|---|---|---|---|
| Time | 9 | 1.62151 | 0.180167 | 34.424 | <2.2E-16 | *** |
| Residuals | 56 | 0.29309 | 0.005234 | | | |

---

Signif. codes: 0 ‘***’ 0.001 ‘**’ 0.01 ‘*’ 0.05 ‘.’ 0.1 ‘’ 1

$Barley_Root$Fe

Analysis of Variance Table

Response: Value

| | Df | Sum Sq | Mean Sq | F value | Pr(>F) | |
|---|---|---|---|---|---|---|
| Time | 6 | 1.51753 | 0.252922 | 11.529 | 1.37E-07 | *** |
| Residuals | 42 | 0.92138 | 0.021938 | | | |

---

Signif. codes: 0 ‘***’ 0.001 ‘**’ 0.01 ‘*’ 0.05 ‘.’ 0.1 ‘’ 1

$Barley_Root$P

Analysis of Variance Table

Response: Value

| | Df | Sum Sq | Mean Sq | F value | Pr(>F) | |
|---|---|---|---|---|---|---|
| Time | 6 | 3.8073 | 0.63456 | 28.566 | 2.50E-13 | *** |
| Residuals | 42 | 0.933 | 0.02221 | | | |

---

Signif. codes: 0 ‘***’ 0.001 ‘**’ 0.01 ‘*’ 0.05 ‘.’ 0.1 ‘’ 1

$Barley_Root$`P/Fe`

Analysis of Variance Table

Response: Value

| | Df | Sum Sq | Mean Sq | F value | Pr(>F) | |
|---|---|---|---|---|---|---|
| Time | 6 | 2.60344 | 0.43391 | 29.333 | 1.61E-13 | *** |
| Residuals | 42 | 0.62127 | 0.01479 | | | |

---

Signif. codes: 0 ‘***’ 0.001 ‘**’ 0.01 ‘*’ 0.05 ‘.’ 0.1 ‘’ 1

***$Barley_Shoot***

$Barley_Shoot$C

Analysis of Variance Table

Response: Value

| | Df | Sum Sq | Mean Sq | F value | Pr(>F) | |
|---|---|---|---|---|---|---|
| Time | 9 | 20.7437 | 2.30486 | 108.51 | <2.2E-16 | *** |
| Residuals | 56 | 1.1895 | 0.02124 | | | |

---

Signif. codes: 0 ‘***’ 0.001 ‘**’ 0.01 ‘*’ 0.05 ‘.’ 0.1 ‘’ 1

$Barley_Shoot$Fe

Analysis of Variance Table

Response: Value

| | Df | Sum Sq | Mean Sq | F value | Pr(>F) | |
|---|---|---|---|---|---|---|
| Time | 6 | 9.4204 | 1.57006 | 27.963 | 3.55E-13 | *** |
| Residuals | 42 | 2.3582 | 0.05615 | | | |

---

Signif. codes: 0 ‘***’ 0.001 ‘**’ 0.01 ‘*’ 0.05 ‘.’ 0.1 ‘’ 1

$Barley_Shoot$P

Analysis of Variance Table

Response: Value

| | Df | Sum Sq | Mean Sq | F value | Pr(>F) | |
|---|---|---|---|---|---|---|
| Time | 6 | 11.1636 | 1.8606 | 46.372 | <2.2E-16 | *** |
| Residuals | 42 | 1.6852 | 0.04012 | | | |

---

Signif. codes: 0 ‘***’ 0.001 ‘**’ 0.01 ‘*’ 0.05 ‘.’ 0.1 ‘’ 1

$Barley_Shoot$`P/Fe`

Analysis of Variance Table

Response: Value

| | Df | Sum Sq | Mean Sq | F value | Pr(>F) | |
|---|---|---|---|---|---|---|
| Time | 6 | 7.4657 | 1.24428 | 37.875 | 2.08E-15 | *** |
| Residuals | 42 | 1.3798 | 0.03285 | | | |

---

Signif. codes: 0 ‘***’ 0.001 ‘**’ 0.01 ‘*’ 0.05 ‘.’ 0.1 ‘’ 1

***$Cucumber_Root***

$Cucumber_Root$C

Analysis of Variance Table

Response: Value

| | Df | Sum Sq | Mean Sq | F value | Pr(>F) | |
|---|---|---|---|---|---|---|
| Time | 9 | 40.389 | 4.4876 | 10.698 | 1.93E-09 | *** |
| Residuals | 56 | 23.49 | 0.4195 | | | |

---

Signif. codes: 0 ‘***’ 0.001 ‘**’ 0.01 ‘*’ 0.05 ‘.’ 0.1 ‘’ 1

$Cucumber_Root$Fe

Analysis of Variance Table

Response: Value

| | Df | Sum Sq | Mean Sq | F value | Pr(>F) | |
|---|---|---|---|---|---|---|
| Time | 6 | 16.135 | 2.68914 | 2.9705 | 0.0165 | * |
| Residuals | 42 | 38.022 | 0.90529 | | | |

---

Signif. codes: 0 ‘***’ 0.001 ‘**’ 0.01 ‘*’ 0.05 ‘.’ 0.1 ‘’ 1

$Cucumber_Root$P

Analysis of Variance Table

Response: Value

| | Df | Sum Sq | Mean Sq | F value | Pr(>F) | |
|---|---|---|---|---|---|---|
| Time | 6 | 30.12 | 5.0201 | 8.7948 | 3.19E-06 | *** |
| Residuals | 42 | 23.974 | 0.5708 | | | |

---

Signif. codes: 0 ‘***’ 0.001 ‘**’ 0.01 ‘*’ 0.05 ‘.’ 0.1 ‘’ 1

$Cucumber_Root$`P/Fe`

Analysis of Variance Table

Response: Value

| | Df | Sum Sq | Mean Sq | F value | Pr(>F) | |
|---|---|---|---|---|---|---|
| Time | 6 | 15.053 | 2.5088 | 10.043 | 7.17E-07 | *** |
| Residuals | 42 | 10.491 | 0.24979 | | | |

---

Signif. codes: 0 ‘***’ 0.001 ‘**’ 0.01 ‘*’ 0.05 ‘.’ 0.1 ‘’ 1

***$Cucumber_Shoot***

$Cucumber_Shoot$C

Analysis of Variance Table

Response: Value

| | Df | Sum Sq | Mean Sq | F value | Pr(>F) | |
|---|---|---|---|---|---|---|
| Time | 9 | 238.001 | 26.4446 | 46.338 | <2.2E-16 | *** |
| Residuals | 56 | 31.959 | 0.5707 | | | |

---

Signif. codes: 0 ‘***’ 0.001 ‘**’ 0.01 ‘*’ 0.05 ‘.’ 0.1 ‘’ 1

$Cucumber_Shoot$Fe

Analysis of Variance Table

Response: Value

| | Df | Sum Sq | Mean Sq | F value | Pr(>F) | |
|---|---|---|---|---|---|---|
| Time | 6 | 109.458 | 18.2429 | 16.521 | 1.15E-09 | *** |
| Residuals | 42 | 46.378 | 1.1042 | | | |

---

Signif. codes: 0 ‘***’ 0.001 ‘**’ 0.01 ‘*’

0.05 ‘.’ 0.1 ‘’ 1

$Cucumber_Shoot$P

Analysis of Variance Table

Response: Value

| | Df | Sum Sq | Mean Sq | F value | Pr(>F) | |
|---|---|---|---|---|---|---|
| Time | 6 | 42.388 | 7.0647 | 28.261 | 2.98E-13 | *** |
| Residuals | 42 | 10.499 | 0.25 | | | |

---

Signif. codes: 0 ‘***’ 0.001 ‘**’ 0.01 ‘*’
0.05 ‘.’ 0.1 ‘’ 1

$Cucumber_Shoot$`P/Fe`

Analysis of Variance Table

Response: Value

| | Df | Sum Sq | Mean Sq | F value | Pr(>F) | |
|---|---|---|---|---|---|---|
| Time | 6 | 29.367 | 4.8945 | 23.96 | 4.29E-12 | *** |
| Residuals | 42 | 8.5797 | 0.2043 | | | |

---

Signif. codes: 0 ‘***’ 0.001 ‘**’ 0.01 ‘*’
0.05 ‘.’ 0.1 ‘’ 1

***$Maize_Root***

$Maize_Root$C

Analysis of Variance Table

Response: Value

| | Df | Sum Sq | Mean Sq | F value | Pr(>F) | |
|---|---|---|---|---|---|---|
| Time | 9 | 6.2784 | 0.6976 | 7.8048 | 2.42E-07 | *** |
| Residuals | 56 | 5.0053 | 0.08938 | | | |

---

Signif. codes: 0 ‘***’ 0.001 ‘**’ 0.01 ‘*’
0.05 ‘.’ 0.1 ‘’ 1

$Maize_Root$Fe

Analysis of Variance Table

Response: Value

| | Df | Sum Sq | Mean Sq | F value | Pr(>F) | |
|---|---|---|---|---|---|---|
| Time | 6 | 10.6715 | 1.77859 | 13.142 | 2.60E-08 | *** |
| Residuals | 42 | 5.6841 | 0.13534 | | | |

---

Signif. codes: 0 ‘***’ 0.001 ‘**’ 0.01 ‘*’
0.05 ‘.’ 0.1 ‘’ 1

$Maize_Root$P

Analysis of Variance Table

Response: Value

| | Df | Sum Sq | Mean Sq | F value | Pr(>F) | |
|---|---|---|---|---|---|---|
| Time | 6 | 9.5687 | 1.59478 | 7.5772 | 1.52E-05 | *** |

| | Df | Sum Sq | Mean Sq | F value | Pr(>F) | |
|---|---|---|---|---|---|---|
| Residuals | 42 | 8.8398 | 0.21047 | | | |

---

Signif. codes: 0 ‘***’ 0.001 ‘**’ 0.01 ‘*’ 0.05 ‘.’ 0.1 ‘’ 1

$Maize_Root$`P/Fe`

Analysis of Variance Table

Response: Value

| | Df | Sum Sq | Mean Sq | F value | Pr(>F) | |
|---|---|---|---|---|---|---|
| Time | 6 | 17.7294 | 2.9549 | 19.815 | 8.04E-11 | *** |
| Residuals | 42 | 6.2632 | 0.14912 | | | |

---

Signif. codes: 0 ‘***’ 0.001 ‘**’ 0.01 ‘*’ 0.05 ‘.’ 0.1 ‘’ 1

***$Maize_Shoot***

$Maize_Shoot$C

Analysis of Variance Table

Response: Value

| | Df | Sum Sq | Mean Sq | F value | Pr(>F) | |
|---|---|---|---|---|---|---|
| Time | 9 | 294.117 | 32.68 | 20.395 | 1.13E-14 | *** |
| Residuals | 56 | 89.729 | 1.602 | | | |

---

Signif. codes: 0 ‘***’ 0.001 ‘**’ 0.01 ‘*’ 0.05 ‘.’ 0.1 ‘’ 1

$Maize_Shoot$Fe

Analysis of Variance Table

Response: Value

| | Df | Sum Sq | Mean Sq | F value | Pr(>F) | |
|---|---|---|---|---|---|---|
| Time | 6 | 118.417 | 19.7361 | 26.254 | 9.93E-13 | *** |
| Residuals | 42 | 31.573 | 0.7517 | | | |

---

Signif. codes: 0 ‘***’ 0.001 ‘**’ 0.01 ‘*’ 0.05 ‘.’ 0.1 ‘’ 1

$Maize_Shoot$P

Analysis of Variance Table

Response: Value

| | Df | Sum Sq | Mean Sq | F value | Pr(>F) | |
|---|---|---|---|---|---|---|
| Time | 6 | 177.265 | 29.5442 | 14.348 | 8.09E-09 | *** |
| Residuals | 42 | 86.486 | 2.0592 | | | |

---

Signif. codes: 0 ‘***’ 0.001 ‘**’ 0.01 ‘*’ 0.05 ‘.’ 0.1 ‘’ 1

$Maize_Shoot$`P/Fe`

Analysis of Variance Table

Response: Value

| | | | | | | | |
|---|---|---|---|---|---|---|---|
| | Df | Sum Sq | Mean Sq | F value | Pr(>F) | | |
| Time | 6 | 148.132 | 24.6886 | 22.054 | 1.57E-11 | *** | |
| Residuals | 42 | 47.018 | 1.1195 | | | | |
| --- | | | | | | | |
| Signif. | codes: | 0 | ‘***’ | 0.001 | ‘**’ | 0.01 | ‘*’ |
| | | 0.05 | ‘.’ | 0.1 | ‘’ | 1 | |

***$Tomato_Root***

$Tomato_Root$C

| | | | | | | | |
|---|---|---|---|---|---|---|---|
| Analysis | of | Variance | Table | | | | |
| | | | | | | | |
| Response: | Value | | | | | | |
| | Df | Sum Sq | Mean Sq | F value | Pr(>F) | | |
| Time | 9 | 1.42763 | 0.158626 | 32.056 | <2.2E-16 | *** | |
| Residuals | 56 | 0.27711 | 0.004948 | | | | |
| --- | | | | | | | |
| Signif. | codes: | 0 | ‘***’ | 0.001 | ‘**’ | 0.01 | ‘*’ |
| | | 0.05 | ‘.’ | 0.1 | ‘’ | 1 | |

$Tomato_Root$Fe

| | | | | | | | |
|---|---|---|---|---|---|---|---|
| Analysis | of | Variance | Table | | | | |
| | | | | | | | |
| Response: | Value | | | | | | |
| | Df | Sum Sq | Mean Sq | F value | Pr(>F) | | |
| Time | 6 | 0.50577 | 0.084295 | 18.454 | 2.32E-10 | *** | |
| Residuals | 42 | 0.19185 | 0.004568 | | | | |
| --- | | | | | | | |
| Signif. | codes: | 0 | ‘***’ | 0.001 | ‘**’ | 0.01 | ‘*’ |
| | | 0.05 | ‘.’ | 0.1 | ‘’ | 1 | |

$Tomato_Root$P

| | | | | | | | |
|---|---|---|---|---|---|---|---|
| Analysis | of | Variance | Table | | | | |
| | | | | | | | |
| Response: | Value | | | | | | |
| | Df | Sum Sq | Mean Sq | F value | Pr(>F) | | |
| Time | 6 | 0.48877 | 0.081462 | 43.217 | <2.2E-16 | *** | |
| Residuals | 42 | 0.07917 | 0.001885 | | | | |
| --- | | | | | | | |
| Signif. | codes: | 0 | ‘***’ | 0.001 | ‘**’ | 0.01 | ‘*’ |
| | | 0.05 | ‘.’ | 0.1 | ‘’ | 1 | |

$Tomato_Root$`P/Fe`

| | | | | | | | |
|---|---|---|---|---|---|---|---|
| Analysis | of | Variance | Table | | | | |
| | | | | | | | |
| Response: | Value | | | | | | |
| | Df | Sum Sq | Mean Sq | F value | Pr(>F) | | |
| Time | 6 | 0.33276 | 0.05546 | 33.991 | 1.35E-14 | *** | |
| Residuals | 42 | 0.06853 | 0.001632 | | | | |
| --- | | | | | | | |
| Signif. | codes: | 0 | ‘***’ | 0.001 | ‘**’ | 0.01 | ‘*’ |
| | | 0.05 | ‘.’ | 0.1 | ‘’ | 1 | |

***$Tomato_Shoot***

$Tomato_Shoot$C

Analysis of Variance Table

Response: Value

| | Df | Sum Sq | Mean Sq | F value | Pr(>F) | |
|---|---|---|---|---|---|---|
| Time | 9 | 55.205 | 6.1339 | 55.473 | <2.2E-16 | *** |
| Residuals | 56 | 6.192 | 0.1106 | | | |

---

Signif. codes: 0 ‘***’ 0.001 ‘**’ 0.01 ‘*’ 0.05 ‘.’ 0.1 ‘’ 1

$Tomato_Shoot$Fe

Analysis of Variance Table

Response: Value

| | Df | Sum Sq | Mean Sq | F value | Pr(>F) | |
|---|---|---|---|---|---|---|
| Time | 6 | 4.4775 | 0.74625 | 16.879 | 8.45E-10 | *** |
| Residuals | 42 | 1.8569 | 0.04421 | | | |

---

Signif. codes: 0 ‘***’ 0.001 ‘**’ 0.01 ‘*’ 0.05 ‘.’ 0.1 ‘’ 1

$Tomato_Shoot$P

Analysis of Variance Table

Response: Value

| | Df | Sum Sq | Mean Sq | F value | Pr(>F) | |
|---|---|---|---|---|---|---|
| Time | 6 | 1.41583 | 0.235971 | 41.29 | 4.57E-16 | *** |
| Residuals | 42 | 0.24003 | 0.005715 | | | |

---

Signif. codes: 0 ‘***’ 0.001 ‘**’ 0.01 ‘*’ 0.05 ‘.’ 0.1 ‘’ 1

$Tomato_Shoot$`P/Fe`

Analysis of Variance Table

Response: Value

| | Df | Sum Sq | Mean Sq | F value | Pr(>F) | |
|---|---|---|---|---|---|---|
| Time | 6 | 0.5528 | 0.092133 | 19.101 | 1.39E-10 | *** |
| Residuals | 42 | 0.20258 | 0.004823 | | | |

---

Signif. codes: 0 ‘***’ 0.001 ‘**’ 0.01 ‘*’ 0.05 ‘.’ 0.1 ‘’ 1

**Tukey.HSD multiple comparison on time**

***Within treatments differences*** BR

| ***Time effect*** | Tukey | -11 | -7 | -3 | 0 | 2 | 4 | 7 | 9 | 11 | 14 |
|---|---|---|---|---|---|---|---|---|---|---|---|
| C | P < 0.001 | e | e | de | de | de | cd | cd | bc | b | a |
| -P | P < 0.001 | n.a. | n.a. | n.a. | d | cd | bcd | bc | b | b | a |
| -Fe | P < 0.001 | n.a. | n.a. | n.a. | c | c | bc | bc | b | bc | a |
| -P/-Fe | P < 0.001 | n.a. | n.a. | n.a. | e | de | cde | cd | bc | b | a |

***Within treatments differences*** BS

| ***Time effect*** | Tukey | -11 | -7 | -3 | 0 | 2 | 4 | 7 | 9 | 11 | 14 |
|---|---|---|---|---|---|---|---|---|---|---|---|
| C | P < 0.001 | f | f | ef | de | de | d | c | c | b | a |
| -P | P < 0.001 | n.a. | n.a. | n.a. | e | e | de | cd | bc | b | a |
| -Fe | P < 0.001 | n.a. | n.a. | n.a. | d | d | cd | bc | b | b | a |
| -P/-Fe | P < 0.001 | n.a. | n.a. | n.a. | d | d | cd | bc | b | a | a |

***Within treatments differences*** CR

| ***Time effect*** | Tukey | -13 | -7 | -2 | 0 | 2 | 5 | 7 | 9 | 12 | 14 |
|---|---|---|---|---|---|---|---|---|---|---|---|
| C | P < 0.001 | c | c | c | c | bc | bc | ab | abc | a | a |
| -P | P = 0.016 | n.a. | n.a. | n.a. | c | c | c | bc | c | a | ab |
| -Fe | P < 0.001 | n.a. | n.a. | n.a. | b | b | ab | ab | ab | ab | a |
| -P/-Fe | P < 0.001 | n.a. | n.a. | n.a. | b | b | b | b | b | b | a |

***Within treatments differences*** CS

| ***Time effect*** | Tukey | -13 | -7 | -2 | 0 | 2 | 5 | 7 | 9 | 12 | 14 |
|---|---|---|---|---|---|---|---|---|---|---|---|
| C | P < 0.001 | d | d | cd | cd | cd | bc | b | b | a | a |
| -P | P < 0.001 | n.a. | n.a. | n.a. | e | de | cd | bc | bc | b | a |
| -Fe | P < 0.001 | n.a. | n.a. | n.a. | d | cd | cd | cd | bc | ab | a |
| -P/-Fe | P < 0.001 | n.a. | n.a. | n.a. | c | bc | bc | b | b | a | a |

| ***Within treatments differences*** | MR | | | | | | | | | | |
|---|---|---|---|---|---|---|---|---|---|---|---|
| ***Time effect*** | Tukey | -17 | -7 | -3 | 0 | 2 | 4 | 7 | 9 | 11 | 14 |
| C | P < 0.001 | d | d | cd | bcd | bcd | bcd | bcd | abc | ab | a |
| -P | P < 0.001 | n.a. | n.a. | n.a. | b | b | b | b | b | b | a |
| -Fe | P < 0.001 | n.a. | n.a. | n.a. | d | cd | cd | cd | bc | ab | a |
| -P/-Fe | P < 0.001 | n.a. | n.a. | n.a. | c | bc | bc | bc | b | b | a |

| ***Within treatments differences*** | MS | | | | | | | | | | |
|---|---|---|---|---|---|---|---|---|---|---|---|
| ***Time effect*** | Tukey | -17 | -7 | -3 | 0 | 2 | 4 | 7 | 9 | 11 | 14 |
| C | P < 0.001 | d | d | d | cd | cd | bcd | bcd | bc | b | a |
| -P | P < 0.001 | n.a. | n.a. | n.a. | c | bc | bc | bc | bc | b | a |
| -Fe | P < 0.001 | n.a. | n.a. | n.a. | e | de | cde | cd | bc | b | a |
| -P/-Fe | P < 0.001 | n.a. | n.a. | n.a. | d | cd | cd | cd | bc | b | a |

| ***Within treatments differences*** | TR | | | | | | | | | | |
|---|---|---|---|---|---|---|---|---|---|---|---|
| ***Time effect*** | Tukey | -14 | -7 | -3 | 0 | 1 | 3 | 7 | 9 | 11 | 14 |
| C | P < 0.001 | d | cd | d | cd | cd | cd | cd | c | b | a |
| -P | P < 0.001 | n.a. | n.a. | n.a. | d | d | cd | c | b | b | a |
| -Fe | P < 0.001 | n.a. | n.a. | n.a. | d | d | cd | cd | bc | ab | a |
| -P/-Fe | P < 0.001 | n.a. | n.a. | n.a. | d | d | cd | bc | b | a | a |

| ***Within treatments differences*** | TS | | | | | | | | | | |
|---|---|---|---|---|---|---|---|---|---|---|---|
| ***Time effect*** | Tukey | -14 | -7 | -3 | 0 | 1 | 3 | 7 | 9 | 11 | 14 |
| C | P < 0.001 | d | d | d | d | d | d | cd | c | b | a |
| -P | P < 0.001 | n.a. | n.a. | n.a. | d | cd | bc | b | a | a | a |
| -Fe | P < 0.001 | n.a. | n.a. | n.a. | d | d | cd | cd | bc | ab | a |
| -P/-Fe | P < 0.001 | n.a. | n.a. | n.a. | c | c | bc | ab | ab | a | a |
